# The global, regional, and national burden of appendicitis in 204 countries and territories, 1990–2019: a systematic analysis from the Global Burden of Disease Study 2019

**DOI:** 10.1186/s12876-023-02678-7

**Published:** 2023-02-22

**Authors:** Linjing Guan, Zhen Liu, Guangdong Pan, Bulin Zhang, Yongrong Wu, Tao Gan, Guoqing Ouyang

**Affiliations:** 1 Department of Abdominal Ultrasound, Liuzhou People’s Hospital Affiliated to Guangxi Medical University, Liuzhou, Guangxi China; 2 Department of Hepatobiliary Surgery, Liuzhou People’s Hospital Affiliated to Guangxi Medical University, Liuzhou, Guangxi China; 3 Department of General Surgery, Liuzhou People’s Hospital Affiliated to Guangxi Medical University, Liuzhou, 545006 Guangxi China

**Keywords:** Appendicitis, Global Burden of Disease, Prevalence, Incidence, Years lived with disability

## Abstract

**Background:**

Appendicitis is the most common abdominal surgical emergency worldwide, and its burden has been changing. We report the level and trends of appendicitis prevalence, and incidence; and years lived with disability (YLD) in 204 countries and territories from 1990 to 2019, based on data from the Global Burden of Diseases, Injuries, and Risk Factors Study (GBD) 2019.

**Methods:**

The numbers and age-standardized prevalence, incidence, and YLD rates per 100,000 population of appendicitis were estimated across regions and countries by age, sex, and sociodemographic index (SDI). All the estimates were reported with 95% uncertainty intervals (UIs).

**Results:**

Globally, the age-standardized prevalence and incidence rates of appendicitis in 2019 were 8.7 (95% UI 6.9 to 11.0) and 229.9 (95% UI 180.9 to 291.0) per 100,000 population, with increases of 20.8% (95% UI 18.9 to 23.0%) and 20.5% (95% UI 18.7 to 22.8%) from 1990 to 2019, respectively. Additionally, the age-standardized YLDs rate was 2.7 (95% UI 1.8 to 3.9) in 2019, with an increase of 20.4% (95% UI 16.2 to 25.1%) from 1990 to 2019. In 2019, the age-standardized prevalence, incidence, and YLD rates peaked in the 15-to-19-year age groups in both male and female individuals. However, no statistically significant differences were observed between the male and female individuals in all groups. Ethiopia, India, and Nigeria showed the largest increases in the age-standardized prevalence rate between 1990 and 2019. Generally, positive associations were found between the age-standardized YLD rates and SDI at the regional and national levels.

**Conclusions:**

Appendicitis remains a major public health challenge globally. Increasing awareness of appendicitis and its risk factors and the importance of early diagnosis and treatment is warranted to reduce its the burden.

**Supplementary Information:**

The online version contains supplementary material available at 10.1186/s12876-023-02678-7.

## Background

Appendicitis is the most common abdominal surgical emergency worldwide, and it can lead to serious complications, such as ileus, peritonitis, abscess, and even death, as well as significant costs to the healthcare system [1, 2]. The incidence of appendicitis is approximately 233 per 100,000 population per year, with a lifetime incidence risk ranging from 6.7 to 8.6% [3, 4]. Although Western countries have reported a decrease in its incidence in the mid-twentieth century, newly industrialized countries have shown an increasing trend in the twenty-first century [4–6]. With the increasing accuracy of acute appendicitis, ultrasound and computed tomography (CT) were the most common modalities and promote the use of antibiotics. For patients without high-risk CT findings, management of antibiotics first is suggested, and surgery can be recommended if antibiotic treatment fails [7].

In 2018, World Health Organization (WHO) disclosed estimates of the cause-specific years of life lost (YLL), years lived with disability (YLD), and disability-adjusted life years (DALYs) for appendicitis stratified by cause, age, and sex at the global, regional, and country levels from 2000 to 2016 [8]. However, no study addressing these data has been published. Recently, a systematic review reported the global incidence using data from population-based studies, but only some regional and country-level data were presented, and the burden in most countries around the world was unavailable [9]. The latest study reported the global incidence and mortality from appendicitis using data from the (Global Burden of Disease Study) [GBD], but the 21 GBD regions and 204 countries were not analyzed in this study; in addition, prevalence and YLDs were unavailable from this study [10]. Some national and regional studies have evaluated the incidence, prevalence, mortality, years of life lost (YLL), and DALY, however, there is no comprehensive, detailed data for all countries [4, 9, 11–14]. To date, the incidence, prevalence, and YLD and association with the sociodemographic index (SDI) in all countries have not been analyzed. Therefore, a comprehensive, comparable analysis of the appendicitis burden is warranted to aid policy makers and healthcare providers in developing successful strategies to reduce the burden of appendicitis.

In the present study, we report the prevalence, incidence, and YLD of appendicitis in the general population in 204 countries and territories at the global, regional, and national levels in terms of the number and age-standardized rates stratified by age, sex, and SDI from 1990 to 2019.

## Methods

### Overview

The Institute of Health Metrics and Evaluation (IHME) conducted the Global Burden of Disease Study (GBD) 2019, which involved 204 countries, seven super-regions and 21 regions from 1990 to 2019 [15]. The GBD 2019 systematically analyzed 369 diseases and injuries, 286 causes of death and 87 risk factors in 2019. The general methodology of the GBD 2019 conducted by the IHME and its main modifications compared with previous years have been described previously [15, 16]. Additional information on non-fatal estimates was available at https://vizhub.healthdata.org/gbd-compare/ and http://ghdx.healthdata.org/gbd-results-tool. GBD 2019 complied with the Guidelines for Accurate and Transparent Health Estimate Reporting (GATHER) statement [17].

### Case definitions and data sources

GBD 2019 defined appendicitis as inflammation of the appendix causing nausea, vomiting, and sharp pain in the right lower abdomen. The gold-standard treatment for appendicitis is surgery. Furthermore, appendicitis can lead to septic shock and other severe complications, including sepsis and even death [15]. Vital registration and verbal autopsy data from the cause of death (COD) database were used to estimate mortality from appendicitis. In GBD 2019, the appendicitis data were obtained from claims and hospital inpatient data, including Poland claims data and additional years of data from USA claims (years 2015–2016) and hospital discharges in Mexico, India, New Zealand, Sweden, Georgia, Ecuador, Botswana and southern sub-Saharan Africa (Additional file 1: Table S1). Garbage code redistribution and noise reduction data, combined with small sample sizes, resulting in unreasonable cause fractions were excluded in the estimation. Details on the data adjustment are shown in Additional file 1: Section 1 and Table S2 [15].

The incidence of appendicitis was estimated by GBD research based on 1412 site years. Notably, a site year, the unique combination of a calendar year and location, was defined as a country or other subnational geographical unit contributing data each year. Only 48 of 204 countries representing 16 of 21 GBD regions provided data to estimate the incidence of appendicitis [15]. The data sources used in estimating the burden of appendicitis in different countries can be found using the GBD 2019 data input source tool (http://ghdx.healthdata.org/gbd-2019/datainput-sources).

### Data processing and disease model

The IHME used DisMod-MR 2.1, a Bayesian meta-regression tool, to estimate the appendicitis incidence and prevalence by pooling the age, sex, region and year. The DisMod-MR 2.1 analytical process is shown in Additional file 1: Figure S1. DisMod-MR 2.1 can conduct age- integration; however, its performance decreased while integrating across wide age groups (e.g., all ages). In the DisMod-MR 2.1 model, the data were disaggregated by age to calculate a country’s age-pattern and then the calculated age pattern was applied to split aggregated all the age data (Additional file 1: Section 2) [15].

For appendicitis, the reference incidence data were adjusted using claims and hospital inpatient data. In the IHME’s prior setting in the DisMod model, remission was bounded from 25 to 27 (approximately 2 weeks) for all the age groups and excess mortality was capped at 0.31. Study covariates were used to adjust the incidence date derived from USA claims data for 2000 to better align with other incidence data points, which were more representative of the general population [15].

Additionally, the function in DisMod-MR 2.1 was utilized to calculate cause-specific mortality rates (CSMRs) resulting from our CODEm and CODcorrect analyses, and the CSMRs were matched with prevalence data points for the same geography [15]. The excess mortality rate was calculated to estimate priors by dividing the CSMR by the prevalence. Moreover, a country-level fiber (g per day) covariate was applied to the incidence, forcing a positive relationship with a lower bound of 0. The excess mortality, log-transformed and forced negative with an upper bound of 0 and a lower bound of − 1 was applied with a lag-distributed income (LDI) covariate. Similarly, the Healthcare Access and Quality Index (HAQI) covariate also forced a negative (− 2, 0) excess mortality value. In GBD 2019, the minimum coefficient of variation at the regional, super-regional, and global-levels was changed from 0.4 to 0.8 to improve the model fit against input data. Betas and exponentiated values (which can be interpreted as odds ratios) of predictive covariates are shown in the Additional file 1: Tables S3 and S4 [15].

### Severity and YLDs

International Classification of Diseases version 10 (ICD-10) codes were applied to identify appendicitis (K35-K38). The lay descriptions of sequelae highlighting major functional consequences and symptoms are the basis for the GBD disability weights (DWs). Only one disease sequela (severity level) in the data of GBD was evaluated for appendicitis, and the DW was 0.324 (95% CI 0.219 to 0.442). The DW is recognized as a factor that reveals the severity of a disease or condition on a scale from 0 (no disease) to 1 (death). Patients with severe appendicitis had severe pain in the stomach, felt nauseated and anxious and could not perform daily activities. The sequela-specific prevalence was multiplied by the severity-specific DWs to estimate the YLDs [15, 18, 19].

### Compilation of results

YLLs were calculated by multiplying the difference between the standard life expectancy for a given age and sum of deaths in each age group [20]. DALYs were calculated by summing the YLL and YLD [21, 22]. Uncertainty was estimated from multiple sources, such as the input data, measurement error corrections, and residual non-sampling error estimates. Uncertainty intervals (UIs) were defined as the 2.5 and 97.5 percentiles of the ordered draws. The flowcharts of estimation for appendicitis are shown in Additional file 1: Figure S2 [15].

Smoothing spline models were employed to determine the shape of the correlation curve between the appendicitis burden in terms of YLD and SDI for 21 regions and 204 countries and territories [23, 24]. The SDI is a value ranging from 0 (worst) to 1.0 (best) and was calculated from the total fertility rate among those aged younger than 25 years, with a mean education level for the population greater than 15 years, and lag-distributed income per capita (LDI) [25]. All the estimates of prevalence, incidence, and YLD was generated using by R software version 3.6.3 [26]. The differences between sexes were compared with unpaired t test. A *P* value < 0.05 was considered statistically significant.

### Patient and public involvement statement

This study used the data freely available from the GBD at http://ghdx.healthdata.org/gbd-results-tool. The data analyzed during this study could refer to Table 1 and Additional file 1: Tables S5-S7. In addition, data are available from the corresponding author on reasonable request. Patients were not involved in the design, recruitment or conduct of the study. Results of this study will be made publicly available through publication.

**Table 1:** Prevalent cases, incident cases, years lived with disability (YLD), and years of life lost (YLL) for appendicitis in 2019 for both sexes and percentage change of age-standardized rates (ASR) per 100,000 populations from 1990 to 2019 by Global Burden of Disease regions

	Prevalence (95% uncertainty interval)			Incidence (95% uncertainty interval)			YLDs (95% uncertainty interval)		
	Counts	ASR per 100,000 population (95% UI)	Percentage change in ASRs per 100,000 population (95% UI)	Counts	ASR per 100,000 population (95% UI)	Percentage change in ASRs per 100,000 population (95% UI)	Counts	ASR per 100,000 population (95% UI)	Percentage change in ASRs per 100,000 population (95% UI)
Global	672,203 (536,225 to 847,977)	8.7 (6.9 to 11)	20.8 (23 to 18.9)	17,698,765 (14,101,114 to 22,324,572)	229.9 (180.9 to 291)	20.5 (22.8 to 18.7)	211,113 (137,041 to 303,366)	2.7 (1.8 to 3.9)	20.4 (25.1 to 16.2)
Andean Latin America	21,347 (17,402 to 26,573)	32.5 (26.6 to 40.2)	− 30.7 (− 36.6 to − 22.2)	558,662 (456,610 to 696,711)	852.4 (697.7 to 1059.4)	− 30.5 (− 36.6 to − 22)	6569 (4287 to 9596)	10 (6.5 to 14.5)	− 30.7 (− 41.2 to − 17.9)
Australasia	2850 (2247 to 3629)	10.8 (8.4 to 14)	2.4 (− 2.1 to 6.4)	74,147 (58,363 to 94,560)	283.9 (220.1 to 369.3)	2.4 (− 2.1 to 6.4)	882 (546 to 1332)	3.4 (2 to 5.2)	2.4 (− 22.8 to 35.2)
Caribbean	5046 (3913 to 6473)	10.8 (8.3 to 13.9)	28.8 (25.3 to 32)	132,078 (102,639 to 169,890)	282.9 (219.4 to 361.9)	28.6 (25.1 to 31.8)	1574 (1023 to 2353)	3.4 (2.2 to 5)	28.3 (9.7 to 50.4)
Central Asia	8907 (6690 to 11,734)	9.4 (7 to 12.3)	1.5 (− 2 to 5.4)	233,029 (175,238 to 306,969)	245.3 (184.6 to 323.5)	1.5 (− 2.1 to 5.4)	2799 (1767 to 4212)	2.9 (1.8 to 4.4)	1.3 (− 13.5 to 17)
Central Europe	7814 (6451 to 9388)	8.4 (6.7 to 10.4)	7.6 (0.6 to 15.8)	203,543 (167,437 to 245,338)	220.3 (175.2 to 271.3)	7.5 (0.6 to 15.7)	2486 (1625 to 3576)	2.7 (1.7 to 3.9)	7.4 (− 3.7 to 20.2)
Central Latin America	34,366 (26,234 to 44,412)	13.6 (10.3 to 17.6)	16.1 (12.3 to 20.1)	900,312 (686,867 to 1,159,909)	358.2 (271.1 to 460.1)	15.4 (11.8 to 19.4)	10,795 (6781 to 16,177)	4.3 (2.7 to 6.4)	16.3 (6.1 to 27.3)
Central Sub-Saharan Africa	15,752 (11,998 to 20,404)	10.8 (8.5 to 13.8)	36.1 (30.6 to 42.4)	416,895 (315,480 to 538,242)	284.3 (224.9 to 361.7)	35.8 (30.3 to 42.2)	4900 (3015 to 7598)	3.4 (2.1 to 5.1)	36 (8 to 72.8)
East Asia	98,212 (77,893 to 120,704)	6.7 (5.3 to 8.3)	8.4 (2.7 to 14.7)	2,565,267 (2,034,140 to 3,149,307)	176.2 (138.5 to 220)	7.4 (1.8 to 13.6)	31,567 (20,685 to 45,211)	2.2 (1.4 to 3.1)	9.1 (1.5 to 18.2)
Eastern Europe	18,435 (14,229 to 23,497)	10.4 (7.8 to 13.6)	19.9 (17.3 to 22.8)	480,413 (370,619 to 612,175)	273.2 (202.6 to 354.6)	19.7 (17.1 to 22.5)	5795 (3710 to 8441)	3.3 (2.1 to 4.9)	19.7 (9.4 to 31.6)
Eastern Sub-Saharan Africa	22,041 (16,104 to 29,956)	4.7 (3.6 to 6.3)	46.5 (42.9 to 49.9)	591,822 (432,475 to 797,031)	127 (96.7 to 166.9)	45.7 (42.2 to 49)	6956 (4286 to 10,506)	1.5 (0.9 to 2.2)	45.5 (31.2 to 61.2)
High-income Asia Pacific	22,677 (17,978 to 28,337)	17.2 (13.1 to 22.1)	− 2.6 (− 7.2 to 2.3)	586,290 (463,318 to 730,184)	448.1 (339.7 to 572.3)	− 2.6 (− 7.2 to 2.5)	7111 (4538 to 10,230)	5.4 (3.4 to 7.9)	− 2.2 (− 11.7 to 8.9)
High-income North America	21,877 (19,671 to 24,295)	6.2 (5.5 to 6.9)	− 10.4 (− 21.8 to 4)	571,783 (514,543 to 636,513)	162.6 (144.9 to 182.1)	− 10.3 (− 21.7 to 4.1)	7057 (4756 to 9721)	2 (1.3 to 2.8)	− 8.9 (− 21.9 to 6.8)
North Africa and Middle East	66,665 (50,979 to 87,066)	10.4 (8 to 13.5)	48.2 (44.1 to 52.5)	1,745,503 (1,329,173 to 2,271,525)	271.6 (206.1 to 350.9)	48 (43.9 to 52.2)	20,714 (12,951 to 30,887)	3.2 (2 to 4.8)	46.5 (32.5 to 63)
Oceania	577 (444 to 755)	4.1 (3.2 to 5.4)	16.2 (12.2 to 21.1)	15,219 (11,665 to 19,748)	109.2 (85 to 140.3)	16.1 (12.1 to 21)	187 (118 to 279)	1.3 (0.9 to 2)	16.2 (12.2 to 21.1)
South Asia	196,227 (154,672 to 249,562)	10 (8 to 12.6)	53.5 (45.9 to 61)	5,214,009 (4,114,157 to 6,620,999)	266.7 (212.7 to 334.9)	51.8 (44.3 to 59.1)	60,854 (38,899 to 89,567)	3.1 (2 to 4.5)	52.5 (36.3 to 70.7)
Southeast Asia	43,365 (33,776 to 56,231)	6.3 (4.9 to 8.1)	34.2 (30.6 to 37.7)	1,146,656 (892,434 to 1,477,241)	166.5 (129.2 to 216.3)	33.9 (30.4 to 37.4)	13,780 (8689 to 20,227)	2 (1.3 to 2.9)	34.5 (20.4 to 51.4)
Southern Latin America	7736 (6046 to 9784)	11.7 (9 to 14.9)	49.8 (42 to 57.6)	201,955 (157,808 to 254,876)	305.5 (236.3 to 389.3)	49.6 (41.8 to 57.5)	2394 (1497 to 3585)	3.6 (2.2 to 5.4)	46.6 (14.1 to 86.4)
Southern Sub-Saharan Africa	5855 (4394 to 7749)	6.9 (5.2 to 9.1)	26.2 (22.4 to 30.6)	154,821 (116,584 to 203,979)	183 (138.5 to 241)	25.9 (22.1 to 30.2)	1837 (1152 to 2840)	2.2 (1.4 to 3.3)	25.1 (10 to 43.7)
Tropical Latin America	11,326 (9235 to 14,000)	5.1 (4.1 to 6.3)	28.9 (23.6 to 35.5)	298,011 (243,250 to 366,750)	135.6 (109.7 to 167.8)	27.8 (22.6 to 34.3)	3616 (2379 to 5269)	1.6 (1.1 to 2.4)	27.8 (17.7 to 39.9)
Western Europe	38,356 (31,229 to 47,142)	10.6 (8.5 to 13.6)	12.1 (6.6 to 18.3)	997,135 (806,633 to 1,229,567)	278.5 (220.2 to 352.8)	12.1 (6.6 to 18.3)	11,990 (7743 to 17,155)	3.3 (2.1 to 4.8)	12 (0.6 to 25.9)
Western Sub-Saharan Africa	22,773 (16,551 to 30,697)	4.5 (3.4 to 6)	48.7 (43.6 to 54)	611,213 (445,004 to 824,269)	120.1 (89.7 to 158.9)	47.6 (42.4 to 52.8)	7249 (4465 to 11,015)	1.4 (0.9 to 2.2)	47.9 (37 to 58.6)

## Results

### Global level

Globally, there were 672,203 (95% UI 536,225 to 847,977) prevalent cases of appendicitis, with an age-standardized prevalence rate of 8.7 (95% UI 6.9 to 11.0) per 100,000 population in 2019. The age-standardized prevalence rate was 7.2 (95% UI 5.7 to 9.1) per 100,000 population in 1990, and increased by 20.8% (95% UI 18.9 to 23.0%) from 1990 to 2019. Additionally, there were 17,698,765 (5% UI 14,101,114 to 22,324,572) incident appendicitis cases, with an age-standardized incidence rate of 229.9 (95% UI 180.9 to 291.0) per 100,000 populations in 2019. The age-standardized incidence rate was 190.7 (95% UI 149.6 to 240.6) per 100,000 population in 1990, with a 20.5% (95% UI 18.7 to 22.8%) increase from 1990 to 2019 (Table 1).

The global age-standardized YLD rate showed a steady trend from 1990 to 1997, and then increased from 1998 to 2019. Globally, in 2019, appendicitis accounted for 211,113 (95% UI 137,041 to 303,366) YLD, with an age-standardized YLD rate of 2.7 (95% UI 1.8 to 3.9) per 100,000 population. The age-standardized YLD rate was 2.3 (95% UI 1.5 to 3.2) per 100,000 population in 1990, with an increase of 20.4% (95% UI 16.2 to 25.1%) from 1990 to 2019 (Table 1).

### Regional level

**Fig. 1:**
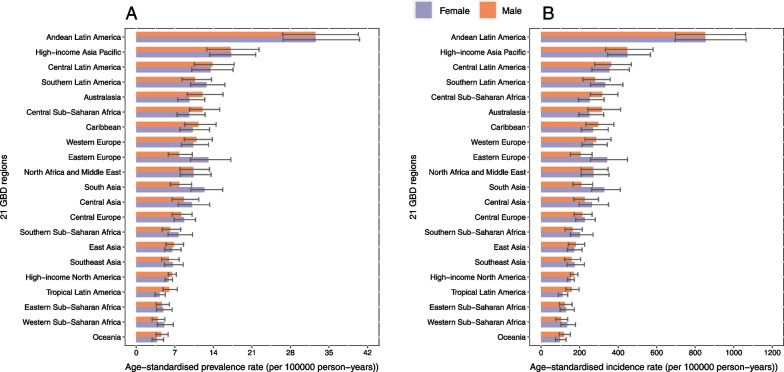
The age-standardized prevalence (**A**) and incidence (**B**) rate of appendicitis in 2019 for 21 GBD region, by sex

At the regional level, Andean Latin America (32.5 [95% UI 26.6 to 40.2]), High-income Asia Pacific (17.2 [95% UI 13.1 to 22.1]), and Central Latin America (13.6 [95% UI 10.3 to 17.6]) demonstrated the highest age-standardized prevalence rates of appendicitis per 100,000 population in 2019. By contrast, the lowest age-standardized prevalence rate was found in Oceania (4.1 [95% UI 3.2 to 5.4]), Western Sub-Saharan Africa (4.5 [95% UI 3.4 to 6.0]), and Eastern Sub-Saharan Africa (4.7 [95% UI 3.6 to 6.3]) in 2019 (Table 1, Fig. 1A).

The age-standardized incidence rates of appendicitis per 100,000 population were also found to be highest in Andean Latin America (852.4 [95% UI 697.7 to 1059.4]), High-income Asia Pacific (448.1 [95% UI 339.7 to 572.3]), and Central Latin America (358.2 [95% UI 271.1 to 460.1]), whereas Oceania (109.2 [95% UI 85.0 to 140.3]), Western Sub-Saharan Africa (120.1 [95% UI 89.7 to 158.9]) and Eastern Sub-Saharan Africa (127.0 [95% UI 96.7 to 166.9]) had the lowest age-standardized incidence rates (Table 1, Fig. 1B).

Andean Latin America (10.0 [95% UI 6.5 to 14.5]), High-income Asia Pacific (5.4 [95% UI 3.4 to 7.9]) and Central Latin America (4.3 [95% UI 2.7 to 6.4]) had the highest age-standardized YLD rates from appendicitis per 100,000 population in 2019, whereas Oceania (1.3 [95% UI 0.9 to 2.0]), Western Sub-Saharan Africa (1.4 [95% UI 0.9 to 2.2]) and Eastern Sub-Saharan Africa (1.5 [95% UI 0.9 to 2.2]) had the lowest age-standardized YLD rates among the GBD 2019 regions (Table 1, Additional file 2: Fig. S3).

The percentage change in the age-standardized prevalence rates varied across the 21 GBD regions from 1990 to 2019. South Asia (53.5% [95% UI 45.9 to 61.0%]), Southern Latin America (49.8% [95% UI 42.0 to 57.6%]), and Western Sub-Saharan Africa (48.7% [95% UI 43.6 to 54.0%]) showed the largest increasing trends, while Andean Latin America (− 30.7% [95% UI − 36.6 to − 22.2%]), High-income North America (− 10.4% [95% UI − 21.8 to − 4.0%]) and High-income Asia Pacific (− 2.6% [95% UI − 7.2 to − 2.3%]) showed the largest decreasing trends (Additional file 2: Fig. S4). The percentage change in the age-standardized incidence rates varied across the 21 GBD regions from 1990 to 2019. South Asia (51.8% [95% UI 44.3 to 59.1%]), Southern Latin America (49.6% [95% UI 41.8 to 57.5%]), and North Africa and Middle East (48.0% [95% UI 43.9 to 52.2%]) showed the largest increasing trends, while Andean Latin America (− 30.5% [95% UI − 36.6 to − 22.0%]), High-income North America (− 10.3% [95% UI − 21.7 to − 4.1%]) and High-income Asia Pacific (− 2.6% [95% UI − 7.2 to − 2.5%]) showed the largest decreasing trends (Additional file 2: Fig. S5).

The top three statistically significant increases in age-standardized YLD rates were observed in South Asia (52.5% [95% UI 36.3 to 70.7%]), Western Sub-Saharan Africa (47.9% [95% UI 37.0 to 58.6%]), and Southern Latin America (46.6% [95% UI 14.1 to 86.4%]), whereas the top three statistically significant decreasing trends were observed in Andean Latin America (− 30.7% [95% UI − 41.2 to − 17.9%]), High-income North America (− 8.9% [95% UI − 21.9 to 6.8%]), and High-income Asia Pacific (− 2.2% [95% UI − 11.7 to 8.9%]) (Additional file 2: Fig. S6).

The number of prevalent cases was increased by 1.6 times from 1990 409,125 (95% UI: 318,852 to 520,824) to 2019 672,203 (95% UI 536,225 to 847,977), but the regions contributing to the increase in 2019 varied (Additional file 2: Fig. S7). The number of incident cases was also increased by 1.6 times from 10,821,656 (95% UI: 8,420,122 to 13,838,792) in 1990 to 17,698,765 (95% UI: 14,101,114 to 22,324,572) in 2019, with differing contributions from GBD 2019 regions (Additional file 2: Fig. S8).

### National level

**Fig. 2:**
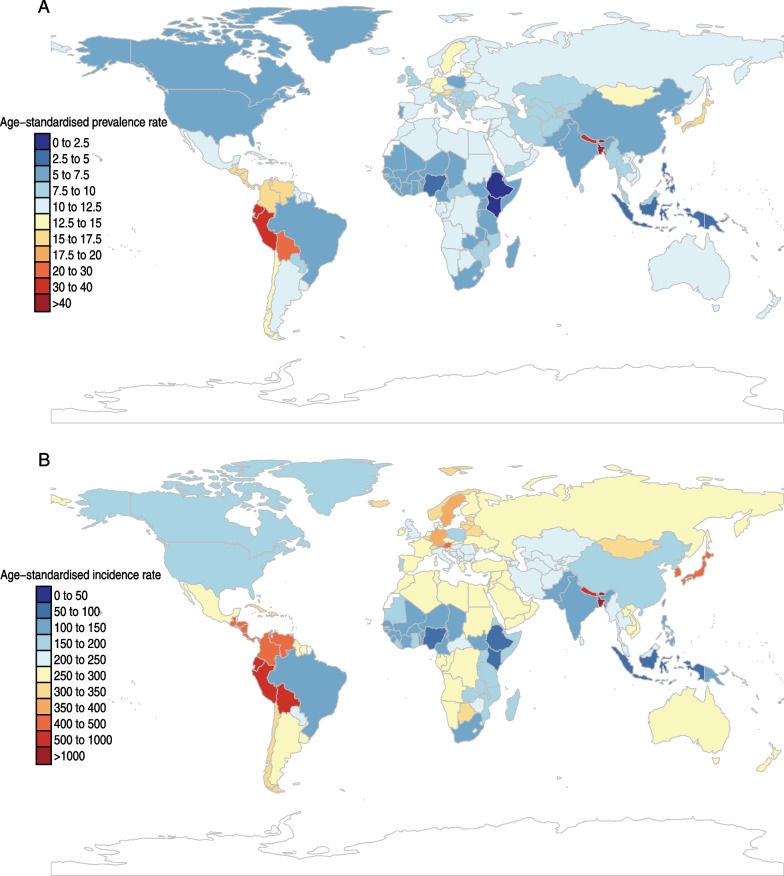
The global age-standardized prevalence (**A**) and incidence (**B**) rate of appendicitis per 100,000 population in 2019, by country and territory

At the national level, the age-standardized prevalence rate of appendicitis varied from 1.9 to 51.5 cases per 100,000 population. Bangladesh (51.5 [95% UI 41.3 to 64.1]), Bhutan (44.8 [95% UI 35.8 to 56.0]), and Peru (33.6 [95% UI 26.4 to 43.2]) had the three highest age-standardized prevalence rates in 2019, whereas Ethiopia (1.9 [95% UI 1.3 to 2.6]), Kenya (2.3 [95% UI 1.7 to 3.2]) and Indonesia (3.4 [95% UI 2.5 to 4.5]) showed the lowest age-standardized rates (Additional file 1: Table S5, Fig. 2A).

The age-standardized incidence rates of appendicitis varied from 53.8 to 1349.8 cases per 100,000 population. In 2019, Bangladesh (1349.8 [95% UI 1092.2 to 1673.1]), Bhutan (1174.4 [95% UI 942.1 to 1459.6]), and Peru (879.7 [95% UI 688.0 to 1137.6]) had the highest age-standardized incidence rates. By contrast, Ethiopia (53.8 [95% UI 38.8 to 73.3]), Kenya (65.4 [95% UI 47.4 to 88.7]) and Indonesia (92.5 [95% UI 68.7 to 121.8]) had the lowest age-standardization rates (Additional file 1: Table S6, Fig. 2B).

Additionally, the age-standardized YLD rate of appendicitis in 2019 ranged from 0.6 to 15.7 cases per 100,000 population. The highest rates were found in Bangladesh (15.7 [95% UI 10.1 to 23.5]), Bhutan (13.6 [95% UI 8.8 to 20.4]), and Peru (10.4 [95% UI 6.6 to 15.6]), and the lowest rates were also found in Ethiopia (0.6 [95% UI 0.4 to 0.9]), Kenya (0.8 [95% UI 0.5 to 1.1]) and Indonesia (1.1 [95% UI 0.7 to 1.6]) (Additional file 1: Table S7, Additional file 2: Fig. S9).

The percent changes in the age-standardized prevalence rates per 100,000 population from 1990 to 2019 differed substantially among 204 countries and territories. Ethiopia (207.6% [95% UI 178.6 to 243.7%]), India (105.6% [95% UI 98.5 to 113.5%]), and Nigeria (101.4% [95% UI 94.7 to 109.7%]) had the largest increasing trends over the 30 years. By contrast, Peru (− 44.3% [95% UI − 51.5 to − 33.8%]), Mongolia (− 39.7% [95% UI − 46.1 to − 30.6%]) and Guatemala (− 33.6% [95% UI − 42.8 to − 22.8%]) had the largest decreasing trends from 1990 to 2019 (Additional file 1: Table S5, Additional file 2: Fig. S10).

The percent changes in the age-standardized incidence rates between 1990 and 2019 also differed across all countries and territories. The largest increases were observed in Ethiopia (176.1% [95% UI 150.9 to 207.1%]), India (98.4% [95% UI 91.2 to 106.2%]) and Nigeria (96.3% [95% UI 90.2 to 104.1%]). The largest decreases during this period were found in Peru (− 44.2% [95% UI − 51.5 to − 33.6%]), Mongolia (− 39.8% [95% UI − 46.1 to − 30.7%]) and Guatemala (− 33.6% [95% UI − 42.9 to − 22.9%]) (Additional file 1: Table S6, Additional file 2: Fig. S11).

The largest increases in age-standardized YLD due to appendicitis per 100,000 population between 1990 and 2019 were in Ethiopia (207.4% [95% UI 178.6 to 243.7%]), India (101.4% [95% UI 88.2 to 113.6%]), and Nigeria (101.1% [95% UI 93.9 to 109.8%]). By contrast, Peru (− 44.3% [95% UI − 56.6 to − 29.0%]), Mongolia (− 39.8% [95% UI − 55.4 to − 18.6%]), and Guatemala (− 32.6% [95% UI − 50.5 to − 10.9%]) showed the largest decreases from 1990 to 2019 (Additional file 1: Table S7, Additional file 2: Fig. S12).

### Sex and age patterns

**Fig. 3:**
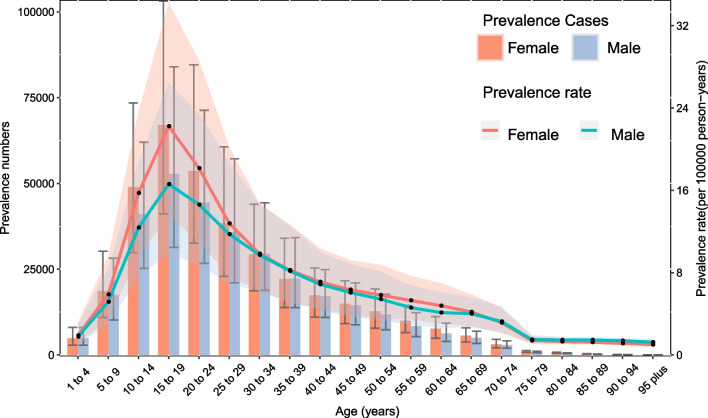
Global number and rates of prevalence for appendicitis per 100,000 population by age and sex, 2019. Shading indicates the 95% UIs for the prevalent rate

Globally, no statistically significant differences were observed in the prevalence between women and men in any age group. The number of prevalent cases increased with age and peaked in the 15-to 19-year age group for both males and female individuals, after this age, the overall trend declined. Additionally, the prevalence rate reached its peak in the 15- to 19-year age group and then decreased with age in both males and female individuals in 2019 (Fig. 3). No visible difference was noted between the incidence in males and female individuals in all age groups. The number of incident cases reached its highest level in the 15- to 19-year age group in both males and females individuals, after which a declining trend with increasing age was observed. In 2019, the incidence rate also peaked in the 15- to 19-year age group. After this age, the incidence rate then decreased with age in both males and female individuals (Additional file 2: Fig. S13). The pattern of YLDs by sex and age group was relatively similar to those of prevalence and incidence (Additional file 2: Fig. S14).

### Burden of appendicitis by SDI

**Fig. 4:**
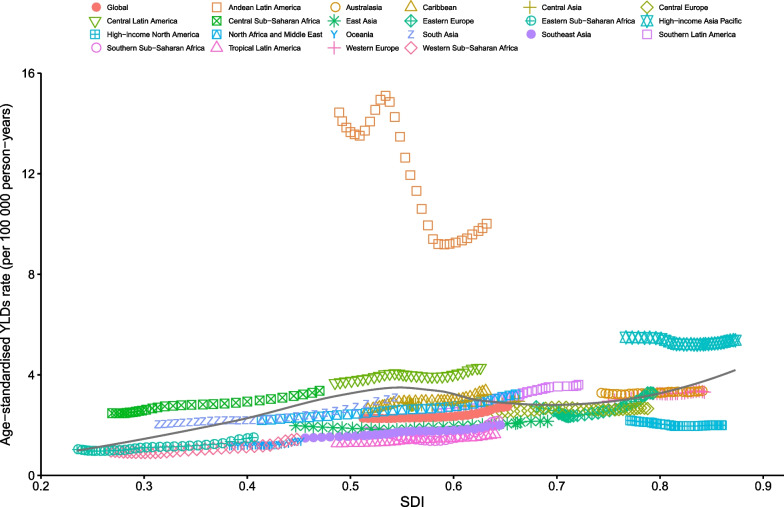
Age-standardized YLDs rates of appendicitis in 21 GBD regions by SDI, 1990–2019. Expected values based on Socio-demographic Index and disease rates in all locations are shown as the black line. YLDs, years lived with disability. GBD, Global Burden of Diseases, Injuries, and Risk Factors Study. SDI, Sociodemographic Index

At the regional level, a generally positive association was observed between the age-standardized YLD rate and SDI from 1990 to 2019. The lowest age-standardized YLD rate was observed when the SDI was approximately 0.23, showing an overall increasing trend with the SDI value. From 1990 to 2019, the observed burden was higher than the expected level in Andean Latin America, Central Sub-Saharan Africa, Central Latin America, and High-income Asia Pacific. By contrast, High-income North America, Western Sub-Saharan Africa, Southeast Asia, Tropical Latin America, East Asia, and global were below the expected level based on the SDI in all years (Fig. 4).

**Fig. 5:**
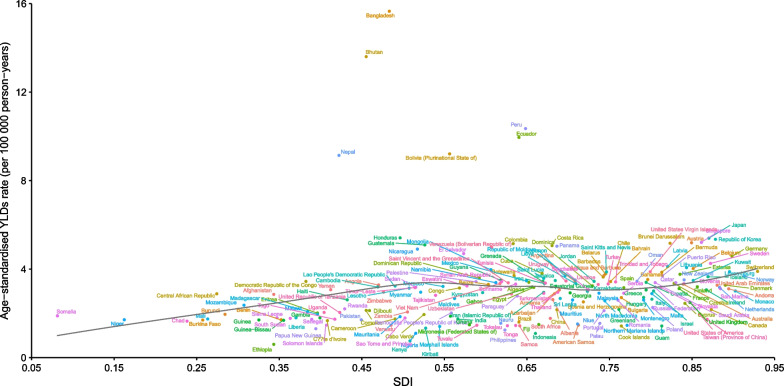
Age-standardized YLDs rates of appendicitis by 204 countries and territories and SDI, 2019. Expected values based on Socio-demographic Index and disease rates in all locations are shown as the black line. YLDs, years lived with disability. SDI, Sociodemographic Index

The national-level analysis revealed a generally positive association between the age-standardized YLD rates and SDI. The observed levels in Bangladesh, Bhutan, Peru, Nepal, Ecuador, and many other countries were much higher than the expected levels. However, in higher SDI countries and territories, such as Ethiopia, Guam, Kiribati, and many other countries, the observed levels were much lower than the expected levels based on the SDI (Fig. 5).

## Discussion

In the present study, we present the prevalence, incidence, and YLD and age-standardized rates for appendicitis in 204 countries and territories from 1990 to 2019. In 2019, there were 672,203 prevalent cases, 17,698,765 incident cases, and 211,113 YLD cases globally. The age-standardized prevalence, incidence, and YLDs rates were all increased from 1990 to 2019. To our best knowledge, the present study is the first to estimate the associations of age-standardized rates with the SDI in 21 GBD regions and 204 countries. The YLD rate of appendicitis increased with increasing SDI in terms of region and country.

Acute appendicitis is an acute condition, and it can lead to serious complications1,2, and some complications can lead to live in disability perpetually. Therefore, timely treatment can prevent serious complications. If hospital intraduct policies of prevent acute appendicitis, it can get faster treatment.

Appendicitis is one of the most common causes of abdominal pain in children and young adults, and occurs when the lumen of the vermiform appendix becomes inflamed, typically because of an obstruction. The condition can lead to death as well as significant costs to the healthcare system [27]. In the United States, the cost of hospitalization related to appendicitis may be as high as $3 billion a year [28]. The incidence of appendicitis is 7.8% [29] and a 2017 study showed it has increased in western countries in 1900 and declined until the middle of the 20th [4, 30]. However, the latest data show that the incidence of appendicitis has been on the rise and no complete and comprehensive study addressing these data has been published. Therefore, a comprehensive and comparable analysis of the burden of appendicitis must be performed to help decision makers and healthcare providers develop successful strategies to reduce the burden of appendicitis. In the present study, we report the prevalence, incidence, and YLDs of appendicitis in the general population in 204 countries and territories at the global, regional, and national levels in terms of the number and age-standardized rates stratified by age, sex, and SDI from 1990 to 2019.

GBD 2013 reported that the number of incident cases of appendicitis was 16 million in 2013 [31] and increased to 19 million in 2017 [32], whereas the number of incident cases was 17.7 million in 2019. The age-standardized incidence rate was 225.2 per 100,000 population in 2013 and 251.72 per 100,100 population in 2017, with a decrease of 14.58% from 1990 to 2013 and an increase of 1.8% from 1990 to 2017 [31, 32]. In GBD 2019, the age-standardized incidence rate increased from 190.7 per 100,000 population in 1990 to 229.9 per 100,000 population in 2019, and the age-standardized incidence rate showed an increase of 20.5%. Similar to the incidence, the age-standardized prevalence and YLDs rates also showed decreasing and increasing trends from 1990 to 2013 and from 1990 to 2017, respectively [31, 32]. Compared with GBD study, both the age-standardized prevalence and YLD rates showed an approximately tenfold increase from 1990 to 2019. This suggests that the burden of appendicitis increased over time, particularly from 2013 to 2019. However, number of incident cases in GBD 2019 was lower than that in GBD 2017. The results of these two studies could not be directly compared with our results, though, because of the different data sources and methodologies applied. For instance, GBD 2013 employed DisMod-MR 2.0, but GBD 2017 and GBD 2019 used DisMod-MR 2.1, to pool the available data. Moreover, compared with GBD 2017, GBD 2019 added subnational location data, such as Italy and Poland, and added estimates for the new locations (Monaco, San Marino, Cook Islands, and Saint Kitts and Nevis) [15].

Most appendicitis studies focused on only specific regions or countries. Few have comprehensively investigated appendicitis at the regional and national levels globally. Although a previous publication reviewed the evolution of the global incidence of appendicitis during the twentieth century, data from most countries are unavailable [9]. In the present study, we found that the highest age-standardized rates were more common in less-developed regions, such as Andean Latin America, Central Latin America, and Central Sub-Saharan Africa. This result was somewhat consistent with a previous review, which reported that the incidence in Asia, South America, and the Middle East was much higher than that in Western countries, and the incidence had increased in newly industrialized countries in the Middle East, South America, Asia, and Africa [4, 5, 9, 11, 12]. At the country level, the present study found less-developed and developing countries, namely Bangladesh, Bhutan, and Peru, had the highest age-standardized incidence, prevalence, and YLD rates, were further confirmed the above findings. The above results suggest that the burden of appendicitis in less-developed and developing countries, particularly in newly industrialized countries, is higher than that in developed countries. The differences between regions and countries may be due to differences in healthcare systems, socioeconomic statuses of the population, race, eating habits, and environmental exposures [4, 5, 9, 11, 12]. Therefore, prevention measures, management strategies and policies to reduce the burden of appendicitis should be given priority in these areas by policy makers.

Notably, the data in GBD 2019 were estimated using DisMod-MR 2.1 because only a few countries or territories provide actual population-based national data on the burden of appendicitis. Therefore, these national-level estimates should be interpreted with caution. If possible, additional attention should be given to health data collection to acquire representative data from every country. Additional resources are recommended to reduce the burden of appendicitis in low- and middle-income countries; thus, increased global cooperation might be necessary. These resources will contribute to more accurate predictions of the global burden of appendicitis and provide a basis for policy making.

As shown in previous studies [5, 12], although the burden was slightly higher in female individuals in our study, no statistically significant difference was found in the prevalence and incidence rates between male and female individuals. Hence, both female and male individuals should be given equal priority in prevention and treatment policies. However, some studies showed that the incidence of appendicitis was higher in male than in female individuals, likely because of geographic variations [4, 9, 12]. In 2019, the age-standardized prevalence, incidence, and YLD rates peaked in the 15- to 19-year age groups in both male and female individuals. These results were similar to those of a previous study that reported that the highest incidence was in the 15- to 19-year age group in female individuals and the 10- to 14-year age group in male individuals. Other studies confirmed this result and agreed that adolescents aged between 10 and 19 years had the highest burden of appendicitis [4, 9, 12, 33].

To our best knowledge, the associations of the burden of appendicitis with the development levels of regions and countries have not been compared in previous studies. The present study found that the SDI was an important factor in the appendicitis burden, and a generally positive association was observed between the regional- and national-level SDI and YLD because of appendicitis from 1990 to 2019. Thus, the burden of appendicitis was generally lower in countries with higher socioeconomic development levels; however, the burden of appendicitis was not limited to either more-developed or less-developed regions or countries, because low burdens of appendicitis were observed in regions and countries with different SDI. This phenomenon could be attributed to an early accurate diagnosis and effective interventions, such as appendectomy and antibiotics. Countries with high socioeconomic development levels have better diagnostic tools and treatment facilities than those with low socioeconomic development levels [11, 12]. The burdens of appendicitis were higher than the expected levels in some regions, such as Andean Latin America, Central Sub-Saharan Africa, Central Latin America, and High-income Asia Pacific, and other countries such as Andean Latin America, the Caribbean, and South Asia. When considering prevention policies, the observed burden should be combined with the expected burden based on the SDI in each region and country/territory.

Detecting and controlling risk factors are important approaches in prevention strategies. The risk factors for appendicitis include geographic and socioeconomic factors, race, seasonal patterns (the risk is highest in the summer), air pollution, dietary fiber, luminal obstruction, gastrointestinal infection, and genetic factors [9, 33–38]. High temperatures in the summer, an important risk factor, must be considered in the development of regional- and national-level prevention programs, as well as global warming. In GBD 2019, risk factors for appendicitis such low fruit consumption, low vegetable consumption, education level and LDI were also evaluated in appendicitis mortality estimation [15]. Thus, policymakers should consider those risk factors in their policy making.

Some limitations should be noted. First, the data included in the present study were secondary data from GBD 2019. The accuracy and robustness of GBD 2019 mainly depend on the quality and quantity of the input data used in the DisMod-MR 2.1 model. Second, the effects of different diagnosis, prevention strategies, and management policies in different regions and countries were not assessed, and substantial variations may exist, even in different regions and countries with the same SDI. Finally, because of the lack of relevant data, the burden of appendicitis stratified by histology was not evaluated in the present study.

## Conclusion

In summary, appendicitis remains a major public health challenge globally. Globally, the age-standardized prevalence, incidence, and YLD rates increased from 1990 to 2019. The highest burden of appendicitis was in adolescents, and no statistically significant difference was found between male and female individuals. Increasing awareness of appendicitis and its risk factors and the importance of early diagnosis and treatment are warranted to reduce the burden of appendicitis. Improving appendicitis health data in all regions and countries and monitoring the burden and treatment of appendicitis should be given more attention. Our study may be useful for policymakers to efficiently allocate resources to improve the diagnosis and treatment of appendicitis and reduce its modifiable risk factors.

## Supplementary Information

**Additional file 1.** The data sources, estimation and data table of appendicitis in 204 countries and territories, 1990–2019: a systematic analysis for the Global Burden of Disease Study 2019.

**Additional file 2.** The figures of appendicitis burden in 204 countries and territories, 1990–2019: a systematic analysis from the Global Burden of Disease Study 2019.

## Data Availability

The datasets generated for this study can be found in the GBD at http://ghdx.healthdata.org/gbd-results-tool. The data analyzed during this study could refer to Table 1 and Additional file 1: Tables S5–S7. In addition, data are available from the corresponding author on reasonable request.

## Abbreviations

YLD: Years lived with disability
GBD: The Global Burden of Diseases, Injuries, and Risk Factors Study
SDI: Sociodemographic index
UIs: Uncertainty intervals
WHO: World Health Organization
YLL: Years of life lost
DALYs: Disability-adjusted life years
IHME: The Institute of Health Metrics and Evaluation
GATHER: Guidelines for Accurate and Transparent Health Estimate Reporting
COD: Cause of death
CSMRs: Calculate cause-specific mortality rates
LDI: Lag-distributed income
HAQI: The Healthcare Access and Quality Index
DWs: Disability weights
