# Supplementary material for: The global, regional, and national burden of appendicitis in 204 countries and territories, 1990–2019: a systematic analysis from the Global Burden of Disease Study 2019
Source: BMC Gastroenterol. 2023 Feb 22;23:44. doi: 10.1186/s12876-023-02678-7 (PMC9945388; doi:10.1186/s12876-023-02678-7)
Supplement: Supplementary file 1 — Additional file 1. The data sources, estimation and data table of appendicitis in 204 countries and territories, 1990–2019: a systematic analysis for the Global Burden of Disease Study 2019. [file 12876_2023_2678_MOESM1_ESM.pdf]

# Additional file 1

## The data sources, estimation and data table of appendicitis in 204 countries and territories, 1990–2019: a systematic analysis for the Global Burden of Disease Study 2019

**Section 1. Data sources……………………………………………………………………………………………...3-5**

**1.1 Data adjustment ……………………………………………………………………………………………………3-4**

**1.2 Data processing…………………………………………………………………………………………...………...4-5**

**Section 2. DisMod-MR 2.1 estimation……………………………………………………………………………………5-8**

**2.1. DisMod-MR 2.1 analytical process……………………………………………………………………………….5-6**

**2.2. DisMod-MR 2.1 likelihood estimation……………………………………………………………...………....6-8**

**References……………………………………………………………………………………………………………….…………12**

**List of supplemental Figure and Table**

**Figure S1: GBD 2019 DisMod-MR 2.1 analytical cascade……………………………………………….……...….9-10**

**Figure S2: The flowcharts of estimation for appendicitis……………………………………..………………………...….10**

**Table S1: Data Inputs for appendicitis morbidity modelling by parameter……………………………….……………..……………10**

**Table S2: MR-BRT Crosswalk Adjustment Factors for Appendicitis………………………………………………………………….11**

**Table S3: Summary of covariates used in the appendicitis DisMod-MR meta-regression model……………………………..…………..11**

**Table S4: Covariates used in appendicitis mortality modelling…………………………………………………………………………..12**

**Table S5: Prevalent cases of appendicitis in 1990 and 2019 for both sexes and percentage change of age-standardised rates (ASR) by location……………………………………………………………………..………………………………………………......13-22**

**Table S6: Incident cases of appendicitis in 1990 and 2019 for both sexes and percentage change of age-standardised rates (ASR) by location………………………………………………………………………………………..…………………….……….23-34**

**Table S7: YLDs of appendicitis in 1990 and 2019 for both sexes and percentage change of age-standardised rates (ASR) by location…………………………………………………………………………………………………..…………………..35-44**

## Section 1. Data sources

### 1.1 Data adjustment

In order to make the data more consistent and suitable for modelling, IHME corrected the claims and hospital discharges data, used a number of other adjustments to extracted nonfatal sources. In the second step of nonfatal estimation, commonly applied adjustments included age-sex splitting, bias correction, adjustments for underreporting of notification data, and computing expected values of excess mortality. Age-sex splitting was commonly utilized to literature data reported by age or sex but not by age and sex. For GBD 2019, IHME split all data reported in age groups with a width greater than 20 years, using age patterns from available survey microdata or regional patterns derived from an initial run of main modelling tool, DisMod-MR 2.1. For most of the bias correction of data for variations in study attributes, the meta-regression component of DisMod-MR 2.1 was applied, such as case definitions and measurement method. DisMod-MR 2.1 calculates a single adjustment that is applied regardless of age, sex, or location. IHME applied bias corrections to the data before entry into DisMod-MR 2.1, if enough data were available to differentiate these adjustments by age, sex, or location, or if detailed survey data were available to make more precise adjustments between different thresholds on a biochemical measure. Age-specific correction factors were derived, due to the relationship varied with age. The correction of notification data for underreporting relied on studies that had examined the gap between true incidence and notified cases.

In GBD 2019, IHME estimated expected values of excess mortality from prevalence or incidence and cause-specific mortality rate (CSMR) data for every cause for which deaths were estimated apart from deaths due to maternal causes and deaths due to non-maternal causes for women of reproductive age, live births and all-cause mortality estimates can be used to calculate deaths. IHME matched every prevalence data point (or incidence data for short-duration conditions) with the CSMR value corresponding to the age range, sex, year, and location of the data point. IHME restricted this to data points reporting age-groups spanning 20 years or less. The ratio of CSMR to prevalence (or incidence times a short duration) is conceptually equivalent to an excess mortality rate. To reflect a gradient in excess mortality, we added in all relevant models the log of lag distributed income (LDI) or the Healthcare Access and Quality (HAQ) index as a covariate, with a strong prior that as LDI or HAQ Index increases, excess mortality declines [1].

**1.2 Data processing**

Hospital discharge data provide observations about encounters, generally with only the primary diagnostic code for the encounter. Claims data, on the other hand, link claims for all inpatient and outpatient encounters for a single individual, and provide primary and secondary diagnoses for all encounters. In GBD 2017, if an individual has one or more inpatients who have encountered the appropriate ICD code for any diagnosis, the individual is extracted from the claim data as an event case. Readmission within 28 days was considered to be due to the same onset of illness.

GBD 2019 improved data processing methods to capture cases that were diagnosed and/or treated in an outpatient setting. Specifically, incident cases were extracted from claim data if an individual had at least one inpatient or outpatient encounter with an appropriate ICD code as any diagnosis within one year. Data from hospital discharges were, then, adjusted using correction factors from claims, converting encounters to estimates of cases, accounting for most locations providing only primary diagnostic codes, and estimating outpatient cases from inpatient cases. The USA claims data from the year 2000 and from the years 2010 2016 were each adjusted to data from hospital discharges outside DisMod using MR-BRT analysis to adjust for selection bias due to commercial insurance. The table S2 below shows bias correction factors estimated using MR-BRT. Data points with an age-standardised incidence rate greater than three median absolute deviations from the median of the age-standardised incidence rate for all inpatient and non-USA claims data were marked as outliers and excluded from analysis [1].

## Section 2. DisMod-MR 2.1 estimation

### 2.1. DisMod-MR 2.1 analytical process

Figure S1 shows the DisMod-MR 2.1 analytical process. IHME divided the sequence of estimation occurs into five levels: global, super-region, region, country and, where applicable, subnational location. The super-region priors are generated at the global level with mixed-effects, nonlinear

regression using all available data; the super-region fit, in turn, informs the region fit, and so on down the cascade. The wrapper gives analysts the choice to branch the cascade in terms of time and sex at different levels depending on data density. The default used in most models is to branch by sex after the global fit but to retain all years of data until the lowest level in the cascade.

**2.2. DisMod-MR 2.1 likelihood estimation**

The Gaussian, log-Gaussian, Laplace or Log-Laplace likelihood function in DisMod-MR 2.1 was used to analyzed. The default log-Gaussian equation for the data likelihood is:

$$-\log\left[p\left(y_j|\Phi\right)\right] = \log\left(\sqrt{2\pi}\right) + \log\left(\delta_j + s_j\right) + \frac{1}{2\left(\frac{\log\left(a_j+\eta_j\right)-\log\left(m_j+\eta_j\right)}{\delta_j + s_j}\right)^2}$$

where, y j is a 'measurement value' (i.e., data point); Φ denotes all model random variables; $\theta\ k$ is the offset value, eta, for particular 'integrand' (prevalence, incidence, remission, excess mortality rate, with-condition mortality rate, cause-specific mortality rate, relative risk or standardized mortality ratio) and $b\ k$ is the adjusted measurement for data point j, defined by:

$$a_j = e^{(-u_j - c_j)} y_j$$

where u j is the total ‘area effect’ (i.e., the sum of the random effects at three levels of the cascade: super-region, region and country) and c j is the total covariate effect (i.e., the mean combined fixed effects for sex, study covariates, and country level covariates), defined by:

$$c_j = \sum_{k=0}^{K[I(j)]-1} \beta_{I(j),k} X_{k,j}$$

with standard deviation

$$s_j = \sum_{l=0}^{L[I(j)]-1} \zeta_{I(j),l} Z_{k,j}$$

where k denotes the mean value of each data point in relation to a covariate (also called x-covariate); I(j) denotes a data point for a particular integrand, j; $\beta_{I(j),k}$ is the multiplier of the k$^{th}$ x-covariate for the i$^{th}$ integrand; $X_{k,j}$ is the covariate value corresponding to the data point j for covariate k; l denotes the standard deviation of each data point in relation to a covariate (also called z-covariate); $\zeta_{I(j),l}$ is the multiplier of the I$^{th}$ z-covariate for the i$^{th}$ integrand; and $\delta_j$ is the standard deviation for adjusted measurement j, defined by:

$$\delta_j = \log\left[y_j + e^{(-u_j-c_j)}\eta_j + c_j\right] - \log\left[y_j + e^{(-u_j-c_j)}\eta_j\right]$$

Where $m_j$ denotes the model for the j$^{th}$ measurement, not counting effects or measurement noise and defined by:

$$m_j = \frac{1}{B(j) - A(j)} \int_{A(j)}^{B(j)} I_j(a)da$$

where A(j) is the lower bound of the age range for a data point; B(j) is the upper bound of the age range for a data point; and Ij denotes the function of age corresponding to the integrand for data point j [1].

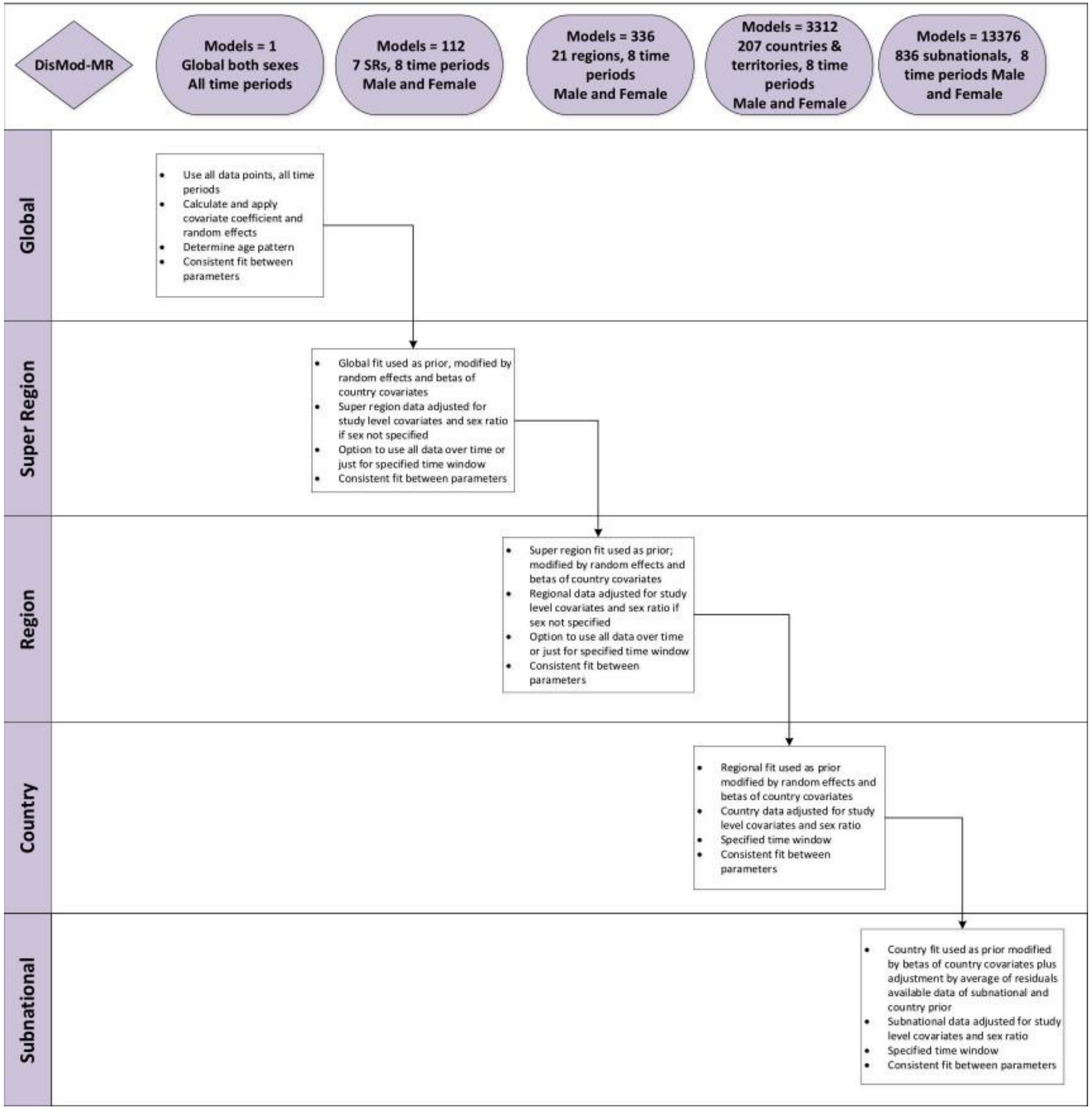
DisMod-MR
Models = 1
Global both sexes
All time periods
Models = 112
7 SRs, 8 time periods
Male and Female
Models = 336
21 regions, 8 time periods
Male and Female
Models = 3312
207 countries & territories, 8 time periods
Male and Female
Models = 13376
836 subnationals, 8 time periods Male and Female
Global
• Use all data points, all time periods
• Calculate and apply covariate coefficient and random effects
• Determine age pattern
• Consistent fit between parameters
Super Region
• Global fit used as prior, modified by random effects and betas of country covariates
• Super region data adjusted for study level covariates and sex ratio if sex not specified
• Option to use all data over time or just for specified time window
• Consistent fit between parameters
Region
• Super region fit used as prior; modified by random effects and betas of country covariates
• Regional data adjusted for study level covariates and sex ratio if sex not specified
• Option to use all data over time or just for specified time window
• Consistent fit between parameters
Country
• Regional fit used as prior modified by random effects and betas of country covariates
• Country data adjusted for study level covariates and sex ratio
• Specified time window
• Consistent fit between parameters
Subnational
• Country fit used as prior modified by betas of country covariates plus adjustment by average of residuals available data of subnational and country prior
• Subnational data adjusted for study level covariates and sex ratio
• Specified time window
• Consistent fit between parameters

**Figure S1: GBD 2019 DisMod-MR 2.1 analytical cascade[1].**

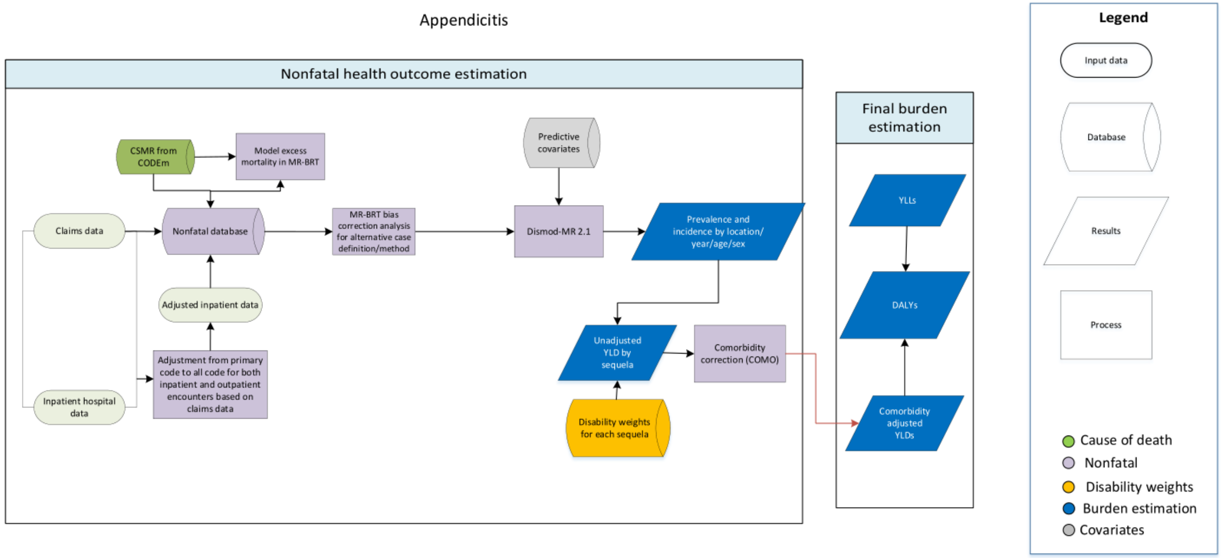


**Figure S2: The flowcharts of estimation for appendicitis [1].**

**Table S1. Data Inputs for appendicitis morbidity modelling by parameter [1].**

| Measure | Total sources | Countries with data |
| --- | --- | --- |
| Incidence | 297 | 46 |

### Table S2. MR-BRT Crosswalk Adjustment Factors for Appendicitis[1].

| Data input | Reference or alternative data collection | Gamma | Beta Coefficient, Log (95% CI) | Adjustment factor* |
|---|---|---|---|---|
| Hospital + non-USA claims | Ref | 0.06 | --- | --- |
| USA claims from year 2000 | Alt | | -0.57 (-0.30, -0.85) | 0.56 (0.43, 0.74) |
| USA claims from year 2010-2016 | Alt | | -0.06 (-0.20, 0.08) | 0.94 (0.82, 1.09) |

*Adjustment factor is the transformed Beta coefficient in normal space, and can be interpreted as the factor by which the alternative case definition is adjusted to reflect what it would have been if measured as the reference.

### Table S3: Summary of covariates used in the appendicitis DisMod-MR meta-regression model [1].

| Covariate | Type | Parameter | Exponentiated beta (95% Uncertainty Interval) |
|---|---|---|---|
| Fibre unadjusted (g) | Country-level | Incidence | 1.00 (0.99, 1.00) |
| Healthcare access and quality index | Country-level | Excess mortality rate | 0.94 (0.94, 0.94) |

**Table S4. Covariates used in appendicitis mortality modelling [1].**

| Level | Covariate | Direction |
|---|---|---|
| 2 | Age-sex-specific scaled exposure variable for low fruit consumption | + |
| | Age-sex-specific scaled exposure variable for low vegetable consumption | + |
| | Healthcare Access and Quality Index | - |
| 3 | Socio-demographic Index | - |
| | Education (years per capita) | - |
| | Log LDI ($I per capita) | - |

## Reference:

1. **Global burden of 369 diseases and injuries in 204 countries and territories, 1990-2019: a systematic analysis for the Global Burden of Disease Study 2019**. *Lancet (London, England)* 2020, **396**(10258):1204-1222.

| Table S5: Prevalent cases of appendicitis in 1990 and 2019 for both sexes and percentage change of age-standardised rates(ASR) by location | | | | | |
|---|---|---|---|---|---|
| | 1990 | | 2019 | | Percentage change in ASR from 1990 and 2019 |
| | Counts (95% UI) | Rate (95% UI) | Counts (95% UI) | Rate (95% UI) | |
| Global | 409125(318852 to 520824) | 7.2(5.7 to 9.1) | 672203(536225 to 847977) | 8.7(6.9 to 11) | 20.8(18.9 to 23) |
| High-income North America | 19400(15577 to 24258) | 6.9(5.5 to 8.7) | 21877(19671 to 24295) | 6.2(5.5 to 6.9) | -10.4(-21.8 to 4) |
| Canada | 1878(1455 to 2408) | 6.7(5.1 to 8.7) | 2412(1910 to 2983) | 6.8(5.2 to 8.6) | 0.8(-3.6 to 4.9) |
| Greenland | 4(3 to 5) | 6.3(4.8 to 8.2) | 4(3 to 5) | 6.8(5.3 to 8.7) | 9.1(3.2 to 14.8) |
| United States of America | 17518(14125 to 21827) | 6.9(5.5 to 8.7) | 19461(17635 to 21474) | 6.1(5.5 to 6.8) | -11.6(-24.1 to 4.3) |
| Australasia | 2173(1665 to 2899) | 10.6(8.1 to 14.1) | 2850(2247 to 3629) | 10.8(8.4 to 14) | 2.4(-2.1 to 6.4) |
| Australia | 1772(1348 to 2385) | 10.4(7.8 to 13.9) | 2395(1859 to 3102) | 10.7(8.2 to 14.1) | 3.6(-1.2 to 7.9) |
| New Zealand | 402(314 to 520) | 11.6(9 to 14.8) | 455(388 to 539) | 11.4(9.6 to 13.7) | -1.9(-12.3 to 9.4) |
| High-income Asia Pacific | 29564(21988 to 39011) | 17.6(13.1 to 23.3) | 22677(17978 to 28337) | 17.2(13.1 to 22.1) | -2.6(-7.2 to 2.3) |
| Brunei Darussalam | 46(34 to 61) | 15.5(11.5 to 20.4) | 74(56 to 96) | 16.6(12.3 to 21.7) | 7.1(1.8 to 13.2) |
| Japan | 21116(15835 to 27687) | 18.4(13.7 to 24.2) | 14750(11843 to 18238) | 17.3(13.4 to 21.9) | -6.2(-12.2 to 2.2) |
| Singapore | 529(398 to 700) | 16.3(12.2 to 21.3) | 750(579 to 958) | 16.7(12.4 to 22) | 2(-3.4 to 7.1) |
| Republic of Korea | 7873(5798 to 10502) | 16(11.9 to 21) | 7103(5457 to 9049) | 17(12.6 to 22.4) | 6.4(0 to 12.3) |
| Western Europe | 34930(27014 to 45439) | 9.5(7.3 to 12.4) | 38356(31229 to 47142) | 10.6(8.5 to 13.6) | 12.1(6.6 to 18.3) |
| Andorra | 6(4 to 7) | 9.6(7.2 to 12.8) | 7(6 to 9) | 10(7.5 to 13.3) | 4.3(-0.3 to 9.2) |
| Austria | 1008(858 to 1171) | 14.6(12.3 to 17.1) | 1153(1067 to 1248) | 16.7(15.3 to 18.2) | 14.6(2.1 to 29.4) |
| Belgium | 1008(778 to 1326) | 10.7(8.2 to 14.3) | 1235(1034 to 1463) | 12.9(10.6 to 15.6) | 20.3(4.9 to 37.2) |
| Cyprus | 57(42 to 77) | 8.2(6.2 to 10.7) | 880(719 to 1050) | 10.7(8.3 to 13.1) | 29.9(12.6 to 52.1) |
| Denmark | 496(384 to 646) | 9.9(7.6 to 13.1) | 541(417 to 696) | 10.5(7.9 to 13.9) | 5.6(1.2 to 9.9) |

| | | | | | |
|---|---|---|---|---|---|
| Finland | 521(441 to 617) | 10.9(9.2 to 13.1) | 498(429 to 582) | 10.3(8.7 to 12.3) | -5.7(-10.7 to -0.6) |
| France | 5278(4011 to 6980) | 9.5(7.2 to 12.6) | 5654(4374 to 7300) | 10(7.6 to 13.2) | 5.6(0.8 to 10.2) |
| Germany | 8419(6473 to 11013) | 11.3(8.5 to 15) | 9985(8081 to 12091) | 14.8(11.6 to 18.4) | 30.6(16 to 46.5) |
| Greece | 923(693 to 1231) | 9.3(6.9 to 12.4) | 809(623 to 1036) | 9.8(7.4 to 13.2) | 5.6(0.8 to 10.6) |
| Iceland | 27(20 to 35) | 10.4(7.9 to 13.6) | 37(29 to 48) | 11.8(9 to 15.4) | 13.4(6.7 to 20.8) |
| Ireland | 344(259 to 468) | 9.4(7.1 to 12.7) | 437(334 to 566) | 9.9(7.4 to 13.2) | 5.4(0.3 to 9.8) |
| Israel | 460(345 to 615) | 9.1(6.8 to 12) | 852(644 to 1136) | 9.7(7.3 to 13) | 6.6(1.3 to 11.5) |
| Italy | 4356(3281 to 5758) | 7.8(5.9 to 10.4) | 3594(2974 to 4406) | 8.4(6.7 to 10.6) | 7.6(-4.1 to 23.9) |
| Luxembourg | 44(35 to 55) | 12.6(9.9 to 16.1) | 66(57 to 75) | 12.4(10.5 to 14.3) | -1.9(-14 to 14.2) |
| Malta | 29(22 to 38) | 8(5.9 to 10.6) | 33(27 to 40) | 9.3(7.5 to 11.4) | 17(3.6 to 34.9) |
| Monaco | 2(2 to 3) | 9.3(7 to 12.5) | 3(2 to 4) | 9.7(7.3 to 12.9) | 3.9(-0.1 to 8.9) |
| Netherlands | 1459(1123 to 1925) | 9.8(7.4 to 13.1) | 1502(1170 to 1955) | 10.1(7.7 to 13.4) | 2.9(-1.7 to 8.2) |
| Norway | 441(339 to 582) | 10.8(8.2 to 14.2) | 568(447 to 722) | 11.8(9.1 to 15.2) | 9.6(5.4 to 14) |
| San Marino | 2(2 to 3) | 9.5(7.1 to 12.5) | 3(2 to 4) | 10(7.5 to 13.4) | 4.9(0.1 to 9.2) |
| Portugal | 571(421 to 763) | 5.7(4.2 to 7.6) | 569(467 to 701) | 6.8(5.4 to 8.6) | 19.4(6.2 to 35.4) |
| Spain | 3661(2769 to 4831) | 9.4(7.1 to 12.5) | 3839(2976 to 4866) | 10.1(7.6 to 13.3) | 7.2(2.7 to 11.8) |
| Sweden | 967(744 to 1249) | 12.5(9.4 to 16.4) | 1187(925 to 1523) | 13.8(10.3 to 18.1) | 10.4(5.8 to 15.7) |
| Switzerland | 786(660 to 925) | 12.2(10.2 to 14.5) | 934(810 to 1073) | 12.5(10.6 to 14.6) | 2.3(-1.7 to 7) |
| United Kingdom | 4039(3185 to 5195) | 7.6(5.8 to 9.8) | 4730(3918 to 5828) | 8(6.4 to 10) | 5.8(0.8 to 11.5) |
| Southern Latin America | 3914(2994 to 5125) | 7.8(6 to 10.1) | 7736(6046 to 9784) | 11.7(9 to 14.9) | 49.8(42 to 57.6) |
| Argentina | 2538(1948 to 3295) | 7.7(5.9 to 9.9) | 5020(3872 to 6520) | 11.1(8.5 to 14.5) | 45.2(36.5 to 54.7) |
| Chile | 1132(862 to 1499) | 8.1(6.2 to 10.5) | 2346(1896 to 2872) | 13.2(10.5 to 16.2) | 63.3(50.3 to 78.9) |
| Uruguay | 244(189 to 319) | 7.9(6.1 to 10.4) | 370(284 to 471) | 11.2(8.5 to 14.5) | 40.8(33.5 to 48.3) |
| Eastern Europe | 18490(14050 to 24211) | 8.7(6.6 to 11.4) | 18435(14229 to 23497) | 10.4(7.8 to 13.6) | 19.9(17.3 to 22.8) |
| Belarus | 1186(920 to 1547) | 11.8(9.1 to 15.5) | 991(772 to 1242) | 12.1(9.2 to 15.8) | 2.4(-2.4 to 8.4) |

| | | | | | |
|---|---|---|---|---|---|
| Estonia | 176(135 to 225) | 11.8(9 to 15.3) | 133(103 to 169) | 12.1(9.2 to 16) | 1.9(-3.3 to 7.1) |
| Latvia | 311(243 to 395) | 12.3(9.5 to 16.1) | 203(165 to 248) | 13.2(10.3 to 16.6) | 6.6(-1 to 15.1) |
| Lithuania | 444(347 to 570) | 12.4(9.6 to 15.9) | 304(244 to 369) | 13.1(10.2 to 16.4) | 5.4(-0.1 to 11.7) |
| Republic of Moldova | 528(406 to 684) | 12(9.2 to 15.6) | 387(303 to 496) | 11.8(9 to 15.5) | -1.7(-6.6 to 3.7) |
| Russian Federation | 11097(8339 to 14504) | 7.8(5.8 to 10.3) | 12538(9621 to 16028) | 10.2(7.6 to 13.3) | 29.9(26.7 to 34.4) |
| Ukraine | 4749(3634 to 6139) | 9.8(7.4 to 12.8) | 3878(2998 to 4932) | 10.5(8 to 13.7) | 7.8(2.1 to 14.1) |
| Central Europe | 9298(7043 to 12032) | 7.8(5.9 to 10.2) | 7814(6451 to 9388) | 8.4(6.7 to 10.4) | 7.6(0.6 to 15.8) |
| Albania | 284(210 to 382) | 7.8(5.9 to 10.3) | 203(151 to 262) | 8.3(6.1 to 10.8) | 5.9(-0.5 to 12.4) |
| Bosnia and Herzegovina | 365(275 to 479) | 7.9(5.9 to 10.4) | 221(168 to 280) | 7.9(5.8 to 10.5) | 0.8(-4.4 to 6.5) |
| Bulgaria | 664(504 to 859) | 8.3(6.2 to 10.9) | 481(377 to 595) | 8.6(6.4 to 11.2) | 3.9(-1 to 9) |
| Croatia | 484(412 to 559) | 9.4(7.3 to 12) | 1234(972 to 1557) | 12.2(9.3 to 15.7) | 30.3(22.9 to 37.2) |
| Czechia | 818(622 to 1064) | 4.1(3.1 to 5.3) | 1514(1094 to 2036) | 5.2(3.9 to 7) | 28.8(21.1 to 39) |
| Hungary | 817(630 to 1052) | 8.3(6.2 to 10.8) | 689(537 to 876) | 8.6(6.4 to 11.4) | 3.7(-2.2 to 9.1) |
| North Macedonia | 159(116 to 212) | 7.8(5.6 to 10.3) | 153(115 to 198) | 8.1(6 to 10.7) | 4.7(-1 to 10.3) |
| Montenegro | 51(38 to 67) | 8(5.9 to 10.6) | 45(34 to 58) | 8.2(6 to 10.7) | 2(-2.6 to 8.6) |
| Poland | 2550(1887 to 3382) | 7(5.1 to 9.3) | 2206(1893 to 2584) | 7.1(5.9 to 8.5) | 2.5(-11.1 to 20.3) |
| Romania | 1839(1387 to 2390) | 8(6 to 10.5) | 1296(999 to 1646) | 8.2(6.1 to 10.8) | 2.7(-2.2 to 8.3) |
| Serbia | 661(504 to 858) | 7.4(5.5 to 9.6) | 671(547 to 805) | 9.1(7.2 to 11.3) | 24.5(11.6 to 40.2) |
| Slovakia | 438(324 to 578) | 8.4(6.2 to 11) | 443(352 to 550) | 10(7.7 to 12.8) | 19.2(9 to 29) |
| Slovenia | 167(125 to 217) | 8.8(6.5 to 11.5) | 168(150 to 188) | 10.6(9.2 to 12.1) | 20.2(-1.6 to 50.7) |
| Central Asia | 6897(5161 to 9046) | 9.2(7 to 11.9) | 8907(6690 to 11734) | 9.4(7 to 12.3) | 1.5(-2 to 5.4) |
| Armenia | 307(228 to 404) | 8.7(6.5 to 11.5) | 261(200 to 339) | 9.7(7.2 to 12.8) | 11.2(5.7 to 17) |
| Azerbaijan | 709(529 to 943) | 8.8(6.7 to 11.6) | 929(699 to 1240) | 9.2(6.8 to 12.4) | 3.8(-2.4 to 10.8) |
| Georgia | 443(331 to 577) | 8.2(6.2 to 10.8) | 288(217 to 371) | 9.3(6.8 to 12.3) | 13(7.3 to 19.8) |
| Kazakhstan | 1630(1221 to 2109) | 9.5(7.2 to 12.3) | 1679(1279 to 2193) | 9.5(7.1 to 12.5) | 0(-6.3 to 6.7) |

| | | | | | |
|---|---|---|---|---|---|
| Kyrgyzstan | 403(293 to 540) | 8.4(6.3 to 11) | 632(468 to 841) | 9.3(6.9 to 12.4) | 10.6(4.7 to 16.5) |
| Mongolia | 550(444 to 664) | 21.1(17.4 to 25.3) | 421(338 to 526) | 12.7(10.1 to 16) | -39.7(-46.1 to -30.6) |
| Tajikistan | 511(371 to 683) | 8.6(6.5 to 11.2) | 928(686 to 1228) | 8.9(6.7 to 11.8) | 4(-1.4 to 9.4) |
| Turkmenistan | 356(262 to 470) | 8.6(6.5 to 11.2) | 481(364 to 632) | 9.3(7 to 12.2) | 7.9(2.1 to 13.4) |
| Uzbekistan | 1988(1434 to 2663) | 8.6(6.4 to 11.3) | 3288(2454 to 4374) | 9.2(6.9 to 12.1) | 6.9(1.3 to 13.3) |
| Central Latin America | 22753(17070 to 29064) | 11.7(9.1 to 14.8) | 34366(26234 to 44412) | 13.6(10.3 to 17.6) | 16.1(12.3 to 20.1) |
| Colombia | 5086(3716 to 6648) | 14(10.5 to 18) | 7640(5835 to 9809) | 16.5(12.5 to 21.2) | 18.4(13 to 24.4) |
| Costa Rica | 521(381 to 705) | 15.6(11.8 to 20.5) | 733(555 to 948) | 16.1(12 to 21.1) | 3.3(-2.8 to 9.9) |
| El Salvador | 1208(959 to 1525) | 18.9(15.4 to 23.4) | 958(717 to 1249) | 14.9(11.1 to 19.5) | -21.3(-31 to -11.5) |
| Guatemala | 2265(1898 to 2688) | 24.5(20.9 to 28.6) | 3264(2546 to 4181) | 16.3(12.9 to 20.4) | -33.6(-42.8 to -22.8) |
| Honduras | 961(763 to 1205) | 17.5(14.4 to 21.6) | 1905(1473 to 2421) | 17.5(13.8 to 21.8) | -0.2(-9.3 to 10.6) |
| Mexico | 8310(5965 to 11185) | 7.8(5.7 to 10.3) | 13717(10193 to 17852) | 10.9(8.1 to 14.3) | 39.8(36 to 44.2) |
| Nicaragua | 650(480 to 850) | 14.1(10.9 to 17.8) | 1107(827 to 1449) | 15.8(11.9 to 20.4) | 12(4.5 to 20.9) |
| Panama | 437(324 to 571) | 16.2(12.4 to 20.9) | 672(503 to 885) | 16.1(12 to 21.3) | -0.8(-6.7 to 6) |
| Venezuela (Bolivarian Republic of) | 3314(2494 to 4254) | 15.5(12 to 19.6) | 4368(3313 to 5655) | 15.9(12 to 20.8) | 2.4(-3.6 to 10.4) |
| Andean Latin America | 20735(17479 to 24588) | 46.9(40.3 to 55.1) | 21347(17402 to 26573) | 32.5(26.6 to 40.2) | -30.7(-36.6 to -22.2) |
| Bolivia (Plurinational State of) | 2466(2065 to 2954) | 33.7(28.8 to 39.8) | 3745(3008 to 4739) | 30(24.3 to 37.6) | -11.2(-19 to -0.1) |
| Ecuador | 2958(2467 to 3592) | 26.4(22.4 to 31.6) | 5955(5139 to 7008) | 32.4(28.1 to 37.9) | 22.4(14 to 33.1) |
| Peru | 15311(12872 to 18119) | 60.3(51.6 to 70.8) | 11647(9147 to 15007) | 33.6(26.4 to 43.2) | -44.3(-51.5 to -33.8) |
| Caribbean | 3210(2481 to 4146) | 8.4(6.6 to 10.7) | 5046(3913 to 6473) | 10.8(8.3 to 13.9) | 28.8(25.3 to 32) |
| Antigua and Barbuda | 6(4 to 8) | 8.7(6.6 to 11.4) | 10(8 to 13) | 11.8(9 to 15.4) | 35.2(27.8 to 42.6) |
| Bahamas | 24(18 to 32) | 8.3(6.3 to 10.8) | 43(33 to 55) | 11.3(8.6 to 14.6) | 36(29.8 to 42.1) |
| Barbados | 23(17 to 30) | 8.6(6.5 to 11.2) | 31(24 to 39) | 11.7(8.9 to 15.3) | 36.6(29.8 to 43.9) |

| | | | | | |
|---|---|---|---|---|---|
| Belize | 16(12 to 22) | 7.7(5.9 to 10.1) | 49(36 to 64) | 10.6(8 to 13.8) | 36.9(30.4 to 44.7) |
| Bermuda | 5(4 to 7) | 8.7(6.6 to 11.2) | 6(5 to 8) | 12.4(9.3 to 16.3) | 42.8(35.8 to 49.9) |
| Cuba | 1093(842 to 1429) | 7.2(5.3 to 9.8) | 89(74 to 110) | 7.8(6.3 to 9.7) | 8.2(-3.9 to 24.6) |
| Dominica | 7(5 to 9) | 8.3(6.3 to 10.9) | 7(5 to 9) | 10.4(7.9 to 13.4) | 26.5(19.9 to 32.5) |
| Dominican Republic | 711(542 to 923) | 8.5(6.6 to 10.8) | 1313(1004 to 1719) | 11.6(8.9 to 15) | 36.4(27.8 to 44.7) |
| Grenada | 7(5 to 10) | 7.8(5.9 to 10.2) | 11(9 to 15) | 11(8.4 to 14.4) | 41.2(35 to 49) |
| Guyana | 74(58 to 94) | 8.4(6.7 to 10.5) | 93(74 to 120) | 11.3(9 to 14.4) | 34.5(27.4 to 42) |
| Haiti | 416(331 to 516) | 6(4.9 to 7.4) | 1138(895 to 1456) | 8.3(6.6 to 10.5) | 38.4(28.6 to 48.7) |
| Jamaica | 224(166 to 299) | 8.5(6.5 to 11.1) | 328(248 to 425) | 11.2(8.5 to 14.5) | 31.9(25.6 to 38.5) |
| Puerto Rico | 323(242 to 431) | 8.7(6.5 to 11.6) | 360(276 to 468) | 11.9(8.9 to 15.7) | 37(30.5 to 43.8) |
| Saint Kitts and Nevis | 4(3 to 5) | 9.3(7.3 to 12.2) | 7(6 to 9) | 12.4(9.5 to 16.1) | 32.8(25.3 to 40.2) |
| Saint Lucia | 12(9 to 17) | 8.1(6.2 to 10.6) | 19(15 to 25) | 11.4(8.8 to 15) | 41.4(34.7 to 49.4) |
| Saint Vincent and the Grenadines | 10(7 to 13) | 8(6.1 to 10.5) | 12(9 to 15) | 10.7(8.2 to 13.9) | 33.9(28.1 to 40.5) |
| Suriname | 33(25 to 43) | 7.7(5.9 to 9.9) | 61(46 to 79) | 10.6(8 to 13.8) | 38.5(31 to 45.7) |
| Trinidad and Tobago | 104(77 to 138) | 8(6 to 10.4) | 141(109 to 184) | 11(8.3 to 14.5) | 38.2(31.7 to 46) |
| United States Virgin Islands | 11(8 to 14) | 9.7(7.6 to 12.5) | 11(9 to 14) | 12.5(9.7 to 16.2) | 27.8(21.7 to 34.3) |
| Tropical Latin America | 6758(5191 to 8625) | 4(3.1 to 5) | 11326(9235 to 14000) | 5.1(4.1 to 6.3) | 28.9(23.6 to 35.5) |
| Brazil | 6250(4799 to 8002) | 3.8(2.9 to 4.8) | 10682(8725 to 13165) | 5(4 to 6.2) | 33.6(27.3 to 41.1) |
| Paraguay | 508(392 to 650) | 12.8(10.4 to 15.7) | 644(510 to 828) | 9(7.2 to 11.3) | -29.6(-33 to -25.7) |
| East Asia | 82768(62231 to 108086) | 6.2(4.8 to 8) | 98212(77893 to 120704) | 6.7(5.3 to 8.3) | 8.4(2.7 to 14.7) |
| China | 80491(60502 to 105413) | 6.2(4.8 to 8) | 95204(75486 to 117012) | 6.7(5.3 to 8.4) | 8.6(2.7 to 15) |
| Democratic People's Republic of Korea | 1335(1041 to 1689) | 6.5(5 to 8) | 1682(1321 to 2096) | 6.2(4.8 to 7.9) | -3.6(-8.8 to 1.9) |

| | | | | | |
|---|---|---|---|---|---|
| Taiwan (Province of China) | 942(721 to 1208) | 4.5(3.5 to 5.7) | 1326(1124 to 1550) | 5.3(4.5 to 6.4) | 17.6(7.6 to 31.3) |
| Southeast Asia | 24060(18239 to 31680) | 4.7(3.6 to 6) | 43365(33776 to 56231) | 6.3(4.9 to 8.1) | 34.2(30.6 to 37.7) |
| Cambodia | 964(745 to 1224) | 8.7(6.9 to 10.9) | 1762(1355 to 2310) | 10.1(7.9 to 13.2) | 16.3(8.5 to 24.4) |
| Indonesia | 3629(2613 to 4884) | 1.7(1.3 to 2.3) | 9114(6762 to 12161) | 3.4(2.5 to 4.5) | 97.8(92.1 to 105.2) |
| Lao People's Democratic Republic | 417(328 to 527) | 9.1(7.4 to 11.2) | 798(613 to 1040) | 10.3(8 to 13.2) | 12.4(3.9 to 22.7) |
| Malaysia | 1321(1003 to 1737) | 7.1(5.5 to 9.2) | 3032(2337 to 3955) | 9.3(7.1 to 11.9) | 29.9(23.5 to 37.2) |
| Maldives | 14(10 to 19) | 6(4.5 to 8) | 44(33 to 57) | 8.4(6.3 to 10.9) | 39.4(32.6 to 47.8) |
| Mauritius | 76(57 to 103) | 6.3(4.8 to 8.4) | 104(80 to 134) | 8.3(6.3 to 10.8) | 30.6(24.1 to 38.9) |
| Myanmar | 3760(2942 to 4780) | 8.3(6.6 to 10.4) | 5300(4089 to 6867) | 9.3(7.2 to 12) | 12.4(4.4 to 19.6) |
| Philippines | 1562(1141 to 2113) | 2.2(1.6 to 2.9) | 4705(3475 to 6357) | 3.9(2.9 to 5.2) | 79.7(75.7 to 83.9) |
| Sri Lanka | 1181(872 to 1570) | 6.3(4.8 to 8.3) | 1832(1395 to 2363) | 8.4(6.4 to 11) | 32.5(25.7 to 39.6) |
| Seychelles | 6(4 to 7) | 7.5(5.8 to 9.5) | 10(8 to 12) | 9.8(7.6 to 12.4) | 30.9(23.2 to 40.6) |
| Thailand | 5556(4231 to 7233) | 8.7(6.8 to 11.1) | 6274(4912 to 7968) | 9.4(7.2 to 12.2) | 8.4(1.8 to 14.5) |
| Timor-Leste | 65(50 to 83) | 7.8(6.2 to 9.9) | 147(112 to 195) | 10(7.9 to 12.8) | 27.2(20 to 34.9) |
| Viet Nam | 5480(4185 to 7217) | 7.6(5.9 to 9.7) | 10186(8126 to 12756) | 10.6(8.3 to 13.4) | 40.4(31.5 to 51.7) |
| Oceania | 242(183 to 317) | 3.6(2.8 to 4.6) | 577(444 to 755) | 4.1(3.2 to 5.4) | 16.2(12.2 to 21.1) |
| American Samoa | 2(1 to 3) | 3.9(3 to 5) | 3(2 to 3) | 4.5(3.5 to 5.8) | 15.9(9.9 to 22.6) |
| Cook Islands | 1(1 to 1) | 3.8(2.8 to 4.9) | 1(1 to 1) | 4.4(3.4 to 5.7) | 17.4(10.6 to 24.9) |
| Micronesia (Federated States of) | 4(3 to 5) | 3.9(3.1 to 4.9) | 5(4 to 6) | 4.6(3.6 to 5.8) | 16.6(10 to 23.2) |
| Fiji | 32(24 to 41) | 3.9(3 to 5) | 43(33 to 55) | 4.6(3.6 to 5.9) | 19.1(12.5 to 25.5) |
| Guam | 6(4 to 7) | 3.8(2.9 to 4.8) | 7(6 to 10) | 4.4(3.4 to 5.7) | 17.1(10.4 to 23.8) |
| Kiribati | 3(2 to 4) | 4(3.2 to 5) | 5(4 to 7) | 4.1(3.2 to 5.2) | 3.4(-2 to 8.7) |

| | | | | | |
|---|---|---|---|---|---|
| Nauru | 0(0 to 1) | 4.1(3.2 to 5.2) | 1(0 to 1) | 4.8(3.8 to 6.2) | 18(12.4 to 24.4) |
| Niue | 0(0 to 0) | 3.9(3 to 5.1) | 0(0 to 0) | 4.7(3.6 to 6) | 20.9(14.7 to 28.8) |
| Marshall Islands | 2(1 to 2) | 4(3.2 to 5.1) | 3(2 to 3) | 4.3(3.4 to 5.7) | 8.9(2.5 to 15.1) |
| Northern Mariana Islands | 2(1 to 3) | 3.8(2.9 to 4.9) | 2(1 to 2) | 4.5(3.5 to 5.8) | 18.7(11.3 to 25.4) |
| Palau | 1(0 to 1) | 3.9(3 to 5.1) | 1(1 to 1) | 4.8(3.7 to 6.1) | 21.1(14.7 to 28) |
| Papua New Guinea | 146(110 to 193) | 3.4(2.6 to 4.4) | 420(321 to 552) | 4(3.1 to 5.2) | 18.9(13 to 25.7) |
| Samoa | 6(5 to 8) | 3.7(2.9 to 4.8) | 10(7 to 13) | 4.4(3.4 to 5.7) | 18.4(11.7 to 24.8) |
| Solomon Islands | 14(10 to 18) | 4(3.2 to 5.1) | 31(24 to 40) | 4.5(3.6 to 5.8) | 12.5(5.8 to 17.9) |
| Tokelau | 0(0 to 0) | 3.6(2.7 to 4.7) | 0(0 to 0) | 4.4(3.4 to 5.8) | 24.6(17.3 to 32.5) |
| Tonga | 4(3 to 5) | 3.8(3 to 4.9) | 5(3 to 6) | 4.5(3.4 to 5.8) | 16.5(9.8 to 22.8) |
| Tuvalu | 0(0 to 0) | 3.7(2.8 to 4.7) | 1(0 to 1) | 4.5(3.5 to 5.8) | 22.7(15.2 to 30.6) |
| Vanuatu | 7(5 to 8) | 4.4(3.6 to 5.5) | 14(11 to 18) | 4.5(3.5 to 5.8) | 1.8(-4.4 to 7.4) |
| North Africa and Middle East | 26268(19514 to 34649) | 7(5.4 to 9.1) | 66665(50979 to 87066) | 10.4(8 to 13.5) | 48.2(44.1 to 52.5) |
| Afghanistan | 983(781 to 1229) | 8.3(6.9 to 10) | 3940(3053 to 5107) | 9.3(7.4 to 11.7) | 12.3(4.5 to 21.2) |
| Algeria | 2211(1619 to 2931) | 7.9(6.1 to 10.2) | 4623(3512 to 6055) | 10.9(8.2 to 14.2) | 37.1(29.7 to 45.6) |
| Bahrain | 42(31 to 56) | 7.5(5.7 to 9.8) | 161(125 to 206) | 11(8.4 to 14.4) | 46.7(38.7 to 54.9) |
| Egypt | 4107(3006 to 5468) | 6.9(5.1 to 9) | 10727(8061 to 14137) | 10.2(7.7 to 13.3) | 48.3(40.9 to 57.2) |
| Iran (Islamic Republic of) | 3676(2684 to 4851) | 5.6(4.3 to 7.3) | 8140(6272 to 10623) | 9.5(7.2 to 12.3) | 67.8(62.3 to 74.1) |
| Iraq | 1318(956 to 1761) | 7(5.3 to 9.1) | 5020(3754 to 6710) | 10.8(8.1 to 14.1) | 53.6(45.1 to 61.7) |
| Jordan | 311(225 to 431) | 7.3(5.5 to 9.8) | 1399(1053 to 1868) | 11(8.5 to 14.4) | 50.9(41.9 to 61.4) |
| Kuwait | 152(113 to 203) | 7.7(5.8 to 10.1) | 525(401 to 678) | 11.2(8.4 to 14.7) | 45.6(37.3 to 54.7) |
| Lebanon | 248(184 to 329) | 7.4(5.6 to 9.7) | 575(442 to 739) | 11.3(8.6 to 14.5) | 52.5(44 to 60.2) |
| Libya | 344(249 to 455) | 7.5(5.7 to 9.7) | 784(597 to 1035) | 10.7(8.1 to 14.2) | 43.5(36.8 to 51.8) |
| Morocco | 2097(1574 to 2778) | 7.5(5.8 to 9.6) | 3848(2955 to 4959) | 10.3(7.9 to 13.3) | 38.2(30.1 to 45.5) |

| | | | | | |
|---|---|---|---|---|---|
| Palestine | 174(128 to 229) | 8(6.1 to 10.3) | 597(445 to 794) | 11(8.5 to 14.4) | 38.1(30.6 to 46.3) |
| Oman | 152(111 to 205) | 7.3(5.4 to 9.7) | 568(431 to 758) | 11(8.4 to 14.4) | 51(41.8 to 60) |
| Qatar | 36(27 to 48) | 7.1(5.3 to 9.4) | 362(272 to 483) | 10.8(8.3 to 14) | 52(44 to 61.2) |
| Saudi Arabia | 1262(936 to 1686) | 7.1(5.4 to 9.5) | 4387(3391 to 5689) | 10.9(8.4 to 14.1) | 53.9(45.9 to 63.6) |
| Sudan | 1538(1128 to 2064) | 7.1(5.4 to 9.3) | 4564(3363 to 6059) | 10.1(7.8 to 13.1) | 42.7(35.1 to 51.4) |
| Syrian Arab Republic | 1150(848 to 1532) | 8(6.1 to 10.3) | 1734(1290 to 2297) | 11.1(8.3 to 14.4) | 38.3(29.9 to 46.7) |
| Tunisia | 690(514 to 922) | 7.5(5.7 to 9.9) | 1240(950 to 1590) | 10.8(8.3 to 14) | 44(36.8 to 51.6) |
| Turkey | 4517(3362 to 5970) | 6.9(5.2 to 9) | 8991(6872 to 11705) | 10.7(8.2 to 13.8) | 55.2(46.7 to 65.1) |
| United Arab Emirates | 149(111 to 198) | 7.2(5.4 to 9.4) | 997(764 to 1300) | 10.5(8 to 13.5) | 46.1(37.4 to 54.4) |
| Yemen | 1093(819 to 1442) | 7.7(6 to 9.9) | 3413(2532 to 4444) | 9.9(7.6 to 12.7) | 28.8(21.4 to 37.5) |
| South Asia | 76654(61590 to 96931) | 6.5(5.3 to 8.1) | 196227(154672 to 249562) | 10(8 to 12.6) | 53.5(45.9 to 61) |
| Bangladesh | 41110(33288 to 51852) | 36(29.8 to 44.2) | 87677(69429 to 109728) | 51.5(41.3 to 64.1) | 43(32.3 to 53.6) |
| Bhutan | 230(190 to 286) | 34.1(28.8 to 40.8) | 373(295 to 469) | 44.8(35.8 to 56) | 31.5(19.8 to 44.6) |
| India | 24903(19239 to 31790) | 2.7(2.1 to 3.4) | 84044(63988 to 110006) | 5.5(4.2 to 7.1) | 105.6(98.5 to 113.5) |
| Nepal | 6925(5760 to 8379) | 35.6(29.8 to 42.1) | 9907(8157 to 12175) | 30.3(25.4 to 36.5) | -14.9(-20.4 to -9.5) |
| Pakistan | 3486(2706 to 4452) | 2.8(2.3 to 3.5) | 14227(10897 to 18497) | 5.4(4.2 to 6.9) | 89.4(79.6 to 100.4) |
| Southern Sub-Saharan Africa | 3330(2449 to 4470) | 5.5(4.2 to 7.2) | 5855(4394 to 7749) | 6.9(5.2 to 9.1) | 26.2(22.4 to 30.6) |
| Botswana | 156(118 to 204) | 10.8(8.6 to 13.7) | 306(237 to 394) | 12(9.4 to 15.2) | 10.9(4.9 to 17.4) |
| Lesotho | 205(157 to 267) | 10.3(8.2 to 12.9) | 239(184 to 311) | 10.1(7.9 to 13) | -1.6(-6.9 to 4.1) |
| Namibia | 149(114 to 198) | 9.5(7.5 to 12.3) | 284(213 to 381) | 10.8(8.3 to 14.2) | 14.2(5.4 to 22.1) |
| South Africa | 1688(1222 to 2266) | 3.9(2.9 to 5.2) | 3300(2472 to 4356) | 5.6(4.2 to 7.4) | 43.5(38.9 to 48.7) |
| Eswatini | 87(64 to 117) | 9.6(7.6 to 12.4) | 135(102 to 180) | 10.4(8.1 to 13.7) | 8.6(1.3 to 15.1) |
| Zimbabwe | 1045(763 to 1419) | 9(7 to 11.8) | 1591(1189 to 2145) | 9.4(7.2 to 12.5) | 4.6(-0.4 to 10) |

| | | | | | |
|---|---|---|---|---|---|
| Western Sub-Saharan Africa | 6246(4676 to 8260) | 3(2.3 to 4) | 22773(16551 to 30697) | 4.5(3.4 to 6) | 48.7(43.6 to 54) |
| Benin | 205(152 to 273) | 4.1(3.1 to 5.4) | 746(541 to 1013) | 5.4(4.1 to 7.1) | 31.2(23 to 38.9) |
| Burkina Faso | 406(302 to 536) | 4.1(3.1 to 5.3) | 1315(974 to 1786) | 5.4(4.1 to 7.2) | 31.4(22 to 39.7) |
| Cameroon | 456(341 to 605) | 4.1(3.2 to 5.4) | 1826(1308 to 2468) | 5.5(4.1 to 7.3) | 33.9(25.6 to 42.3) |
| Cabo Verde | 16(11 to 22) | 4.3(3.2 to 5.7) | 37(28 to 50) | 6.1(4.5 to 8.2) | 42.7(35 to 51.4) |
| Chad | 270(207 to 354) | 4.3(3.4 to 5.6) | 909(662 to 1234) | 5.2(3.9 to 6.8) | 18.7(10.7 to 27.2) |
| Cote d'Ivoire | 540(399 to 715) | 10.8(9.1 to 12.6) | 358(327 to 387) | 10.8(9.7 to 11.9) | 0.1(-9.7 to 11.3) |
| Gambia | 47(36 to 63) | 4.4(3.5 to 5.7) | 144(104 to 195) | 5.6(4.2 to 7.4) | 26.2(17.4 to 34.7) |
| Ghana | 750(564 to 984) | 4.7(3.6 to 6.1) | 2091(1562 to 2797) | 5.9(4.5 to 7.8) | 27.2(19.2 to 34.6) |
| Guinea | 258(196 to 339) | 4.1(3.1 to 5.3) | 743(535 to 998) | 5.4(4 to 7.1) | 31.3(21.9 to 41.4) |
| Guinea-Bissau | 47(36 to 61) | 4.3(3.4 to 5.5) | 116(86 to 154) | 5.3(4.1 to 7) | 23.5(13.1 to 33.4) |
| Liberia | 81(60 to 106) | 4(3 to 5.3) | 311(228 to 423) | 5.7(4.3 to 7.6) | 42.4(33 to 52.3) |
| Mali | 372(282 to 487) | 4.2(3.2 to 5.4) | 1319(952 to 1776) | 5.5(4.1 to 7.1) | 31.8(22.5 to 40.5) |
| Mauritania | 91(67 to 120) | 4.1(3.1 to 5.4) | 264(192 to 362) | 5.9(4.4 to 7.8) | 41.5(33.1 to 50.4) |
| Niger | 368(282 to 476) | 4.4(3.4 to 5.6) | 1352(973 to 1812) | 5.4(4.1 to 7.1) | 24.3(14.4 to 35.3) |
| Nigeria | 1651(1204 to 2237) | 1.7(1.3 to 2.3) | 8132(5869 to 10981) | 3.4(2.5 to 4.5) | 101.4(94.7 to 109.7) |
| Sao Tome and Principe | 5(4 to 7) | 4.2(3.2 to 5.5) | 13(10 to 18) | 5.6(4.2 to 7.5) | 35.1(26.8 to 43.1) |
| Senegal | 353(263 to 464) | 4.3(3.3 to 5.6) | 931(675 to 1272) | 5.5(4.1 to 7.3) | 26.4(17.4 to 35.7) |
| Sierra Leone | 158(116 to 210) | 4.1(3.1 to 5.4) | 536(389 to 731) | 5.7(4.3 to 7.6) | 37.5(30.3 to 47.2) |
| Togo | 169(124 to 223) | 4.2(3.3 to 5.5) | 473(346 to 629) | 5.4(4.1 to 7.1) | 28.2(19.9 to 36.5) |
| Eastern Sub-Saharan Africa | 6672(4955 to 8948) | 3.2(2.5 to 4.3) | 22041(16104 to 29956) | 4.7(3.6 to 6.3) | 46.5(42.9 to 49.9) |
| Burundi | 249(186 to 334) | 4.3(3.3 to 5.6) | 797(591 to 1072) | 6.2(4.8 to 8.1) | 45.5(38.8 to 54) |
| Comoros | 26(20 to 34) | 5.1(4.1 to 6.6) | 53(40 to 70) | 6.8(5.3 to 8.9) | 32.7(24.5 to 42.4) |

| | | | | | |
|---|---|---|---|---|---|
| Djibouti | 28(21 to 38) | 5.1(3.9 to 6.7) | 85(64 to 113) | 6.7(5.1 to 8.8) | 33.1(25.2 to 39.4) |
| Eritrea | 139(104 to 186) | 4.2(3.3 to 5.5) | 484(361 to 653) | 6.4(4.9 to 8.4) | 49.7(41.2 to 57.7) |
| Ethiopia | 329(236 to 450) | 0.6(0.5 to 0.8) | 2420(1660 to 3411) | 1.9(1.3 to 2.6) | 207.6(178.6 to 243.7) |
| Kenya | 412(284 to 577) | 1.5(1 to 2) | 1423(991 to 1979) | 2.3(1.7 to 3.2) | 58.5(53.9 to 63.8) |
| Madagascar | 651(481 to 885) | 4.9(3.7 to 6.6) | 1849(1374 to 2521) | 6.2(4.7 to 8.2) | 25.7(17.5 to 33.9) |
| Malawi | 491(366 to 657) | 4.8(3.7 to 6.3) | 1370(1009 to 1819) | 6.5(5.1 to 8.5) | 36.4(29.9 to 43.7) |
| Mozambique | 697(530 to 918) | 5.1(4 to 6.5) | 2370(1793 to 3109) | 7.5(6 to 9.5) | 48.9(41.1 to 57.1) |
| Rwanda | 391(299 to 511) | 5.1(4 to 6.5) | 977(735 to 1328) | 6.9(5.4 to 9.2) | 35.7(25.9 to 47.4) |
| Somalia | 368(278 to 495) | 4.8(3.8 to 6.3) | 1359(1029 to 1811) | 6(4.7 to 7.8) | 24.3(18.6 to 30.6) |
| South Sudan | 283(205 to 386) | 4.3(3.2 to 5.7) | 577(419 to 773) | 5.6(4.2 to 7.3) | 30.4(23.7 to 36.9) |
| United Republic of Tanzania | 1309(965 to 1753) | 4.7(3.6 to 6.2) | 3971(2897 to 5378) | 6.5(4.9 to 8.6) | 38.6(32.3 to 45.7) |
| Uganda | 867(637 to 1175) | 4.7(3.7 to 6.2) | 2943(2153 to 3915) | 6.5(5 to 8.4) | 36.6(29.3 to 44.4) |
| Zambia | 425(311 to 573) | 4.8(3.7 to 6.2) | 1346(1000 to 1852) | 6.6(5.1 to 8.8) | 37.5(29.2 to 46.8) |
| Central Sub-Saharan Africa | 4762(3652 to 6076) | 7.9(6.3 to 10) | 15752(11998 to 20404) | 10.8(8.5 to 13.8) | 36.1(30.6 to 42.4) |
| Angola | 835(631 to 1074) | 7.4(5.8 to 9.3) | 3459(2588 to 4542) | 10.5(8.2 to 13.5) | 42.5(34.4 to 51.1) |
| Central African Republic | 254(202 to 320) | 8.8(7.3 to 10.8) | 544(422 to 685) | 9.3(7.5 to 11.5) | 5.2(-0.4 to 10.6) |
| Congo | 195(146 to 256) | 7.2(5.7 to 9.1) | 572(430 to 755) | 10(7.8 to 13.2) | 39.9(30.1 to 49.3) |
| Democratic Republic of the Congo | 3372(2578 to 4320) | 8.1(6.4 to 10.3) | 10803(8221 to 13950) | 11.1(8.8 to 14.1) | 36.3(28.8 to 44.3) |
| Equatorial Guinea | 31(24 to 39) | 7(5.6 to 8.7) | 176(129 to 240) | 10.4(7.9 to 13.7) | 48(34.2 to 61.7) |
| Gabon | 75(55 to 100) | 7(5.3 to 9.2) | 198(148 to 263) | 10.3(7.8 to 13.4) | 46.4(39.3 to 53.3) |

| Table S6: Incidence cases of appendicitis in 1990 and 2019 for both sexes and percentage change of age-standardised rates(ASR) by location | | | | | |
|---|---|---|---|---|---|
| | 1990 | | 2019 | | Percentage change in ASR from 1990 and 2019 |
| | Counts (95% UI) | Rate (95% UI) | Counts (95% UI) | Rate (95% UI) | |
| Global | 10821656(8420122 to 13838792) | 190.7(149.6 to 240.6) | 17698765(14101114 to 22324572) | 229.9(180.9 to 291) | 20.5(18.7 to 22.8) |
| High-income North America | 506556(404849 to 635595) | 181.3(143.1 to 229.8) | 571783(514543 to 636513) | 162.6(144.9 to 182.1) | -10.3(-21.7 to 4.1) |
| Canada | 49026(37996 to 63111) | 176(134 to 228.1) | 62889(49808 to 77949) | 177.3(136.3 to 224.9) | 0.8(-3.6 to 4.9) |
| Greenland | 98(75 to 129) | 164.3(125.1 to 213.6) | 102(80 to 128) | 179.2(139.4 to 229.9) | 9(3.2 to 14.7) |
| United States of America | 457420(366769 to 572772) | 181.8(143.9 to 229.9) | 508783(460698 to 560899) | 160.9(144.8 to 179.3) | -11.5(-23.8 to 4.2) |
| Australasia | 56661(43379 to 76361) | 277.2(210 to 369.6) | 74147(58363 to 94560) | 283.9(220.1 to 369.3) | 2.4(-2.1 to 6.4) |
| Australia | 46185(35096 to 62310) | 271.7(203.8 to 364.2) | 62291(48197 to 80789) | 281.4(213.8 to 370.7) | 3.5(-1.2 to 7.9) |
| New Zealand | 10476(8160 to 13514) | 303.3(236 to 387.9) | 11856(10078 to 14033) | 297.8(250.2 to 358.7) | -1.8(-12.2 to 9.5) |
| High-income Asia Pacific | 766628(570846 to 1001787) | 460(340.5 to 602.5) | 586290(463318 to 730184) | 448.1(339.7 to 572.3) | -2.6(-7.2 to 2.5) |
| Brunei Darussalam | 1201(886 to 1590) | 405.2(300.8 to 532.6) | 1926(1442 to 2504) | 433.8(321.3 to 565.9) | 7.1(1.8 to 13.2) |
| Japan | 547094(407298 to 716839) | 480.7(356 to 628.4) | 381574(305924 to 471666) | 451.2(348 to 567.3) | -6.1(-12.1 to 2.3) |
| Singapore | 13689(10317 to 18077) | 426.2(316.7 to 556.2) | 19344(14928 to 24709) | 434.3(322 to 573.4) | 1.9(-3.5 to 7) |
| Republic of Korea | 204644(151348 to 270223) | 417.2(311.1 to 545.9) | 183447(140794 to 233088) | 443.3(327.6 to 582.6) | 6.2(-0.1 to 12.1) |
| Western Europe | 909256(701607 to 1187303) | 248.6(190.5 to 327.3) | 997135(806633 to 1229567) | 278.5(220.2 to 352.8) | 12.1(6.6 to 18.3) |
| Andorra | 143(108 to 193) | 250.9(189.6 to 335.8) | 187(145 to 239) | 261.5(196.7 to 346.8) | 4.2(-0.3 to 9.1) |
| Austria | 26139(22246 to 30149) | 382.1(323.6 to 447.1) | 29878(27649 to 32085) | 438.1(398.4 to 475.7) | 14.6(2.1 to 29.5) |
| Belgium | 26217(20263 to 34549) | 281.7(215.9 to 372.2) | 32131(26824 to 37833) | 338.9(276.1 to 410.6) | 20.3(4.9 to 37.2) |

| | | | | | |
|---|---|---|---|---|---|
| Cyprus | 21383(16237 to 27587) | 215(160.4 to 281) | 22934(18717 to 27297) | 279.2(216.1 to 343.4) | 29.8(12.6 to 52.1) |
| Denmark | 12911(9961 to 16804) | 260.3(199 to 343.7) | 14097(10787 to 18066) | 274.7(204.7 to 364.8) | 5.5(1.2 to 9.7) |
| Finland | 13581(11513 to 16085) | 286.4(240.3 to 342.6) | 12949(11167 to 15159) | 269.9(228 to 324.2) | -5.8(-10.8 to -0.7) |
| France | 137645(104375 to 182090) | 248.8(187.9 to 328.4) | 147348(113334 to 190942) | 262.5(198.2 to 348.7) | 5.5(0.7 to 10.1) |
| Germany | 218704(167853 to 287626) | 296.7(222.3 to 393.7) | 259082(209604 to 313378) | 387.3(304 to 482.2) | 30.5(16.1 to 46.4) |
| Greece | 24065(18067 to 32077) | 242.5(180.9 to 323.6) | 20999(16128 to 27067) | 256.1(192.3 to 344.6) | 5.6(0.9 to 10.5) |
| Iceland | 701(529 to 926) | 271.8(205.6 to 356.3) | 962(752 to 1245) | 308(236.1 to 404.5) | 13.3(6.8 to 20.7) |
| Ireland | 9016(6784 to 12308) | 245.3(185 to 332.2) | 11388(8688 to 14738) | 258.5(193.5 to 345) | 5.4(0.2 to 9.7) |
| Israel | 12077(9046 to 16217) | 238(179.5 to 317.2) | 22332(16768 to 29608) | 253.6(189.7 to 336.6) | 6.5(1.2 to 11.4) |
| Italy | 113265(85082 to 149475) | 205.5(152.5 to 272.8) | 93251(76839 to 114002) | 221(175.2 to 277.1) | 7.5(-4.2 to 23.8) |
| Luxembourg | 1134(898 to 1437) | 330.7(258.6 to 420.5) | 1709(1489 to 1952) | 324.1(276.5 to 376.7) | -2(-13.9 to 14.2) |
| Malta | 750(564 to 993) | 208.5(155 to 277.5) | 859(711 to 1039) | 243.7(197.3 to 298.3) | 16.9(3.5 to 34.9) |
| Monaco | 60(47 to 79) | 244.6(182.9 to 327.6) | 72(56 to 91) | 254.2(191.7 to 337) | 3.9(-0.1 to 8.8) |
| Netherlands | 37941(28946 to 50409) | 257.9(193.8 to 343.9) | 39048(30089 to 50742) | 265.3(200.7 to 352) | 2.9(-1.8 to 8.1) |
| Norway | 11475(8793 to 15132) | 282.1(211.9 to 374) | 14786(11625 to 18701) | 309(236 to 399.3) | 9.5(5.3 to 13.9) |
| San Marino | 58(44 to 77) | 249.4(187 to 329.1) | 75(57 to 98) | 261.5(196.4 to 349.6) | 4.8(0.1 to 9.2) |
| Portugal | 14903(10950 to 19935) | 149(109.2 to 199.6) | 14763(12081 to 18156) | 177.7(140.6 to 225) | 19.3(6.1 to 35.3) |
| Spain | 95529(72371 to 126183) | 246.4(185.2 to 325.1) | 99780(77035 to 126469) | 264.2(198.7 to 352.3) | 7.2(2.6 to 11.8) |
| Sweden | 25141(19400 to 32640) | 326.2(246 to 427.1) | 30881(23991 to 39798) | 360.2(271 to 472.2) | 10.4(5.7 to 15.8) |
| Switzerland | 20396(17192 to 23975) | 321.1(267.5 to 378) | 24236(21072 to 27801) | 328.5(279.6 to 380.9) | 2.3(-1.7 to 7) |
| United Kingdom | 105167(82977 to 135990) | 198(152.9 to 257.5) | 123137(101989 to 151609) | 209.5(168.7 to 263.6) | 5.8(0.9 to 11.5) |
| Southern Latin America | 102773(78424 to 133813) | 204.2(157.2 to 264.3) | 201955(157808 to 254876) | 305.5(236.3 to 389.3) | 49.6(41.8 to 57.5) |
| Argentina | 66689(50937 to 86597) | 200.9(154.7 to 261.2) | 131161(101100 to 170436) | 291.3(221.6 to 378.9) | 45(36.3 to 54.5) |
| Chile | 29680(22510 to 39419) | 211.7(162.7 to 276.5) | 61126(49308 to 74679) | 345.3(274 to 426.2) | 63.1(50.2 to 78.4) |

| | | | | | |
|---|---|---|---|---|---|
| Uruguay | 6399(4964 to 8413) | 208.2(160.6 to 274.7) | 9658(7415 to 12353) | 292.8(221.8 to 382.4) | 40.6(33.3 to 48.2) |
| Eastern Europe | 483941(364145 to 628994) | 228.3(171.9 to 298.9) | 480413(370619 to 612175) | 273.2(202.6 to 354.6) | 19.7(17.1 to 22.5) |
| Belarus | 30976(23936 to 40215) | 308.8(236.9 to 404.7) | 25809(20097 to 32340) | 315.9(238.8 to 410.6) | 2.3(-2.4 to 8.4) |
| Estonia | 4582(3506 to 5832) | 309.9(234.4 to 399.4) | 3453(2672 to 4393) | 315.6(241.3 to 414.6) | 1.8(-3.3 to 7.3) |
| Latvia | 8122(6297 to 10324) | 323.3(245.7 to 418.5) | 5286(4304 to 6424) | 344.5(269.7 to 434.8) | 6.6(-1.1 to 14.9) |
| Lithuania | 11599(9065 to 14868) | 324.7(249.8 to 416.8) | 7898(6359 to 9610) | 342.3(267.6 to 430.8) | 5.4(-0.1 to 11.7) |
| Republic of Moldova | 13837(10667 to 17975) | 314.7(240.2 to 409.8) | 10070(7844 to 12946) | 309.3(234.3 to 405.6) | -1.7(-6.6 to 3.6) |
| Russian Federation | 290799(216936 to 377292) | 205.6(152.4 to 269.2) | 327060(250995 to 418478) | 266.3(197.1 to 347.6) | 29.5(26.4 to 33.9) |
| Ukraine | 124027(94964 to 160832) | 255.9(194 to 335.9) | 100837(77684 to 127872) | 275.8(207.3 to 355.8) | 7.8(2 to 14) |
| Central Europe | 243394(184520 to 315082) | 205(154.2 to 267.3) | 203543(167437 to 245338) | 220.3(175.2 to 271.3) | 7.5(0.6 to 15.7) |
| Albania | 7461(5508 to 10076) | 204.9(154.4 to 271.8) | 5263(3898 to 6789) | 216.6(158.3 to 282.8) | 5.7(-0.6 to 12.2) |
| Bosnia and Herzegovina | 9548(7175 to 12560) | 207(154.8 to 271.8) | 5753(4372 to 7306) | 208.3(152.8 to 274.2) | 0.6(-4.6 to 6.3) |
| Bulgaria | 17336(13135 to 22340) | 216.5(161 to 283.2) | 12531(9842 to 15510) | 225(168 to 292.4) | 3.9(-1.1 to 8.9) |
| Croatia | 28507(21997 to 37134) | 245.7(190.7 to 315.4) | 32135(25309 to 40529) | 319.8(244.6 to 411.6) | 30.1(22.8 to 37.1) |
| Czechia | 14342(10607 to 19047) | 107.5(82.2 to 140.3) | 40062(28940 to 53808) | 138.1(102.8 to 183.7) | 28.5(20.8 to 38.5) |
| Hungary | 21350(16459 to 27585) | 217.5(164.2 to 284.6) | 17933(13965 to 22756) | 225.3(167.7 to 297.1) | 3.6(-2.4 to 9) |
| North Macedonia | 4170(3036 to 5556) | 203.2(148.3 to 270.4) | 3966(2986 to 5110) | 212.6(155.6 to 279.8) | 4.6(-1.3 to 10.3) |
| Montenegro | 1328(991 to 1734) | 210.6(155.8 to 275.3) | 1175(887 to 1509) | 214.8(157.5 to 280) | 2(-2.6 to 8.6) |
| Poland | 66977(49694 to 88257) | 182.8(134.7 to 242.6) | 57580(49440 to 67184) | 187(155.5 to 224) | 2.3(-11.1 to 20.1) |
| Romania | 48093(36143 to 62339) | 210.1(157.2 to 271.9) | 33758(26014 to 43027) | 215.4(158.5 to 282.2) | 2.5(-2.3 to 8.2) |
| Serbia | 17301(13078 to 22404) | 192.8(144 to 252.4) | 17466(14275 to 20951) | 239.6(187.8 to 293) | 24.3(11.4 to 39.9) |
| Slovakia | 11472(8464 to 15120) | 219.8(161.8 to 288.5) | 11513(9145 to 14293) | 261.9(199.6 to 336.6) | 19.1(9 to 29) |
| Slovenia | 4356(3258 to 5671) | 231(171.6 to 301.3) | 4373(3894 to 4874) | 277.5(241.3 to 316.5) | 20.1(-1.6 to 50.7) |

| | | | | | |
|---|---|---|---|---|---|
| Central Asia | 181231(134467 to 237141) | 241.7(182.9 to 312.3) | 233029(175238 to 306969) | 245.3(184.6 to 323.5) | 1.5(-2.1 to 5.4) |
| Armenia | 8042(6001 to 10631) | 227.7(170.3 to 299.9) | 6797(5190 to 8797) | 252.9(187.6 to 335.4) | 11.1(5.6 to 16.9) |
| Azerbaijan | 18580(13819 to 24574) | 231.7(174 to 303.4) | 24200(18097 to 32279) | 240.3(178 to 323.6) | 3.7(-2.7 to 10.8) |
| Georgia | 11557(8630 to 14951) | 215.8(160.2 to 280.1) | 7516(5615 to 9662) | 244(179.5 to 322.4) | 13.1(7.4 to 19.9) |
| Kazakhstan | 42727(32082 to 55509) | 248.6(187.7 to 319.5) | 43912(33483 to 57276) | 248.4(186.2 to 327) | -0.1(-6.4 to 6.6) |
| Kyrgyzstan | 10605(7723 to 14159) | 219.5(163.5 to 289.3) | 16573(12333 to 22206) | 242.7(180.9 to 322.1) | 10.6(4.6 to 16.5) |
| Mongolia | 14480(11695 to 17600) | 554.1(457.8 to 661.5) | 11037(8753 to 13865) | 333.8(262.2 to 419.8) | -39.8(-46.1 to -30.7) |
| Tajikistan | 13499(9761 to 17870) | 225.5(170.1 to 293.5) | 24345(18051 to 32162) | 234.5(175.7 to 308) | 4(-1.3 to 9.4) |
| Turkmenistan | 9384(6813 to 12369) | 225.4(170 to 292) | 12603(9530 to 16512) | 243(182.3 to 321) | 7.8(2 to 13.3) |
| Uzbekistan | 52358(37936 to 70260) | 224.7(168 to 296.4) | 86048(64191 to 114727) | 240.1(179.1 to 316.2) | 6.8(1.2 to 13.3) |
| Central Latin America | 603007(451133 to 772387) | 310.4(239.5 to 389.7) | 900312(686867 to 1159909) | 358.2(271.1 to 460.1) | 15.4(11.8 to 19.4) |
| Colombia | 133573(97406 to 173850) | 366.2(276 to 468.7) | 199207(152593 to 254849) | 433(329.5 to 555.6) | 18.2(13 to 24.3) |
| Costa Rica | 13708(10019 to 18481) | 409.3(308.8 to 536.3) | 19118(14466 to 24647) | 422.7(313.6 to 554.7) | 3.3(-2.9 to 9.9) |
| El Salvador | 31783(25249 to 40058) | 496.1(403.3 to 609.6) | 25046(18822 to 32444) | 390.1(291.6 to 507.7) | -21.4(-31.1 to -11.6) |
| Guatemala | 60112(50325 to 71567) | 643.1(550.8 to 755.3) | 85531(66684 to 108889) | 426.8(337.6 to 534.6) | -33.6(-42.9 to -22.9) |
| Honduras | 25517(20211 to 32040) | 460(378 to 562.4) | 49980(38626 to 63459) | 458.3(362.7 to 573.2) | -0.4(-9.5 to 10.2) |
| Mexico | 222486(158837 to 298916) | 209.8(153.3 to 275.9) | 360798(267739 to 466826) | 289.1(214.4 to 378) | 37.8(34.2 to 42.2) |
| Nicaragua | 17228(12709 to 22392) | 369.7(286.1 to 465.8) | 28985(21612 to 37665) | 413.4(312.2 to 531.8) | 11.8(4.4 to 20.5) |
| Panama | 11466(8525 to 15000) | 425.5(325.8 to 546) | 17581(13198 to 22978) | 421.9(315.1 to 550.9) | -0.8(-6.8 to 6.2) |

| | | | | | |
|---|---|---|---|---|---|
| Venezuela (Bolivarian Republic of) | 87135(65269 to 111368) | 407.5(314.8 to 512) | 114066(86923 to 147136) | 416.6(313.9 to 543.2) | 2.2(-3.6 to 10.3) |
| Andean Latin America | 544791(458565 to 648440) | 1227.1(1050.4 to 1440.3) | 558662(456610 to 696711) | 852.4(697.7 to 1059.4) | -30.5(-36.6 to -22) |
| Bolivia (Plurinational State of) | 65145(54475 to 78615) | 884.7(751.2 to 1046.7) | 98270(79037 to 123814) | 785.6(636.9 to 986.5) | -11.2(-19.1 to -0.3) |
| Ecuador | 77754(64672 to 93944) | 692.1(587.9 to 822.8) | 156015(134818 to 182723) | 848.5(736.5 to 989.5) | 22.6(14.2 to 33.5) |
| Peru | 401892(338053 to 477893) | 1575.4(1345.1 to 1856.4) | 304376(238232 to 395095) | 879.7(688 to 1137.6) | -44.2(-51.5 to -33.6) |
| Caribbean | 84394(65093 to 109139) | 220(172.9 to 281.1) | 132078(102639 to 169890) | 282.9(219.4 to 361.9) | 28.6(25.1 to 31.8) |
| Antigua and Barbuda | 149(110 to 196) | 229.5(172.2 to 299.8) | 269(206 to 346) | 309.9(236.2 to 400.6) | 35.1(27.8 to 42.4) |
| Bahamas | 639(475 to 850) | 217.7(164.7 to 284.5) | 1127(859 to 1452) | 295.7(223.2 to 384.8) | 35.8(29.6 to 41.9) |
| Barbados | 589(445 to 773) | 224.5(170.5 to 293.7) | 799(620 to 1026) | 306.3(234 to 402.4) | 36.4(29.7 to 43.8) |
| Belize | 433(315 to 579) | 203.5(154.5 to 266.8) | 1283(951 to 1690) | 278.3(211.8 to 361.1) | 36.7(30.2 to 44.5) |
| Bermuda | 133(101 to 171) | 227.4(171.1 to 294.7) | 166(130 to 210) | 324.4(244 to 427) | 42.6(35.8 to 49.8) |
| Cuba | 1483(1090 to 2012) | 189(139.2 to 256.6) | 2314(1922 to 2820) | 204.2(166.3 to 252.3) | 8(-3.9 to 24.6) |
| Dominica | 171(127 to 230) | 216.7(165 to 287.3) | 181(137 to 235) | 273.8(205.7 to 354.4) | 26.4(19.8 to 32.4) |
| Dominican Republic | 18739(14314 to 24373) | 222.9(174.1 to 284.5) | 34351(26280 to 44785) | 303.4(232.4 to 395.3) | 36.1(27.5 to 44.4) |
| Grenada | 191(139 to 252) | 205.3(154.8 to 265.8) | 300(227 to 393) | 289.5(219 to 378) | 41(34.6 to 48.7) |
| Guyana | 1959(1539 to 2479) | 222(178.1 to 276.1) | 2443(1925 to 3125) | 297.3(235 to 375) | 34(26.8 to 41.6) |
| Haiti | 11128(8828 to 13821) | 160.3(130.2 to 196) | 30099(23624 to 38195) | 220.3(175.5 to 276.3) | 37.4(27.7 to 47.6) |
| Jamaica | 5886(4362 to 7863) | 222.6(169.5 to 290.6) | 8539(6440 to 11145) | 293.5(220.5 to 383.3) | 31.8(25.5 to 38.4) |
| Puerto Rico | 8469(6322 to 11268) | 227.4(170.3 to 302.5) | 9353(7159 to 12183) | 311.3(230.9 to 413) | 36.9(30.4 to 43.7) |

| | | | | | |
|---|---|---|---|---|---|
| Saint Kitts and Nevis | 108(81 to 143) | 245.6(191.8 to 317.1) | 188(146 to 244) | 325.6(249.2 to 425.6) | 32.5(25.1 to 39.8) |
| Saint Lucia | 328(241 to 435) | 212.8(161.4 to 277.9) | 507(391 to 661) | 300.1(229.4 to 394.9) | 41(34.4 to 49) |
| Saint Vincent and the Grenadines | 266(193 to 354) | 210.4(158.6 to 275) | 308(237 to 397) | 281.5(214.3 to 360.9) | 33.8(28.1 to 40.5) |
| Suriname | 872(652 to 1145) | 202.1(155.4 to 259.6) | 1592(1199 to 2061) | 279.4(209.7 to 361.7) | 38.2(30.7 to 45.4) |
| Trinidad and Tobago | 2736(2031 to 3598) | 209.3(157.6 to 273.4) | 3677(2818 to 4778) | 288.7(220.1 to 379.5) | 37.9(31.4 to 45.7) |
| United States Virgin Islands | 280(217 to 358) | 255.7(199.5 to 328.8) | 286(228 to 364) | 326.6(254.8 to 423.3) | 27.7(21.5 to 34.2) |
| Tropical Latin America | 180230(138395 to 229170) | 106.1(82.6 to 133.2) | 298011(243250 to 366750) | 135.6(109.7 to 167.8) | 27.8(22.6 to 34.3) |
| Brazil | 166814(128376 to 212438) | 100.2(78.1 to 125.9) | 281107(229556 to 344854) | 132.6(107 to 164) | 32.4(26.1 to 39.7) |
| Paraguay | 13416(10364 to 17153) | 334.7(272.2 to 412.4) | 16904(13424 to 21862) | 235.8(189.5 to 297.2) | -29.6(-32.9 to -25.7) |
| East Asia | 2187023(1639403 to 2842758) | 164(125.7 to 210.7) | 2565267(2034140 to 3149307) | 176.2(138.5 to 220) | 7.4(1.8 to 13.6) |
| China | 2127289(1593409 to 2767510) | 164.6(126.1 to 212) | 2486787(1970764 to 3055446) | 177(139 to 221) | 7.6(1.9 to 13.9) |
| Democratic People's Republic of Korea | 35025(27079 to 43977) | 169.4(132.4 to 211.5) | 43909(34293 to 54661) | 163.1(126.5 to 207.2) | -3.7(-8.9 to 1.9) |
| Taiwan (Province of China) | 24709(18900 to 31823) | 119.1(92.3 to 149.9) | 34571(29139 to 40546) | 139.9(117.3 to 167.1) | 17.5(7.5 to 31.1) |
| Southeast Asia | 640386(486433 to 839944) | 124.3(96.3 to 159.9) | 1146656(892434 to 1477241) | 166.5(129.2 to 216.3) | 33.9(30.4 to 37.4) |
| Cambodia | 25562(19710 to 32486) | 229.2(183 to 284.8) | 46189(35478 to 60342) | 265.9(206.6 to 343.7) | 16(8.2 to 24) |
| Indonesia | 101300(73664 to 135438) | 48.1(35.9 to 63) | 248461(184521 to 328902) | 92.5(68.7 to 121.8) | 92.5(87.1 to 99.4) |

| | | | | | |
|---|---|---|---|---|---|
| Lao People's Democratic Republic | 11056(8739 to 13962) | 240.2(195.4 to 296.6) | 20906(16125 to 27194) | 269.2(210.8 to 344.7) | 12.1(3.9 to 22.2) |
| Malaysia | 34749(26240 to 45628) | 186.9(144.7 to 241.4) | 79149(60778 to 103885) | 242.5(186.5 to 312.4) | 29.7(23.3 to 37) |
| Maldives | 366(263 to 494) | 157.7(118.7 to 208.1) | 1134(872 to 1480) | 219.5(165.3 to 285.5) | 39.2(32.4 to 47.6) |
| Mauritius | 1985(1480 to 2690) | 165.9(125.2 to 221.4) | 2700(2084 to 3467) | 216.8(164.2 to 282.7) | 30.6(24.1 to 39) |
| Myanmar | 99057(77201 to 126068) | 217.8(173.6 to 273.2) | 138736(107133 to 180111) | 244.5(188.7 to 316.4) | 12.3(4.3 to 19.3) |
| Philippines | 42881(31339 to 57728) | 59.4(44.4 to 78.9) | 127151(94162 to 169771) | 105.1(78.1 to 138.9) | 77(73.3 to 81.1) |
| Sri Lanka | 30982(22921 to 40947) | 166.3(124.6 to 217.6) | 47869(36276 to 61851) | 220.3(166.3 to 287) | 32.5(25.7 to 39.5) |
| Seychelles | 148(113 to 194) | 195.8(152.8 to 251.7) | 260(205 to 324) | 256.2(199.4 to 326.2) | 30.9(23.2 to 40.3) |
| Thailand | 145340(110711 to 189417) | 227.1(176.5 to 290.8) | 163025(127828 to 205760) | 246.1(189.1 to 319.7) | 8.4(1.8 to 14.5) |
| Timor-Leste | 1710(1326 to 2172) | 205.7(163.3 to 259.1) | 3865(2929 to 5145) | 261.3(206.3 to 337.3) | 27(19.8 to 34.7) |
| Viet Nam | 144398(109465 to 189041) | 198.3(154.8 to 254.8) | 265708(211404 to 331248) | 278(218.5 to 349.4) | 40.2(31.3 to 51.4) |
| Oceania | 6399(4840 to 8371) | 94(73.1 to 120.3) | 15219(11665 to 19748) | 109.2(85 to 140.3) | 16.1(12.1 to 21) |
| American Samoa | 51(39 to 67) | 102.4(79.4 to 131.8) | 67(51 to 86) | 118.7(92.3 to 152.5) | 15.9(9.8 to 22.9) |
| Cook Islands | 19(14 to 26) | 98.4(73.3 to 128.4) | 20(15 to 25) | 115.4(88 to 151.3) | 17.3(10.6 to 24.9) |
| Micronesia (Federated States of) | 110(84 to 141) | 103.5(81.7 to 130.5) | 130(100 to 168) | 120.2(93.5 to 153.4) | 16.1(9.6 to 22.6) |
| Fiji | 832(626 to 1088) | 101.8(78.5 to 130.6) | 1135(875 to 1446) | 121.2(93.7 to 154.5) | 19(12.5 to 25.5) |
| Guam | 146(110 to 189) | 98.7(75.2 to 126.9) | 193(148 to 249) | 115.6(88.5 to 149.3) | 17.1(10.3 to 23.7) |
| Kiribati | 81(63 to 103) | 105.2(83.8 to 131.6) | 136(105 to 174) | 108.6(84.7 to 136.9) | 3.2(-2.1 to 8.6) |
| Nauru | 11(8 to 14) | 106.6(82.9 to 136.8) | 14(11 to 19) | 125.7(98.4 to 161.7) | 17.9(12.2 to 24.2) |
| Niue | 2(2 to 3) | 101.7(78.7 to 131.9) | 2(2 to 2) | 122.8(93.9 to 157.9) | 20.8(14.5 to 28.7) |
| Marshall Islands | 49(37 to 64) | 105.2(83.7 to 134.2) | 69(54 to 91) | 114.5(89.8 to 148.1) | 8.8(2.4 to 15) |

| | | | | | |
|---|---|---|---|---|---|
| Northern Mariana Islands | 51(38 to 68) | 99.2(75.6 to 129) | 49(38 to 63) | 117.7(91 to 151.4) | 18.7(11.3 to 25.3) |
| Palau | 17(13 to 23) | 103.4(78.4 to 132.9) | 23(18 to 28) | 125.1(96.8 to 159.6) | 21(14.6 to 28) |
| Papua New Guinea | 3859(2901 to 5056) | 89.4(68.9 to 114.9) | 11089(8441 to 14397) | 106.1(82.1 to 137.2) | 18.8(12.8 to 25.5) |
| Samoa | 169(126 to 224) | 98.5(76.3 to 125.9) | 256(194 to 336) | 116.5(90.4 to 149.6) | 18.2(11.5 to 24.7) |
| Solomon Islands | 360(277 to 469) | 106.3(84.8 to 134.5) | 816(628 to 1048) | 119.2(93.6 to 151.6) | 12.2(5.5 to 17.7) |
| Tokelau | 2(1 to 2) | 93.9(72.1 to 123.3) | 2(1 to 2) | 116.7(89.3 to 150.5) | 24.3(17 to 32.1) |
| Tonga | 100(75 to 129) | 101.2(77.8 to 129.2) | 122(91 to 157) | 117.7(90.2 to 150.9) | 16.3(9.6 to 22.6) |
| Tuvalu | 9(7 to 12) | 96.7(74.5 to 123.4) | 14(11 to 19) | 118.4(91.4 to 151.8) | 22.5(14.9 to 30.3) |
| Vanuatu | 176(138 to 222) | 117.1(94.9 to 144.7) | 364(281 to 471) | 119.2(93.4 to 151.8) | 1.8(-4.5 to 7.4) |
| North Africa and Middle East | 693835(514641 to 913057) | 183.5(140.1 to 237) | 1745503(1329173 to 2271525) | 271.6(206.1 to 350.9) | 48(43.9 to 52.2) |
| Afghanistan | 26137(20722 to 32766) | 218.9(181.3 to 265.7) | 104471(81520 to 133394) | 245.2(196.6 to 306.3) | 12(4.4 to 20.9) |
| Algeria | 58397(42744 to 76369) | 208(159.4 to 265.4) | 120880(91024 to 157230) | 284.6(212.7 to 370.8) | 36.9(29.4 to 45.3) |
| Bahrain | 1105(828 to 1466) | 197.7(149.3 to 258.7) | 4188(3257 to 5346) | 289(219.2 to 378.4) | 46.2(38.3 to 54.2) |
| Egypt | 108283(79411 to 143562) | 180.1(135.5 to 236.9) | 281600(211921 to 372590) | 266.9(202 to 348.8) | 48.2(40.8 to 57) |
| Iran (Islamic Republic of) | 97503(71047 to 129219) | 148.6(111.9 to 192.5) | 212552(163469 to 275493) | 248.4(188 to 321.4) | 67.2(61.7 to 73.4) |
| Iraq | 34869(25107 to 46085) | 183.7(138.2 to 239.5) | 131682(98119 to 176349) | 282.1(212.5 to 367.9) | 53.6(45.1 to 61.7) |
| Jordan | 8215(5925 to 11258) | 191.6(144.4 to 256.3) | 36686(27549 to 48617) | 288.9(220 to 375.4) | 50.8(41.7 to 61.1) |
| Kuwait | 3982(2948 to 5270) | 202.1(151.4 to 263.5) | 13614(10467 to 17650) | 294.4(221.1 to 386.5) | 45.6(37.3 to 54.6) |
| Lebanon | 6520(4824 to 8668) | 193.3(146.4 to 254.4) | 15021(11578 to 19219) | 294.7(224.7 to 378.5) | 52.4(43.9 to 60) |
| Libya | 9094(6595 to 12082) | 195.8(148.8 to 253.1) | 20448(15574 to 26975) | 280.8(212 to 370.9) | 43.4(36.7 to 51.7) |
| Morocco | 55272(41362 to 72366) | 196(150 to 252.2) | 100696(76990 to 130121) | 270.3(206.7 to 348.6) | 37.9(29.8 to 45.2) |
| Palestine | 4607(3387 to 6037) | 209.9(160.6 to 269.1) | 15708(11740 to 20719) | 289.6(220.5 to 376.5) | 38(30.5 to 46.2) |

| | | | | | |
|---|---|---|---|---|---|
| Oman | 4007(2923 to 5379) | 191.5(141.8 to 253.1) | 14753(11242 to 19556) | 289(219.2 to 375.2) | 50.9(41.7 to 59.8) |
| Qatar | 930(692 to 1242) | 186.8(139.7 to 248.1) | 9338(6969 to 12446) | 283.7(216.4 to 368.2) | 51.9(44 to 61.2) |
| Saudi Arabia | 33256(24678 to 44170) | 186.2(140.7 to 246) | 113939(88241 to 147467) | 286.3(218.4 to 369.1) | 53.7(45.9 to 63.5) |
| Sudan | 40730(29724 to 54289) | 186.9(142.6 to 243.7) | 120298(88747 to 158686) | 266(202.8 to 343) | 42.3(34.7 to 50.9) |
| Syrian Arab Republic | 30464(22445 to 40410) | 210.3(160.9 to 271.2) | 45533(33851 to 60546) | 290.5(217.9 to 379.3) | 38.1(29.8 to 46.5) |
| Tunisia | 18152(13549 to 24081) | 197.2(150.1 to 258.7) | 32354(24669 to 41602) | 283.8(215.5 to 367.6) | 43.9(36.7 to 51.5) |
| Turkey | 118816(88719 to 157039) | 180.3(136.5 to 235.8) | 233992(178596 to 304678) | 279.6(213.3 to 362.8) | 55.1(46.6 to 65) |
| United Arab Emirates | 3883(2896 to 5176) | 188.6(141.2 to 246.2) | 25790(19858 to 33486) | 275.2(209.6 to 356.5) | 45.9(37.1 to 54.3) |
| Yemen | 29147(21765 to 38354) | 202.6(159 to 260.6) | 90189(67070 to 118136) | 260.2(199.5 to 333.3) | 28.4(21.1 to 37.1) |
| South Asia | 2067302(1659767 to 2605802) | 175.7(144.2 to 216.3) | 5214009(4114157 to 6620999) | 266.7(212.7 to 334.9) | 51.8(44.3 to 59.1) |
| Bangladesh | 1086795(878628 to 1373311) | 943.8(779.9 to 1156.2) | 2295561(1818278 to 2870740) | 1349.8(1092.2 to 1673.1) | 43(32.1 to 53.6) |
| Bhutan | 6081(5002 to 7541) | 893.1(754.5 to 1074.1) | 9738(7713 to 12242) | 1174.4(942.1 to 1459.6) | 31.5(19.8 to 44.7) |
| India | 693665(536610 to 880080) | 75.3(59.8 to 94.2) | 2262369(1719023 to 2935055) | 149.4(114.7 to 191.7) | 98.4(91.2 to 106.2) |
| Nepal | 183008(151680 to 220119) | 931.8(781 to 1103.9) | 259904(214401 to 318709) | 792.8(667.9 to 952.7) | -14.9(-20.1 to -9.5) |
| Pakistan | 97755(75912 to 123791) | 80.3(65 to 98.8) | 386438(297740 to 498451) | 147(115.3 to 186.8) | 83.1(73.3 to 93.9) |
| Southern Sub-Saharan Africa | 88704(65069 to 118927) | 145.4(110.3 to 191.8) | 154821(116584 to 203979) | 183(138.5 to 241) | 25.9(22.1 to 30.2) |
| Botswana | 4132(3139 to 5402) | 282.8(225.8 to 361.2) | 8008(6240 to 10262) | 313.1(246 to 398.7) | 10.7(4.7 to 17.2) |
| Lesotho | 5414(4150 to 7067) | 269.3(214.4 to 339.7) | 6267(4845 to 8171) | 264.9(208 to 340.6) | -1.6(-7 to 4) |
| Namibia | 3933(2984 to 5246) | 249.3(196.9 to 323.3) | 7460(5576 to 9926) | 284.1(216.8 to 369.7) | 14(5.2 to 21.9) |
| South Africa | 45225(32701 to 60694) | 104.9(78 to 138.7) | 87538(65357 to 115647) | 149.6(111.6 to 198) | 42.6(38.2 to 47.6) |

| | | | | | |
|---|---|---|---|---|---|
| Eswatini | 2307(1723 to 3089) | 252.1(197.5 to 326.1) | 3555(2698 to 4723) | 273.7(212.3 to 357) | 8.6(1.2 to 15) |
| Zimbabwe | 27692(20202 to 37641) | 236.5(183.2 to 309.5) | 41992(31303 to 56477) | 247.7(189 to 326.7) | 4.7(-0.3 to 10.1) |
| Western Sub-Saharan Africa | 168713(126222 to 221418) | 81.4(62.6 to 106.3) | 611213(445004 to 824269) | 120.1(89.7 to 158.9) | 47.6(42.4 to 52.8) |
| Benin | 5483(4056 to 7317) | 108.3(82.6 to 142.3) | 19804(14435 to 26749) | 141.6(106.7 to 187.4) | 30.8(22.6 to 38.3) |
| Burkina Faso | 10877(8085 to 14343) | 108.2(82.6 to 139.8) | 34961(25891 to 47083) | 141.6(108.1 to 189.5) | 30.9(21.5 to 39.2) |
| Cameroon | 12137(9040 to 16000) | 109.2(83.6 to 142.4) | 48314(34507 to 64836) | 145.5(107.5 to 192.9) | 33.3(25.1 to 41.6) |
| Cabo Verde | 426(304 to 582) | 112.4(82.6 to 150.6) | 970(721 to 1311) | 160(119 to 214.5) | 42.3(34.6 to 51) |
| Chad | 7220(5508 to 9420) | 115.2(90.4 to 147.7) | 24296(17751 to 32811) | 136.2(103.7 to 179.8) | 18.3(10.3 to 26.8) |
| Cote d'Ivoire | 12620(10771 to 14582) | 282.6(237.7 to 330.4) | 9297(8517 to 10059) | 282.8(253.8 to 311.1) | 0.1(-9.7 to 11.4) |
| Gambia | 1262(945 to 1652) | 117.5(90.8 to 151.4) | 3820(2763 to 5141) | 147.7(110.9 to 194) | 25.8(17.1 to 34.2) |
| Ghana | 19917(14949 to 25996) | 122.9(95.7 to 158.5) | 55052(41014 to 74384) | 155.7(119 to 206) | 26.7(18.8 to 34.1) |
| Guinea | 6894(5237 to 8955) | 108.6(83.3 to 138.8) | 19753(14196 to 26583) | 142(105.8 to 186.4) | 30.8(21.5 to 40.9) |
| Guinea-Bissau | 1258(966 to 1630) | 114.3(91 to 144.5) | 3063(2272 to 4024) | 140.4(107.1 to 182) | 22.8(12.5 to 32.6) |
| Liberia | 2172(1594 to 2836) | 106.1(80.5 to 137.8) | 8235(6042 to 11159) | 150.4(113 to 201.1) | 41.7(32.4 to 51.4) |
| Mali | 9947(7540 to 13011) | 110(85.5 to 141.6) | 35065(25224 to 46930) | 144.4(108.5 to 187.4) | 31.3(22 to 39.7) |
| Mauritania | 2415(1777 to 3191) | 109.6(82.8 to 143.4) | 6976(5051 to 9600) | 154(115.6 to 204.5) | 40.5(32.4 to 49.3) |
| Niger | 9859(7599 to 12735) | 115.6(91.5 to 146.4) | 36104(26117 to 48405) | 143(107.4 to 188.1) | 23.7(13.9 to 34.6) |
| Nigeria | 46218(33805 to 62195) | 47.3(35.3 to 62.8) | 223056(161039 to 301567) | 92.8(68.7 to 122.7) | 96.3(90.2 to 104.1) |
| Sao Tome and Principe | 146(107 to 197) | 110.1(84.4 to 144.5) | 347(253 to 470) | 148.1(109.8 to 195.5) | 34.5(26.2 to 42.4) |
| Senegal | 9424(6991 to 12338) | 114.7(88.1 to 147.5) | 24641(17840 to 33455) | 144.4(108.6 to 191.4) | 25.9(17 to 35) |
| Sierra Leone | 4209(3098 to 5539) | 109(81.7 to 141.8) | 14173(10263 to 19146) | 149.4(113 to 199.3) | 37.1(29.9 to 46.7) |
| Togo | 4501(3299 to 5962) | 112.1(86.3 to 145.6) | 12512(9174 to 16564) | 143.3(108.2 to 187) | 27.8(19.7 to 36.1) |

| | | | | | |
|---|---|---|---|---|---|
| Eastern Sub-Saharan Africa | 180185(133926 to 241754) | 87.1(67 to 113.2) | 591822(432475 to 797031) | 127(96.7 to 166.9) | 45.7(42.2 to 49) |
| Burundi | 6654(4935 to 8829) | 113.1(87 to 147.1) | 21173(15729 to 28386) | 164(126.5 to 215) | 45(38.4 to 53.3) |
| Comoros | 692(522 to 911) | 135.5(106.4 to 173.6) | 1387(1048 to 1841) | 178.9(138.5 to 234.7) | 32.1(23.7 to 41.5) |
| Djibouti | 736(540 to 993) | 133.4(102.1 to 175.1) | 2245(1684 to 2985) | 176.8(133.3 to 232.7) | 32.5(24.8 to 38.6) |
| Eritrea | 3725(2796 to 4941) | 112.9(88.2 to 146.1) | 12810(9537 to 17316) | 167.8(129.6 to 220) | 48.6(40.4 to 56.3) |
| Ethiopia | 10376(7483 to 14042) | 19.5(14.9 to 25.3) | 69516(47854 to 96524) | 53.8(38.8 to 73.3) | 176.1(150.9 to 207.1) |
| Kenya | 11826(8203 to 16357) | 42.5(30.5 to 57.5) | 39717(27742 to 54753) | 65.4(47.4 to 88.7) | 54(50 to 58.7) |
| Madagascar | 17303(12754 to 23497) | 129.8(98.5 to 174) | 48938(36285 to 66388) | 162.9(124.4 to 216.6) | 25.5(17.4 to 33.7) |
| Malawi | 13063(9752 to 17269) | 126.6(98.1 to 166.3) | 36340(26684 to 48509) | 172.1(133.6 to 222.6) | 35.9(29.7 to 43.2) |
| Mozambique | 18683(14250 to 24340) | 134.5(107.7 to 171.6) | 63027(47593 to 82392) | 199(158 to 250.4) | 47.9(40.3 to 56) |
| Rwanda | 10444(8010 to 13639) | 135.2(106.8 to 171.3) | 25796(19312 to 34867) | 182(141 to 241.5) | 34.6(25.1 to 46.3) |
| Somalia | 9876(7451 to 13157) | 128.5(101.8 to 168.1) | 36194(27410 to 48246) | 159.4(125.3 to 206.1) | 24.1(18.4 to 30.5) |
| South Sudan | 7507(5439 to 10159) | 113.4(85.4 to 150.1) | 15377(11097 to 20728) | 147.5(111.3 to 191.9) | 30.1(23.5 to 36.6) |
| United Republic of Tanzania | 34784(25551 to 46605) | 122.9(93.6 to 162.6) | 105167(76553 to 142373) | 169.9(128.6 to 225.6) | 38.2(32 to 45.5) |
| Uganda | 23060(16873 to 31006) | 125.3(96.9 to 163.1) | 78076(56588 to 104088) | 170.3(130.6 to 221.4) | 35.9(28.6 to 43.7) |
| Zambia | 11322(8318 to 15238) | 127.1(98.7 to 162.5) | 35588(26487 to 48935) | 173.7(133.7 to 230.5) | 36.7(28.5 to 45.9) |
| Central Sub-Saharan Africa | 126246(96656 to 160464) | 209.3(166.2 to 263.2) | 416895(315480 to 538242) | 284.3(224.9 to 361.7) | 35.8(30.3 to 42.2) |
| Angola | 22150(16731 to 28257) | 194.1(152.8 to 243.1) | 91755(68655 to 120703) | 275.7(214.3 to 353.4) | 42(33.8 to 50.5) |
| Central African Republic | 6744(5370 to 8435) | 233(191.3 to 282.4) | 14416(11183 to 18167) | 245.3(197.4 to 303) | 5.3(-0.2 to 10.7) |
| Congo | 5155(3868 to 6708) | 188.8(148.8 to 239.8) | 15090(11355 to 19859) | 263.5(202.9 to 344.5) | 39.6(29.7 to 49) |

| | | | | | |
|---|---|---|---|---|---|
| Democratic Republic of the Congo | 89392(68435 to 114246) | 213.9(169.7 to 271.4) | 285813(217758 to 368161) | 291.2(230 to 369.3) | 36.1(28.7 to 44) |
| Equatorial Guinea | 832(648 to 1038) | 185.3(149 to 229.6) | 4631(3385 to 6277) | 272(207.2 to 356.3) | 46.8(33 to 60.4) |
| Gabon | 1973(1443 to 2617) | 184.4(140.6 to 241.8) | 5190(3888 to 6879) | 269.6(204.5 to 350.6) | 46.2(39 to 53.2) |

| Table S7: Years lived with disability (YLDs) of appendicitis in 1990 and 2019 for both sexes and percentage change of age-standardised rates(ASR) by location | | | | | |
|---|---|---|---|---|---|
| | 1990 | | 2019 | | Percentage change in ASR from 1990 and 2019 |
| | Counts (95% UI) | Rate (95% UI) | Counts (95% UI) | Rate (95% UI) | |
| Global | 128706(82721 to 188292) | 2.3(1.5 to 3.3) | 211113(137041 to 303366) | 2.7(1.8 to 3.9) | 20.4(16.2 to 25.1) |
| High-income North America | 6156(4024 to 8892) | 2.2(1.4 to 3.2) | 7057(4756 to 9721) | 2(1.3 to 2.8) | -8.9(-21.9 to 6.8) |
| Canada | 602(372 to 903) | 2.2(1.3 to 3.3) | 776(493 to 1160) | 2.2(1.3 to 3.3) | 0.9(-21.3 to 30.4) |
| Greenland | 1(1 to 2) | 2(1.2 to 3.1) | 1(1 to 2) | 2.2(1.4 to 3.2) | 8.6(-14.1 to 36.5) |
| United States of America | 5552(3635 to 8001) | 2.2(1.4 to 3.2) | 6280(4187 to 8721) | 2(1.3 to 2.7) | -10(-23.7 to 6.2) |
| Australasia | 671(406 to 1049) | 3.3(2 to 5.1) | 882(546 to 1332) | 3.4(2 to 5.2) | 2.4(-22.8 to 35.2) |
| Australia | 547(328 to 862) | 3.2(1.9 to 5) | 741(450 to 1144) | 3.3(2 to 5.3) | 3.5(-25.7 to 42.7) |
| New Zealand | 124(76 to 190) | 3.6(2.2 to 5.5) | 141(89 to 204) | 3.5(2.2 to 5.1) | -2(-27.2 to 34.8) |
| High-income Asia Pacific | 9194(5824 to 13631) | 5.5(3.5 to 8.2) | 7111(4538 to 10230) | 5.4(3.4 to 7.9) | -2.2(-11.7 to 8.9) |
| Brunei Darussalam | 14(8 to 23) | 4.8(2.8 to 7.6) | 23(14 to 36) | 5.2(3.2 to 8.3) | 6.8(-21.5 to 39.3) |
| Japan | 6557(4121 to 9639) | 5.7(3.6 to 8.5) | 4613(3014 to 6651) | 5.4(3.5 to 7.9) | -5.9(-14 to 4.3) |
| Singapore | 165(95 to 260) | 5.1(3 to 8) | 234(143 to 357) | 5.2(3.1 to 8.1) | 1.8(-23 to 37.9) |
| Republic of Korea | 2458(1454 to 3803) | 5(3 to 7.7) | 2241(1356 to 3395) | 5.3(3.2 to 8.4) | 6.9(-19.7 to 43.9) |
| Western Europe | 10892(6969 to 16108) | 3(1.9 to 4.4) | 11990(7743 to 17155) | 3.3(2.1 to 4.8) | 12(0.6 to 25.9) |
| Andorra | 2(1 to 3) | 3(1.8 to 4.8) | 2(1 to 3) | 3.1(1.8 to 4.7) | 3.6(-26.2 to 46.4) |
| Austria | 314(198 to 461) | 4.5(2.8 to 6.8) | 359(229 to 515) | 5.2(3.3 to 7.5) | 14.2(-15 to 58.1) |
| Belgium | 315(186 to 483) | 3.4(1.9 to 5.2) | 385(235 to 564) | 4(2.4 to 5.9) | 19.6(-13 to 74) |
| Cyprus | 259(157 to 408) | 2.6(1.5 to 4.1) | 279(174 to 418) | 3.4(2 to 5.3) | 29.3(-9.8 to 87.2) |
| Denmark | 154(93 to 237) | 3.1(1.8 to 4.9) | 170(106 to 257) | 3.3(2 to 5.1) | 6.2(-23.9 to 44.8) |

| | | | | | |
|---|---|---|---|---|---|
| Finland | 161(98 to 242) | 3.4(2 to 5.1) | 155(96 to 234) | 3.2(1.9 to 4.9) | -5.3(-32.7 to 34.2) |
| France | 1649(940 to 2595) | 3(1.7 to 4.6) | 1768(1077 to 2732) | 3.1(1.9 to 4.9) | 5.2(-25.7 to 48.2) |
| Germany | 2609(1603 to 3993) | 3.5(2.1 to 5.5) | 3100(1963 to 4557) | 4.6(2.8 to 6.9) | 30.5(-4.7 to 80.5) |
| Greece | 289(174 to 449) | 2.9(1.7 to 4.5) | 254(149 to 391) | 3.1(1.7 to 4.9) | 5.7(-23.1 to 53.2) |
| Iceland | 8(5 to 13) | 3.2(1.9 to 5) | 12(7 to 17) | 3.7(2.1 to 5.7) | 13.5(-18.6 to 55.9) |
| Ireland | 108(62 to 172) | 2.9(1.7 to 4.7) | 136(81 to 208) | 3.1(1.8 to 4.8) | 4.6(-26.4 to 46.8) |
| Israel | 144(85 to 219) | 2.8(1.7 to 4.4) | 265(161 to 418) | 3(1.8 to 4.8) | 6.1(-24.4 to 48.6) |
| Italy | 1366(880 to 2030) | 2.5(1.6 to 3.7) | 1130(744 to 1638) | 2.6(1.7 to 3.9) | 7.3(-8 to 26.4) |
| Luxembourg | 13(8 to 21) | 3.9(2.3 to 6) | 21(13 to 30) | 3.9(2.5 to 5.7) | -0.4(-28.8 to 43.1) |
| Malta | 9(5 to 14) | 2.5(1.5 to 3.9) | 10(6 to 15) | 2.9(1.8 to 4.4) | 15.3(-21.4 to 70.9) |
| Monaco | 1(0 to 1) | 2.9(1.7 to 4.6) | 1(1 to 1) | 3(1.7 to 4.7) | 3.3(-25.9 to 44.7) |
| Netherlands | 458(271 to 701) | 3.1(1.8 to 4.8) | 472(287 to 709) | 3.2(1.9 to 4.9) | 2.6(-26.7 to 45.4) |
| Norway | 136(87 to 203) | 3.3(2.1 to 5) | 178(113 to 257) | 3.7(2.3 to 5.3) | 10.4(-1.2 to 25.4) |
| San Marino | 1(0 to 1) | 3(1.8 to 4.6) | 1(1 to 1) | 3.1(1.8 to 4.9) | 5(-25.1 to 42.2) |
| Portugal | 180(102 to 285) | 1.8(1 to 2.9) | 179(112 to 262) | 2.1(1.2 to 3.2) | 18.1(-19.8 to 77.1) |
| Spain | 1140(675 to 1775) | 2.9(1.7 to 4.6) | 1210(751 to 1825) | 3.2(1.9 to 5) | 7.8(-24.2 to 54.1) |
| Sweden | 298(188 to 438) | 3.8(2.3 to 5.7) | 368(225 to 549) | 4.3(2.6 to 6.5) | 10.9(-15.6 to 41.6) |
| Switzerland | 244(154 to 364) | 3.8(2.4 to 5.7) | 291(184 to 430) | 3.9(2.4 to 5.7) | 2.4(-26.4 to 42.8) |
| United Kingdom | 1267(817 to 1858) | 2.4(1.5 to 3.5) | 1484(979 to 2135) | 2.5(1.6 to 3.6) | 5.6(-1.8 to 14.2) |
| Southern Latin America | 1238(763 to 1875) | 2.5(1.5 to 3.7) | 2394(1497 to 3585) | 3.6(2.2 to 5.4) | 46.6(14.1 to 86.4) |
| Argentina | 807(485 to 1229) | 2.4(1.5 to 3.7) | 1559(925 to 2449) | 3.5(2 to 5.4) | 41.8(-0.1 to 98.2) |
| Chile | 354(209 to 576) | 2.5(1.5 to 4) | 721(437 to 1053) | 4.1(2.4 to 6) | 60.4(15.2 to 126.5) |
| Uruguay | 77(46 to 121) | 2.5(1.5 to 3.9) | 115(70 to 175) | 3.5(2.1 to 5.3) | 38.4(-0.5 to 89.7) |
| Eastern Europe | 5810(3758 to 8680) | 2.7(1.7 to 4.1) | 5795(3710 to 8441) | 3.3(2.1 to 4.9) | 19.7(9.4 to 31.6) |
| Belarus | 371(216 to 576) | 3.7(2.1 to 5.8) | 312(194 to 484) | 3.8(2.3 to 6) | 2.8(-24.7 to 39.9) |

| | | | | | |
|---|---|---|---|---|---|
| Estonia | 55(33 to 82) | 3.7(2.2 to 5.6) | 42(25 to 61) | 3.8(2.2 to 5.8) | 2.1(-25.8 to 39.7) |
| Latvia | 97(60 to 146) | 3.9(2.3 to 5.9) | 64(39 to 95) | 4.1(2.5 to 6.4) | 6.3(-23.6 to 49.1) |
| Lithuania | 138(83 to 211) | 3.9(2.3 to 5.9) | 95(60 to 142) | 4.1(2.5 to 6.2) | 5.5(-21.5 to 41.5) |
| Republic of Moldova | 165(99 to 250) | 3.8(2.3 to 5.7) | 122(75 to 185) | 3.7(2.2 to 5.7) | -1.6(-27.1 to 29.5) |
| Russian Federation | 3490(2235 to 5188) | 2.5(1.6 to 3.7) | 3941(2522 to 5770) | 3.2(2 to 4.8) | 29.5(20.9 to 39.3) |
| Ukraine | 1493(894 to 2319) | 3.1(1.8 to 4.8) | 1221(731 to 1840) | 3.3(2 to 5.1) | 7.8(-21.3 to 46) |
| Central Europe | 2950(1887 to 4352) | 2.5(1.6 to 3.7) | 2486(1625 to 3576) | 2.7(1.7 to 3.9) | 7.4(-3.7 to 20.2) |
| Albania | 90(52 to 143) | 2.5(1.5 to 3.9) | 64(38 to 99) | 2.6(1.5 to 4.1) | 6(-24.2 to 48.7) |
| Bosnia and Herzegovina | 116(68 to 177) | 2.5(1.4 to 3.8) | 70(42 to 105) | 2.5(1.5 to 3.9) | 0.6(-24.1 to 35.5) |
| Bulgaria | 209(126 to 323) | 2.6(1.5 to 4.1) | 153(97 to 230) | 2.7(1.6 to 4.3) | 4.4(-23.8 to 45.9) |
| Croatia | 340(200 to 534) | 2.9(1.7 to 4.5) | 387(239 to 580) | 3.8(2.2 to 5.9) | 30.5(-6.3 to 82.5) |
| Czechia | 172(103 to 272) | 1.3(0.8 to 2) | 482(276 to 772) | 1.7(1 to 2.6) | 28.5(-8.1 to 75.1) |
| Hungary | 261(164 to 403) | 2.6(1.6 to 4.2) | 219(136 to 336) | 2.7(1.6 to 4.3) | 2.7(-25.2 to 39.9) |
| North Macedonia | 51(30 to 81) | 2.5(1.5 to 3.9) | 49(29 to 76) | 2.6(1.4 to 4.2) | 3.5(-25.7 to 46) |
| Montenegro | 16(9 to 25) | 2.5(1.5 to 4) | 14(9 to 22) | 2.6(1.5 to 4.1) | 2.5(-26.2 to 45.4) |
| Poland | 809(516 to 1217) | 2.2(1.4 to 3.3) | 704(463 to 997) | 2.3(1.5 to 3.3) | 2.7(-13.8 to 26.5) |
| Romania | 584(337 to 913) | 2.5(1.5 to 4) | 411(252 to 627) | 2.6(1.5 to 4.1) | 2(-27.9 to 40.6) |
| Serbia | 209(124 to 326) | 2.3(1.4 to 3.6) | 214(131 to 326) | 2.9(1.7 to 4.6) | 25.3(-12.6 to 75.8) |
| Slovakia | 138(84 to 215) | 2.6(1.6 to 4.1) | 141(89 to 208) | 3.2(1.9 to 4.9) | 19.8(-13.1 to 63.5) |
| Slovenia | 53(31 to 81) | 2.8(1.6 to 4.4) | 53(34 to 77) | 3.3(2.1 to 5) | 20.1(-20.6 to 83.2) |
| Central Asia | 2170(1368 to 3309) | 2.9(1.9 to 4.4) | 2799(1767 to 4212) | 2.9(1.8 to 4.4) | 1.3(-13.5 to 17) |
| Armenia | 97(56 to 153) | 2.7(1.6 to 4.3) | 83(50 to 130) | 3.1(1.7 to 5) | 11.7(-23.1 to 57.8) |
| Azerbaijan | 222(125 to 349) | 2.8(1.6 to 4.3) | 292(173 to 455) | 2.9(1.7 to 4.5) | 3.9(-27.6 to 46.3) |
| Georgia | 141(82 to 229) | 2.6(1.5 to 4.3) | 91(55 to 138) | 2.9(1.7 to 4.5) | 11.4(-21.3 to 60) |
| Kazakhstan | 516(304 to 807) | 3(1.8 to 4.7) | 530(319 to 833) | 3(1.7 to 4.7) | -0.3(-29.3 to 39.2) |

| | | | | | |
|---|---|---|---|---|---|
| Kyrgyzstan | 129(75 to 206) | 2.7(1.6 to 4.2) | 200(119 to 310) | 2.9(1.8 to 4.5) | 9.9(-20.8 to 55) |
| Mongolia | 172(107 to 258) | 6.6(4.2 to 9.8) | 131(79 to 201) | 4(2.4 to 6.1) | -39.8(-55.4 to -18.6) |
| Tajikistan | 160(91 to 257) | 2.7(1.6 to 4.3) | 291(169 to 467) | 2.8(1.7 to 4.5) | 4.3(-24.9 to 46.4) |
| Turkmenistan | 112(65 to 180) | 2.7(1.6 to 4.3) | 151(93 to 233) | 2.9(1.8 to 4.5) | 7.3(-23.3 to 49.4) |
| Uzbekistan | 622(364 to 985) | 2.7(1.6 to 4.2) | 1030(600 to 1610) | 2.9(1.7 to 4.4) | 6.8(-24 to 50.5) |
| Central Latin America | 7125(4500 to 10503) | 3.7(2.4 to 5.4) | 10795(6781 to 16177) | 4.3(2.7 to 6.4) | 16.3(6.1 to 27.3) |
| Colombia | 1585(919 to 2459) | 4.4(2.6 to 6.7) | 2380(1465 to 3631) | 5.2(3.1 to 8) | 18(-12 to 55.5) |
| Costa Rica | 164(98 to 264) | 4.9(3 to 7.6) | 230(138 to 355) | 5.1(3 to 7.9) | 3.4(-23.7 to 38.2) |
| El Salvador | 373(234 to 556) | 5.8(3.7 to 8.6) | 302(182 to 458) | 4.7(2.8 to 7.1) | -19.5(-42.4 to 10.3) |
| Guatemala | 701(442 to 1026) | 7.6(4.8 to 10.9) | 1021(603 to 1536) | 5.1(3.1 to 7.6) | -32.6(-50.5 to -10.9) |
| Honduras | 298(180 to 470) | 5.4(3.4 to 8.3) | 591(365 to 891) | 5.4(3.3 to 8.1) | -0.2(-27.6 to 33.8) |
| Mexico | 2626(1591 to 4003) | 2.5(1.5 to 3.7) | 4350(2729 to 6414) | 3.5(2.2 to 5.1) | 39.9(28.8 to 53.7) |
| Nicaragua | 203(120 to 316) | 4.4(2.7 to 6.7) | 344(205 to 531) | 4.9(2.9 to 7.5) | 11.3(-19.7 to 49.1) |
| Panama | 136(79 to 210) | 5.1(3 to 7.8) | 210(123 to 323) | 5(3 to 7.8) | -0.7(-26 to 32.1) |
| Venezuela (Bolivarian Republic of) | 1038(612 to 1584) | 4.9(3 to 7.3) | 1369(817 to 2145) | 5(3 to 7.8) | 2.3(-24.2 to 37.8) |
| Andean Latin America | 6399(4300 to 9245) | 14.4(9.7 to 20.7) | 6569(4287 to 9596) | 10(6.5 to 14.5) | -30.7(-41.2 to -17.9) |
| Bolivia (Plurinational State of) | 760(484 to 1118) | 10.4(6.6 to 15.1) | 1151(712 to 1700) | 9.2(5.7 to 13.5) | -11.1(-30.4 to 13.6) |
| Ecuador | 907(581 to 1312) | 8.1(5.2 to 11.7) | 1832(1200 to 2643) | 10(6.5 to 14.3) | 23(-2.7 to 57.4) |
| Peru | 4732(3164 to 6831) | 18.6(12.4 to 26.7) | 3586(2283 to 5416) | 10.4(6.6 to 15.6) | -44.3(-56.6 to -29) |
| Caribbean | 1002(636 to 1522) | 2.6(1.7 to 3.9) | 1574(1023 to 2353) | 3.4(2.2 to 5) | 28.3(9.7 to 50.4) |
| Antigua and Barbuda | 2(1 to 3) | 2.8(1.6 to 4.3) | 3(2 to 5) | 3.7(2.1 to 5.7) | 35(-3.9 to 86.5) |
| Bahamas | 8(4 to 12) | 2.6(1.5 to 4.1) | 14(8 to 20) | 3.5(2.1 to 5.3) | 34.8(0.9 to 86.6) |
| Barbados | 7(4 to 11) | 2.7(1.5 to 4.3) | 10(6 to 15) | 3.6(2.2 to 5.6) | 34.3(-4 to 88.8) |
| Belize | 5(3 to 8) | 2.4(1.4 to 3.8) | 15(9 to 24) | 3.3(1.9 to 5) | 35(-5.6 to 88.3) |

| | | | | | |
|---|---|---|---|---|---|
| Bermuda | 2(1 to 3) | 2.7(1.6 to 4.4) | 2(1 to 3) | 3.9(2.4 to 5.9) | 41(2.2 to 95.6) |
| Cuba | 18(10 to 28) | 2.3(1.3 to 3.6) | 28(18 to 42) | 2.5(1.5 to 3.8) | 8.7(-25.2 to 61) |
| Dominica | 2(1 to 3) | 2.6(1.5 to 4) | 2(1 to 4) | 3.2(1.9 to 5.3) | 24.2(-12.2 to 74.4) |
| Dominican Republic | 222(127 to 355) | 2.7(1.6 to 4.2) | 407(247 to 657) | 3.6(2.2 to 5.8) | 35.2(-4.4 to 96.4) |
| Grenada | 2(1 to 4) | 2.5(1.4 to 4) | 4(2 to 6) | 3.4(2.1 to 5.4) | 39.3(1.3 to 96.7) |
| Guyana | 23(14 to 35) | 2.6(1.6 to 4) | 29(17 to 45) | 3.5(2.1 to 5.4) | 33.7(-3.5 to 85.6) |
| Haiti | 130(75 to 209) | 1.9(1.1 to 2.9) | 356(205 to 560) | 2.6(1.5 to 4) | 38.7(-4.9 to 103.8) |
| Jamaica | 70(40 to 110) | 2.7(1.6 to 4.2) | 102(61 to 158) | 3.5(2.1 to 5.4) | 31(-6 to 87.7) |
| Puerto Rico | 102(60 to 162) | 2.7(1.6 to 4.3) | 112(66 to 170) | 3.7(2.1 to 5.7) | 35.2(-1.2 to 88.1) |
| Saint Kitts and Nevis | 1(1 to 2) | 3(1.7 to 4.6) | 2(1 to 3) | 3.9(2.3 to 6) | 31.1(-6 to 88.4) |
| Saint Lucia | 4(2 to 6) | 2.5(1.5 to 4) | 6(4 to 10) | 3.6(2.1 to 5.8) | 41(-0.1 to 96.7) |
| Saint Vincent and the Grenadines | 3(2 to 5) | 2.5(1.5 to 4) | 4(2 to 6) | 3.4(2 to 5.4) | 33.7(-5.3 to 91.6) |
| Suriname | 10(6 to 17) | 2.4(1.4 to 3.8) | 19(11 to 29) | 3.3(1.9 to 5.2) | 37.3(-2.9 to 92.3) |
| Trinidad and Tobago | 33(19 to 51) | 2.5(1.5 to 3.9) | 44(27 to 68) | 3.4(2 to 5.3) | 36.5(-4.4 to 91.5) |
| United States Virgin Islands | 3(2 to 5) | 3.1(1.9 to 4.7) | 3(2 to 5) | 3.9(2.3 to 5.9) | 26.1(-7.4 to 77.6) |
| Tropical Latin America | 2176(1371 to 3220) | 1.3(0.8 to 1.9) | 3616(2379 to 5269) | 1.6(1.1 to 2.4) | 27.8(17.7 to 39.9) |
| Brazil | 2019(1283 to 3000) | 1.2(0.8 to 1.8) | 3416(2241 to 4983) | 1.6(1 to 2.3) | 32.1(21.5 to 45) |
| Paraguay | 156(92 to 243) | 3.9(2.4 to 5.9) | 200(118 to 305) | 2.8(1.7 to 4.2) | -28.9(-47.8 to -2.7) |
| East Asia | 26374(16631 to 39142) | 2(1.3 to 2.9) | 31567(20685 to 45211) | 2.2(1.4 to 3.1) | 9.1(1.5 to 18.2) |
| China | 25637(16171 to 38094) | 2(1.3 to 2.9) | 30593(20038 to 43872) | 2.2(1.4 to 3.1) | 9.3(1.5 to 18.8) |
| Democratic People's Republic of Korea | 432(278 to 626) | 2.1(1.4 to 3) | 545(355 to 787) | 2(1.3 to 2.9) | -3.7(-8.8 to 1.9) |
| Taiwan (Province of China) | 305(194 to 444) | 1.5(0.9 to 2.1) | 430(285 to 608) | 1.7(1.1 to 2.5) | 17.6(7.6 to 31.3) |
| Southeast Asia | 7611(4716 to 11322) | 1.5(0.9 to 2.2) | 13780(8689 to 20227) | 2(1.3 to 2.9) | 34.5(20.4 to 51.4) |

| | | | | | |
|---|---|---|---|---|---|
| Cambodia | 301(179 to 469) | 2.7(1.7 to 4.2) | 554(323 to 867) | 3.2(1.9 to 5) | 16.7(-15.9 to 64.2) |
| Indonesia | 1174(723 to 1772) | 0.6(0.3 to 0.8) | 2948(1833 to 4410) | 1.1(0.7 to 1.6) | 97.7(91.9 to 104.9) |
| Lao People's Democratic Republic | 131(75 to 204) | 2.9(1.7 to 4.4) | 251(146 to 387) | 3.2(1.9 to 4.9) | 12.5(-16.7 to 56.3) |
| Malaysia | 420(232 to 665) | 2.3(1.3 to 3.5) | 952(565 to 1490) | 2.9(1.8 to 4.5) | 28.3(-8 to 80.1) |
| Maldives | 4(3 to 7) | 1.9(1.2 to 2.9) | 14(8 to 22) | 2.7(1.6 to 4.3) | 40.1(5.5 to 86.7) |
| Mauritius | 24(15 to 39) | 2(1.2 to 3.2) | 33(20 to 52) | 2.6(1.6 to 4.1) | 28.8(-5.5 to 75.3) |
| Myanmar | 1180(700 to 1871) | 2.6(1.6 to 4.1) | 1677(1008 to 2549) | 3(1.8 to 4.5) | 13.1(-18.4 to 60.5) |
| Philippines | 505(312 to 760) | 0.7(0.4 to 1) | 1511(940 to 2271) | 1.2(0.8 to 1.9) | 78.4(70.9 to 86.6) |
| Sri Lanka | 375(226 to 588) | 2(1.2 to 3.1) | 583(343 to 878) | 2.7(1.6 to 4) | 32.3(-1.7 to 77.5) |
| Seychelles | 2(1 to 3) | 2.4(1.5 to 3.7) | 3(2 to 5) | 3.1(1.8 to 4.7) | 28.3(-2.2 to 70.1) |
| Thailand | 1741(1012 to 2745) | 2.7(1.6 to 4.2) | 1982(1195 to 2935) | 3(1.7 to 4.5) | 8.4(-25 to 51.5) |
| Timor-Leste | 20(12 to 32) | 2.4(1.5 to 3.8) | 46(26 to 75) | 3.2(1.9 to 4.9) | 30.4(-8.9 to 82) |
| Viet Nam | 1723(976 to 2762) | 2.4(1.4 to 3.7) | 3209(1946 to 4831) | 3.3(2 to 5) | 40.1(1.9 to 97.2) |
| Oceania | 78(49 to 118) | 1.2(0.7 to 1.7) | 187(118 to 279) | 1.3(0.9 to 2) | 16.2(12.2 to 21.1) |
| American Samoa | 1(0 to 1) | 1.3(0.8 to 1.9) | 1(1 to 1) | 1.5(0.9 to 2.1) | 15.9(9.9 to 22.6) |
| Cook Islands | 0(0 to 0) | 1.2(0.8 to 1.8) | 0(0 to 0) | 1.4(0.9 to 2.1) | 17.4(10.6 to 24.9) |
| Micronesia (Federated States of) | 1(1 to 2) | 1.3(0.8 to 1.9) | 2(1 to 2) | 1.5(0.9 to 2.2) | 16.5(10 to 23.2) |
| Fiji | 10(6 to 15) | 1.3(0.8 to 1.9) | 14(9 to 20) | 1.5(0.9 to 2.2) | 19.1(12.5 to 25.5) |
| Guam | 2(1 to 3) | 1.2(0.8 to 1.8) | 2(1 to 4) | 1.4(0.9 to 2.1) | 17.1(10.4 to 23.8) |
| Kiribati | 1(1 to 1) | 1.3(0.8 to 1.9) | 2(1 to 2) | 1.3(0.8 to 1.9) | 3.4(-2 to 8.7) |
| Nauru | 0(0 to 0) | 1.3(0.8 to 1.9) | 0(0 to 0) | 1.6(1 to 2.3) | 18(12.4 to 24.4) |
| Niue | 0(0 to 0) | 1.3(0.8 to 1.8) | 0(0 to 0) | 1.5(1 to 2.2) | 20.9(14.7 to 28.8) |
| Marshall Islands | 1(0 to 1) | 1.3(0.8 to 1.9) | 1(1 to 1) | 1.4(0.9 to 2.1) | 8.8(2.5 to 15.1) |
| Northern Mariana Islands | 1(0 to 1) | 1.2(0.8 to 1.8) | 1(0 to 1) | 1.5(0.9 to 2.1) | 18.7(11.3 to 25.4) |

| | | | | | |
|---|---|---|---|---|---|
| Palau | 0(0 to 0) | 1.3(0.8 to 1.9) | 0(0 to 0) | 1.5(1 to 2.2) | 21.1(14.7 to 28) |
| Papua New Guinea | 47(29 to 71) | 1.1(0.7 to 1.6) | 136(85 to 204) | 1.3(0.8 to 1.9) | 18.9(13 to 25.7) |
| Samoa | 2(1 to 3) | 1.2(0.8 to 1.8) | 3(2 to 5) | 1.4(0.9 to 2.1) | 18.4(11.7 to 24.8) |
| Solomon Islands | 4(3 to 7) | 1.3(0.8 to 1.9) | 10(6 to 15) | 1.5(1 to 2.2) | 12.4(5.8 to 17.9) |
| Tokelau | 0(0 to 0) | 1.2(0.7 to 1.7) | 0(0 to 0) | 1.4(0.9 to 2.1) | 24.6(17.3 to 32.5) |
| Tonga | 1(1 to 2) | 1.2(0.8 to 1.8) | 1(1 to 2) | 1.5(0.9 to 2.1) | 16.5(9.8 to 22.8) |
| Tuvalu | 0(0 to 0) | 1.2(0.8 to 1.7) | 0(0 to 0) | 1.5(0.9 to 2.1) | 22.8(15.2 to 30.6) |
| Vanuatu | 2(1 to 3) | 1.4(0.9 to 2.1) | 4(3 to 7) | 1.5(0.9 to 2.2) | 3(-14 to 19.2) |
| North Africa and Middle East | 8245(5161 to 12538) | 2.2(1.4 to 3.3) | 20714(12951 to 30887) | 3.2(2 to 4.8) | 46.5(32.5 to 63) |
| Afghanistan | 303(175 to 484) | 2.6(1.5 to 3.9) | 1210(691 to 1915) | 2.9(1.7 to 4.4) | 11.8(-21 to 59.6) |
| Algeria | 696(393 to 1124) | 2.5(1.5 to 3.9) | 1446(867 to 2264) | 3.4(2.1 to 5.3) | 35.9(-1.4 to 90.8) |
| Bahrain | 13(8 to 21) | 2.4(1.4 to 3.7) | 50(29 to 77) | 3.4(2.1 to 5.4) | 44.1(3.8 to 103.3) |
| Egypt | 1282(738 to 2130) | 2.2(1.3 to 3.5) | 3335(1925 to 5267) | 3.2(1.9 to 4.9) | 47.4(1.5 to 109.1) |
| Iran (Islamic Republic of) | 1167(733 to 1769) | 1.8(1.1 to 2.7) | 2532(1587 to 3744) | 2.9(1.8 to 4.3) | 64.4(50.5 to 79.5) |
| Iraq | 412(228 to 671) | 2.2(1.3 to 3.5) | 1555(917 to 2453) | 3.3(2 to 5.2) | 52.2(5.4 to 118.6) |
| Jordan | 98(55 to 160) | 2.3(1.4 to 3.7) | 436(253 to 692) | 3.4(2 to 5.4) | 49(6.2 to 108.9) |
| Kuwait | 48(27 to 75) | 2.4(1.4 to 3.8) | 162(97 to 255) | 3.5(2.1 to 5.5) | 42.6(1.6 to 96.1) |
| Lebanon | 77(45 to 127) | 2.3(1.4 to 3.8) | 179(105 to 277) | 3.5(2.1 to 5.5) | 51.7(7.5 to 115.5) |
| Libya | 109(60 to 179) | 2.4(1.3 to 3.8) | 244(145 to 387) | 3.3(2 to 5.3) | 40.6(-0.1 to 101.3) |
| Morocco | 659(374 to 1043) | 2.3(1.4 to 3.6) | 1200(714 to 1916) | 3.2(1.9 to 5.1) | 36.9(-5.7 to 88.1) |
| Palestine | 54(30 to 92) | 2.5(1.5 to 4.1) | 185(108 to 293) | 3.4(2.1 to 5.3) | 36.3(-3.9 to 95.9) |
| Oman | 48(27 to 77) | 2.3(1.3 to 3.6) | 177(99 to 282) | 3.4(2 to 5.3) | 49.6(2.9 to 118) |
| Qatar | 11(7 to 18) | 2.3(1.3 to 3.6) | 111(58 to 182) | 3.4(1.9 to 5.3) | 49.9(2.4 to 113.2) |
| Saudi Arabia | 394(225 to 642) | 2.2(1.3 to 3.5) | 1351(791 to 2134) | 3.4(2 to 5.3) | 52.3(8.3 to 119.9) |
| Sudan | 483(277 to 781) | 2.2(1.3 to 3.5) | 1429(820 to 2257) | 3.2(1.9 to 4.9) | 42(1 to 101.4) |

| | | | | | |
|---|---|---|---|---|---|
| Syrian Arab Republic | 361(199 to 581) | 2.5(1.5 to 3.9) | 537(307 to 845) | 3.4(2 to 5.3) | 35.9(-4 to 88.8) |
| Tunisia | 218(123 to 357) | 2.4(1.4 to 3.8) | 385(231 to 604) | 3.4(2 to 5.3) | 41.4(1.2 to 93.9) |
| Turkey | 1415(802 to 2247) | 2.2(1.3 to 3.3) | 2801(1642 to 4353) | 3.3(1.9 to 5.2) | 53.9(7.4 to 111.7) |
| United Arab Emirates | 47(27 to 75) | 2.3(1.3 to 3.6) | 311(172 to 501) | 3.3(1.9 to 5.1) | 44.5(-0.6 to 106.8) |
| Yemen | 343(194 to 548) | 2.4(1.4 to 3.7) | 1056(595 to 1674) | 3.1(1.8 to 4.8) | 27.1(-9.6 to 76.8) |
| South Asia | 23958(15558 to 35683) | 2(1.3 to 3) | 60854(38899 to 89567) | 3.1(2 to 4.5) | 52.5(36.3 to 70.7) |
| Bangladesh | 12622(7937 to 18898) | 11(7 to 16.2) | 26722(17201 to 40128) | 15.7(10.1 to 23.5) | 42.5(16.1 to 76) |
| Bhutan | 70(46 to 101) | 10.3(6.8 to 14.7) | 114(72 to 172) | 13.6(8.8 to 20.4) | 31.5(7.3 to 60.4) |
| India | 8028(5127 to 12010) | 0.9(0.6 to 1.3) | 26526(16790 to 39330) | 1.7(1.1 to 2.6) | 101.4(88.2 to 113.6) |
| Nepal | 2116(1358 to 3112) | 10.8(7 to 15.7) | 3004(1891 to 4369) | 9.1(5.7 to 13.1) | -15.4(-32.4 to 4.4) |
| Pakistan | 1121(690 to 1701) | 0.9(0.6 to 1.4) | 4488(2768 to 6996) | 1.7(1.1 to 2.6) | 86.1(51.8 to 123.1) |
| Southern Sub-Saharan Africa | 1053(647 to 1620) | 1.7(1.1 to 2.6) | 1837(1152 to 2840) | 2.2(1.4 to 3.3) | 25.1(10 to 43.7) |
| Botswana | 49(28 to 78) | 3.4(2 to 5.3) | 95(55 to 149) | 3.7(2.2 to 5.9) | 10.3(-21.8 to 57.8) |
| Lesotho | 63(38 to 99) | 3.2(2 to 4.8) | 74(43 to 118) | 3.1(1.9 to 5) | -1.4(-29.8 to 39.4) |
| Namibia | 46(26 to 74) | 3(1.7 to 4.6) | 89(51 to 140) | 3.4(2 to 5.3) | 13.9(-18.6 to 61.1) |
| South Africa | 540(329 to 819) | 1.3(0.8 to 1.9) | 1041(645 to 1583) | 1.8(1.1 to 2.7) | 41.3(24.5 to 59.9) |
| Eswatini | 27(16 to 44) | 3(1.8 to 4.7) | 42(24 to 67) | 3.2(2 to 5.1) | 7.6(-23.7 to 54.1) |
| Zimbabwe | 327(173 to 523) | 2.8(1.6 to 4.4) | 497(282 to 784) | 3(1.7 to 4.6) | 4.4(-25.3 to 49.7) |
| Western Sub-Saharan Africa | 2002(1241 to 3044) | 1(0.6 to 1.4) | 7249(4465 to 11015) | 1.4(0.9 to 2.2) | 47.9(37 to 58.6) |
| Benin | 66(39 to 105) | 1.3(0.8 to 2.1) | 237(127 to 379) | 1.7(1 to 2.7) | 30(-8 to 79.2) |
| Burkina Faso | 130(75 to 209) | 1.3(0.8 to 2.1) | 414(227 to 672) | 1.7(1 to 2.7) | 29.9(-8.6 to 79.3) |
| Cameroon | 146(86 to 238) | 1.3(0.8 to 2.1) | 574(328 to 905) | 1.7(1 to 2.7) | 31.4(-6.4 to 80) |
| Cabo Verde | 5(3 to 9) | 1.4(0.8 to 2.2) | 12(7 to 19) | 1.9(1.1 to 3.1) | 41.3(2 to 93) |
| Chad | 86(49 to 139) | 1.4(0.8 to 2.2) | 290(155 to 479) | 1.6(0.9 to 2.6) | 19(-13.5 to 61.7) |
| Cote d'Ivoire | 153(98 to 231) | 3.4(2.1 to 5.2) | 113(72 to 164) | 3.4(2.1 to 5.1) | 0(-29.7 to 44.1) |

| | | | | | |
|---|---|---|---|---|---|
| Gambia | 15(9 to 25) | 1.4(0.9 to 2.2) | 46(26 to 76) | 1.8(1.1 to 2.8) | 27(-7.5 to 70.3) |
| Ghana | 239(141 to 375) | 1.5(0.9 to 2.3) | 658(385 to 1061) | 1.9(1.1 to 3) | 25.9(-8.9 to 71.1) |
| Guinea | 83(49 to 131) | 1.3(0.8 to 2) | 234(132 to 370) | 1.7(1 to 2.6) | 29.3(-7.6 to 79.2) |
| Guinea-Bissau | 15(9 to 24) | 1.4(0.8 to 2.1) | 37(21 to 59) | 1.7(1 to 2.7) | 22.6(-13.8 to 68.4) |
| Liberia | 26(15 to 40) | 1.3(0.8 to 2) | 98(53 to 155) | 1.8(1 to 2.8) | 40.5(-1.5 to 96.6) |
| Mali | 119(70 to 192) | 1.3(0.8 to 2.1) | 418(233 to 681) | 1.7(1 to 2.8) | 30.8(-5.9 to 79.5) |
| Mauritania | 29(17 to 46) | 1.3(0.8 to 2) | 83(45 to 139) | 1.9(1.1 to 3) | 40.3(-1.9 to 98) |
| Niger | 117(71 to 179) | 1.4(0.9 to 2.1) | 424(234 to 696) | 1.7(1 to 2.7) | 23(-10.7 to 69.4) |
| Nigeria | 534(327 to 806) | 0.5(0.3 to 0.8) | 2627(1598 to 3991) | 1.1(0.7 to 1.7) | 101.1(93.9 to 109.8) |
| Sao Tome and Principe | 2(1 to 3) | 1.3(0.8 to 2) | 4(2 to 7) | 1.8(1 to 2.8) | 34.7(-3.6 to 86.1) |
| Senegal | 113(68 to 179) | 1.4(0.9 to 2.2) | 292(167 to 460) | 1.7(1 to 2.6) | 24.6(-11.9 to 69) |
| Sierra Leone | 50(30 to 79) | 1.3(0.8 to 2) | 170(94 to 273) | 1.8(1 to 2.8) | 37(0.8 to 91.8) |
| Togo | 54(32 to 86) | 1.4(0.8 to 2.1) | 150(85 to 235) | 1.7(1 to 2.7) | 28.4(-8.3 to 77.6) |
| Eastern Sub-Saharan Africa | 2123(1300 to 3234) | 1(0.6 to 1.6) | 6956(4286 to 10506) | 1.5(0.9 to 2.2) | 45.5(31.2 to 61.2) |
| Burundi | 80(47 to 125) | 1.4(0.8 to 2.1) | 250(135 to 389) | 2(1.1 to 3) | 42.8(3.2 to 94) |
| Comoros | 8(5 to 13) | 1.6(1 to 2.5) | 16(9 to 27) | 2.1(1.2 to 3.4) | 30.8(-7.6 to 80.8) |
| Djibouti | 9(5 to 14) | 1.6(0.9 to 2.5) | 27(15 to 43) | 2.1(1.2 to 3.4) | 32.2(-6.7 to 85.3) |
| Eritrea | 45(27 to 71) | 1.4(0.8 to 2.1) | 153(86 to 249) | 2(1.2 to 3.2) | 48(4.8 to 101.1) |
| Ethiopia | 106(65 to 162) | 0.2(0.1 to 0.3) | 783(468 to 1219) | 0.6(0.4 to 0.9) | 207.4(178.6 to 243.7) |
| Kenya | 133(79 to 208) | 0.5(0.3 to 0.7) | 460(276 to 710) | 0.8(0.5 to 1.1) | 58.5(53.9 to 63.8) |
| Madagascar | 207(114 to 357) | 1.6(0.9 to 2.6) | 583(336 to 942) | 2(1.2 to 3.1) | 24.7(-13.9 to 80.1) |
| Malawi | 156(90 to 244) | 1.5(0.9 to 2.3) | 434(236 to 700) | 2.1(1.2 to 3.1) | 36.6(0.5 to 86.1) |
| Mozambique | 220(128 to 345) | 1.6(1 to 2.5) | 739(423 to 1173) | 2.4(1.4 to 3.6) | 47.8(6.9 to 100.2) |
| Rwanda | 123(73 to 196) | 1.6(1 to 2.5) | 310(176 to 498) | 2.2(1.3 to 3.4) | 36.9(-3 to 85.9) |
| Somalia | 117(70 to 189) | 1.5(1 to 2.4) | 424(229 to 690) | 1.9(1.1 to 2.9) | 22.1(-13.8 to 67.8) |

| | | | | | |
|---|---|---|---|---|---|
| South Sudan | 90(53 to 146) | 1.4(0.8 to 2.2) | 183(105 to 295) | 1.8(1.1 to 2.8) | 29.2(-3.5 to 69.4) |
| United Republic of Tanzania | 418(240 to 667) | 1.5(0.9 to 2.3) | 1249(680 to 2073) | 2(1.1 to 3.3) | 36.7(-3 to 90) |
| Uganda | 274(159 to 440) | 1.5(0.9 to 2.3) | 922(502 to 1498) | 2(1.2 to 3.2) | 35.6(-6.1 to 86.2) |
| Zambia | 134(78 to 206) | 1.5(0.9 to 2.3) | 418(230 to 681) | 2.1(1.2 to 3.3) | 35.8(-5.3 to 92.5) |
| Central Sub-Saharan Africa | 1479(906 to 2276) | 2.5(1.5 to 3.7) | 4900(3015 to 7598) | 3.4(2.1 to 5.1) | 36(8 to 72.8) |
| Angola | 263(152 to 410) | 2.3(1.4 to 3.5) | 1082(612 to 1706) | 3.3(2 to 5.1) | 41.4(-1.4 to 93.6) |
| Central African Republic | 78(48 to 122) | 2.7(1.7 to 4.2) | 168(98 to 257) | 2.9(1.8 to 4.4) | 5.9(-26.3 to 48.8) |
| Congo | 60(33 to 95) | 2.2(1.3 to 3.4) | 179(105 to 278) | 3.1(1.9 to 4.8) | 40.3(-2.1 to 109.3) |
| Democratic Republic of the Congo | 1044(609 to 1662) | 2.5(1.5 to 3.9) | 3354(1988 to 5210) | 3.4(2.1 to 5.2) | 36.3(-0.5 to 89.9) |
| Equatorial Guinea | 10(6 to 15) | 2.2(1.3 to 3.4) | 54(30 to 87) | 3.2(1.9 to 5.1) | 46.3(1.8 to 107) |
| Gabon | 23(13 to 38) | 2.2(1.3 to 3.5) | 61(36 to 95) | 3.2(1.9 to 4.9) | 44.8(3.2 to 101.2) |
