# Supplementary material for: The global, regional, and national burden of appendicitis in 204 countries and territories, 1990–2019: a systematic analysis from the Global Burden of Disease Study 2019
Source: BMC Gastroenterol. 2023 Feb 22;23:44. doi: 10.1186/s12876-023-02678-7 (PMC9945388; doi:10.1186/s12876-023-02678-7)
Supplement: Supplementary file 2 — Additional file 2. The figures of appendicitis burden in 204 countries and territories, 1990–2019: a systematic analysis from the Global Burden of Disease Study 2019. [file 12876_2023_2678_MOESM2_ESM.pdf]

Additional file 2

The figures of appendicitis burden in 204 countries and territories, 1990–2019: a systematic analysis from the Global Burden of Disease Study 2019

Figure S3: The age-standardized YLDs rate of appendicitis in 2019 for 21 GBD regions, by sex..........................…..2

Figure S4: The percentage change in age-standardized point prevalence of appendicitis from 1990 to 2019 for 21 Global Burden of Disease regions by sex…………………………………………………………………………………...3

Figure S5: The percentage change in age-standardized point incidence of appendicitis from 1990 to 2019 for 21 Global Burden of Disease regions by sex…………………………………………………………………………….................................………………...4

Figure S6: The percentage change in age-standardized point YLDs of appendicitis from 1990 to 2019 for 21 Global Burden of Disease regions by sex………………………………………………………………………………...…......…………………………………………..…...……5

Figure S7: Number of prevalent cases of appendicitis from 1990 to 2019 for 21 Global Burden of Disease regions……………………………….………6

Figure S8: Number of incident cases of appendicitis from 1990 to 2019 for 21 Global Burden of Disease regions…..7

Figure S9: Age-standardized YLDs rates of appendicitis per 100 000 population in 2019, by country and territory..8

Figure S10: The percentage change in age-standardized point prevalence of appendicitis from 1990 to 2019 for country and territories.........................9

Figure S11: The percentage change in age-standardized point incidence of appendicitis from 1990 to 2019 for country and territory……..………..10

Figure S12: The percentage change in age-standardized point YLDs of appendicitis from 1990 to 2019 for country and territory……………………..11

Figure S13: Global cases and age-standardized rates of incidence of appendicitis per 100 000 population by age and sex, 2019……….......................12

Figure S14: Global cases and age-standardized rates of YLDs of appendicitis per 100 000 population by age and sex, 2019……………………...……..13

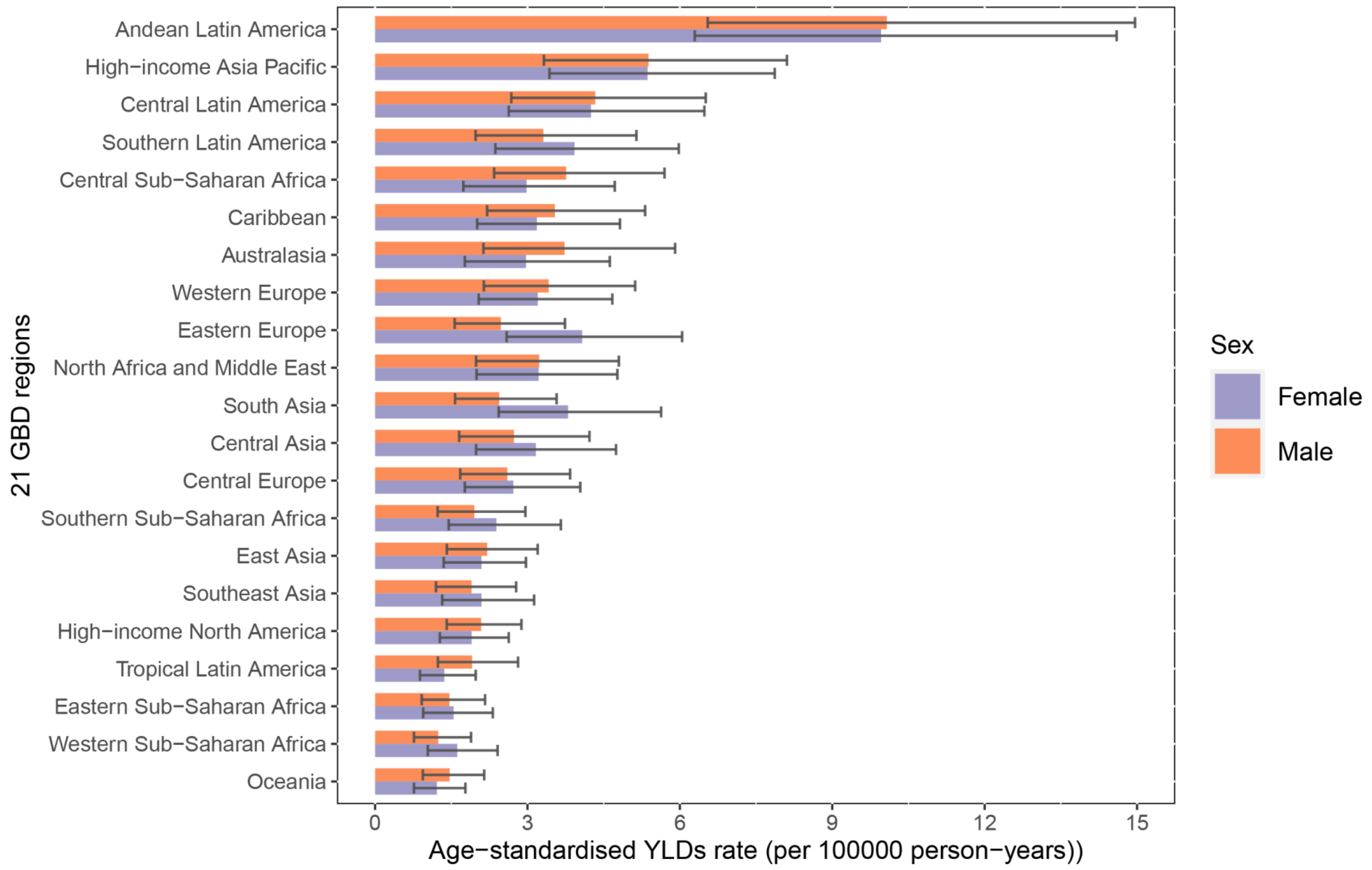

Figure S3: The age-standardized YLDs rate of appendicitis in 2019 for 21 GBD regions, by sex.

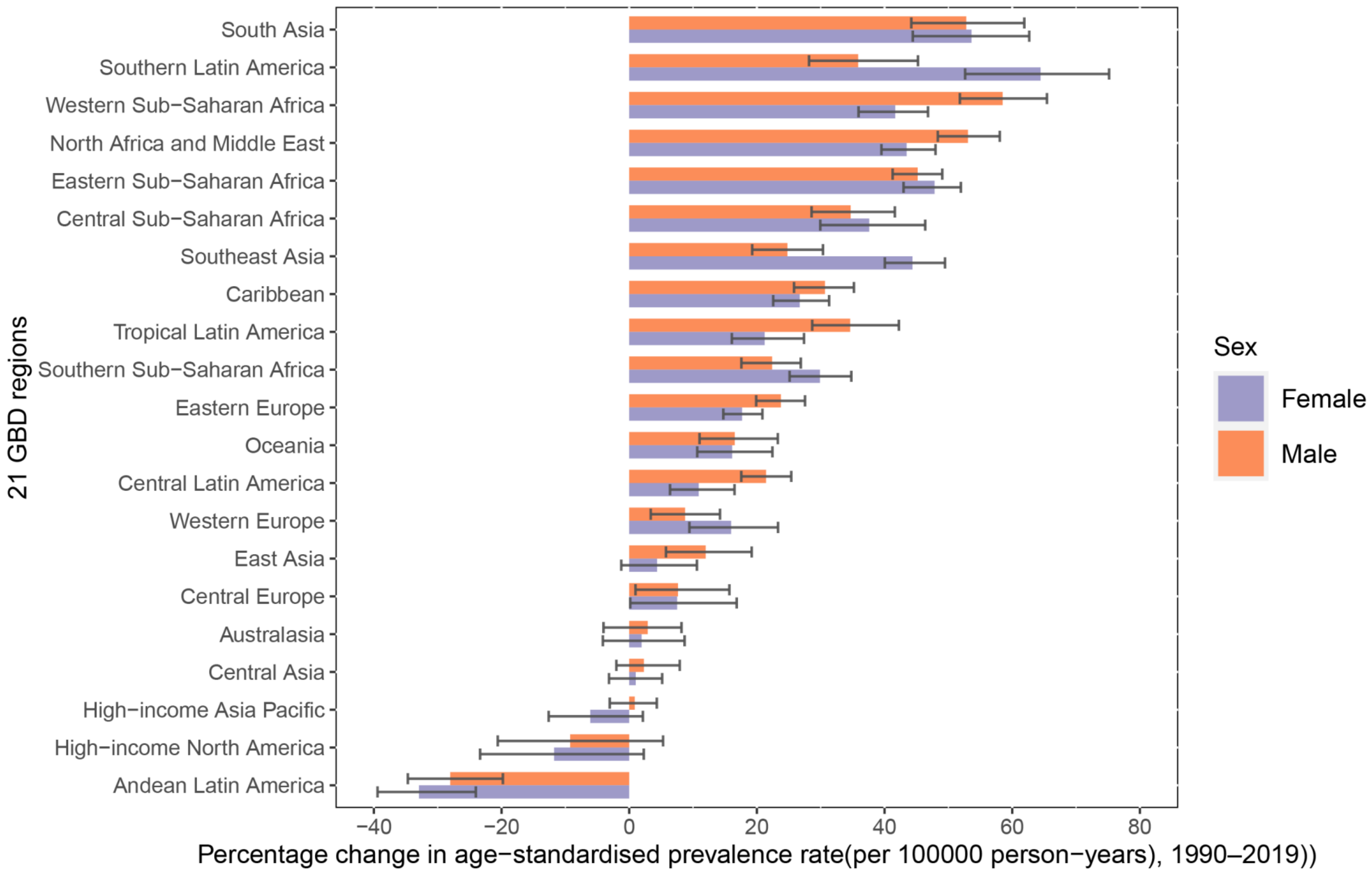

Figure S4: The percentage change in age-standardized point prevalence of appendicitis from 1990 to 2019 for 21 Global Burden of Disease regions by sex.

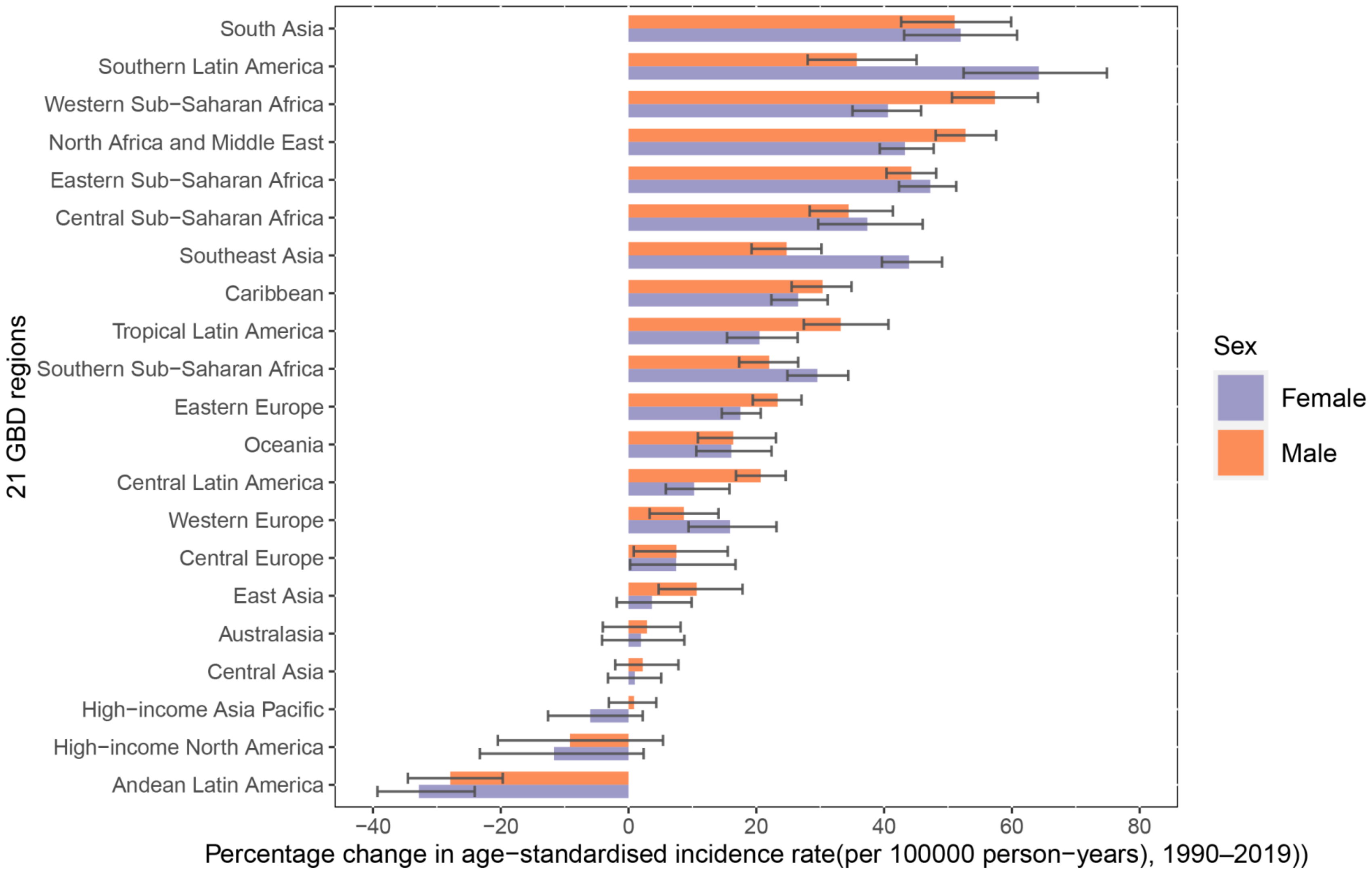

Figure S5: The percentage change in age-standardized point incidence of appendicitis from 1990 to 2019 for 21 Global Burden of Disease regions by sex.

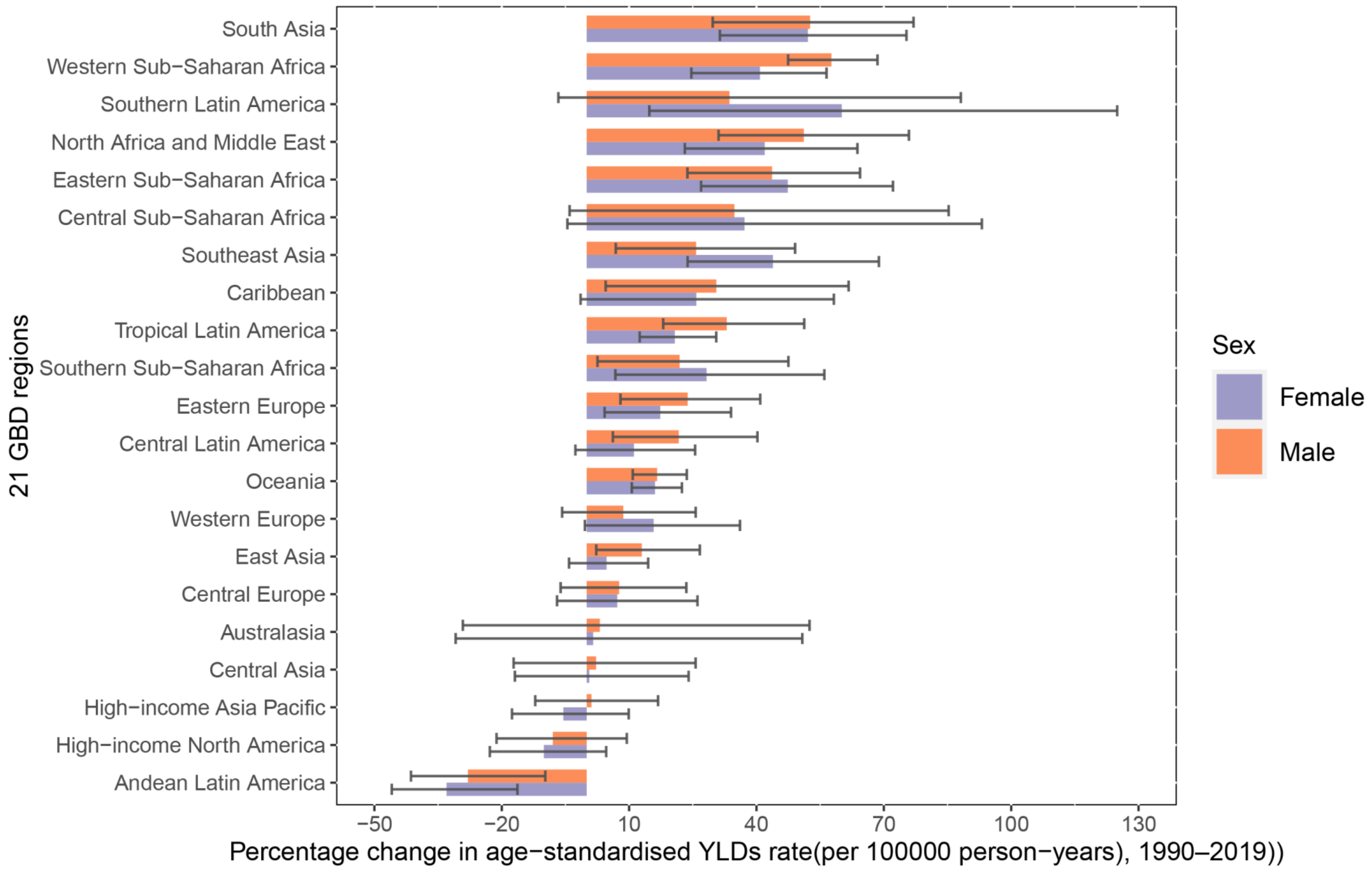

Figure S6: The percentage change in age-standardized point YLDs of appendicitis from 1990 to 2019 for 21 Global Burden of Disease regions by sex.

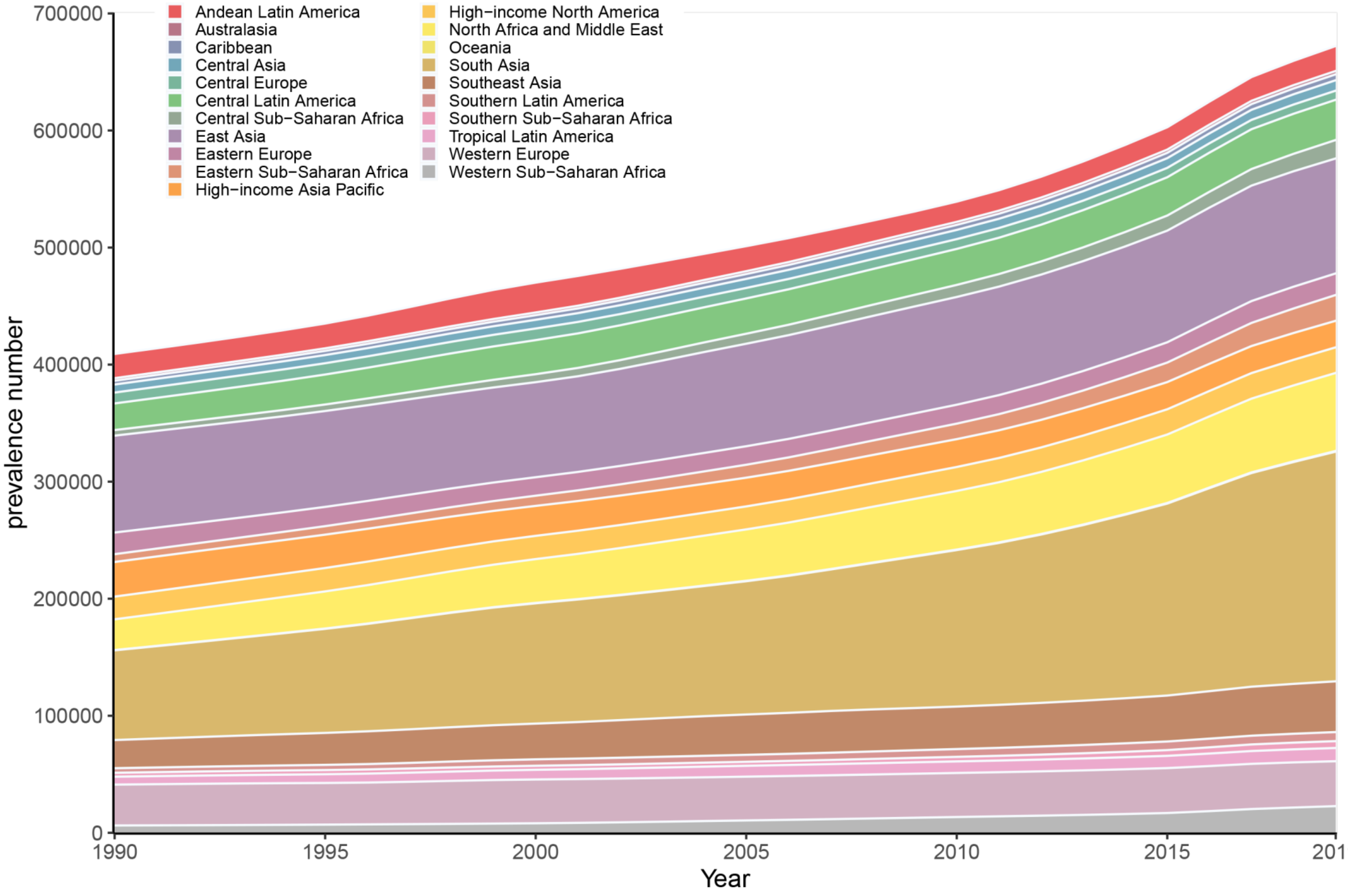

Figure S7: Number of prevalent cases of appendicitis from 1990 to 2019 for 21 Global Burden of Disease regions

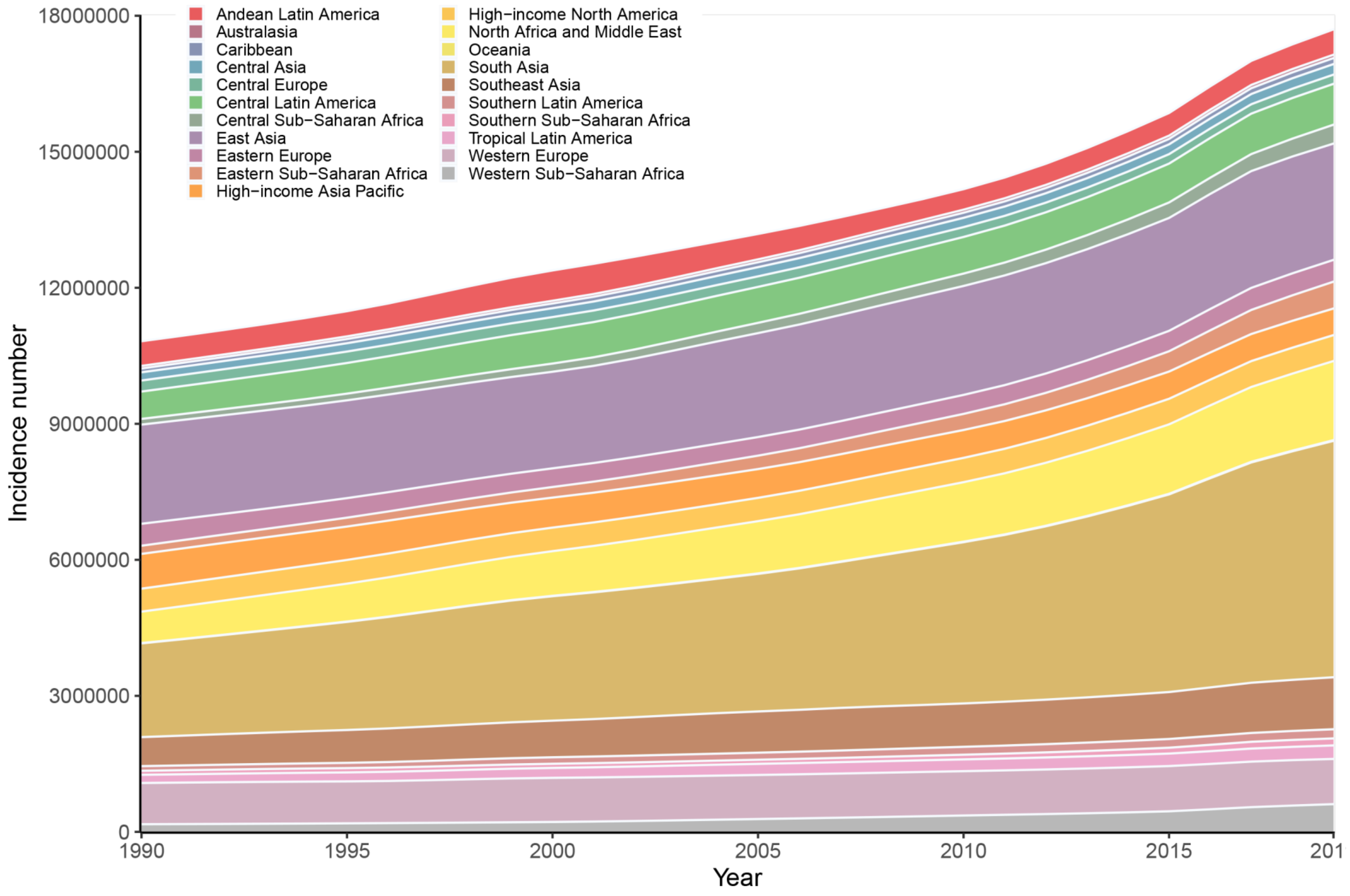

Figure S8: Number of incidence cases of appendicitis from 1990 to 2019 for 21 Global Burden of Disease regions

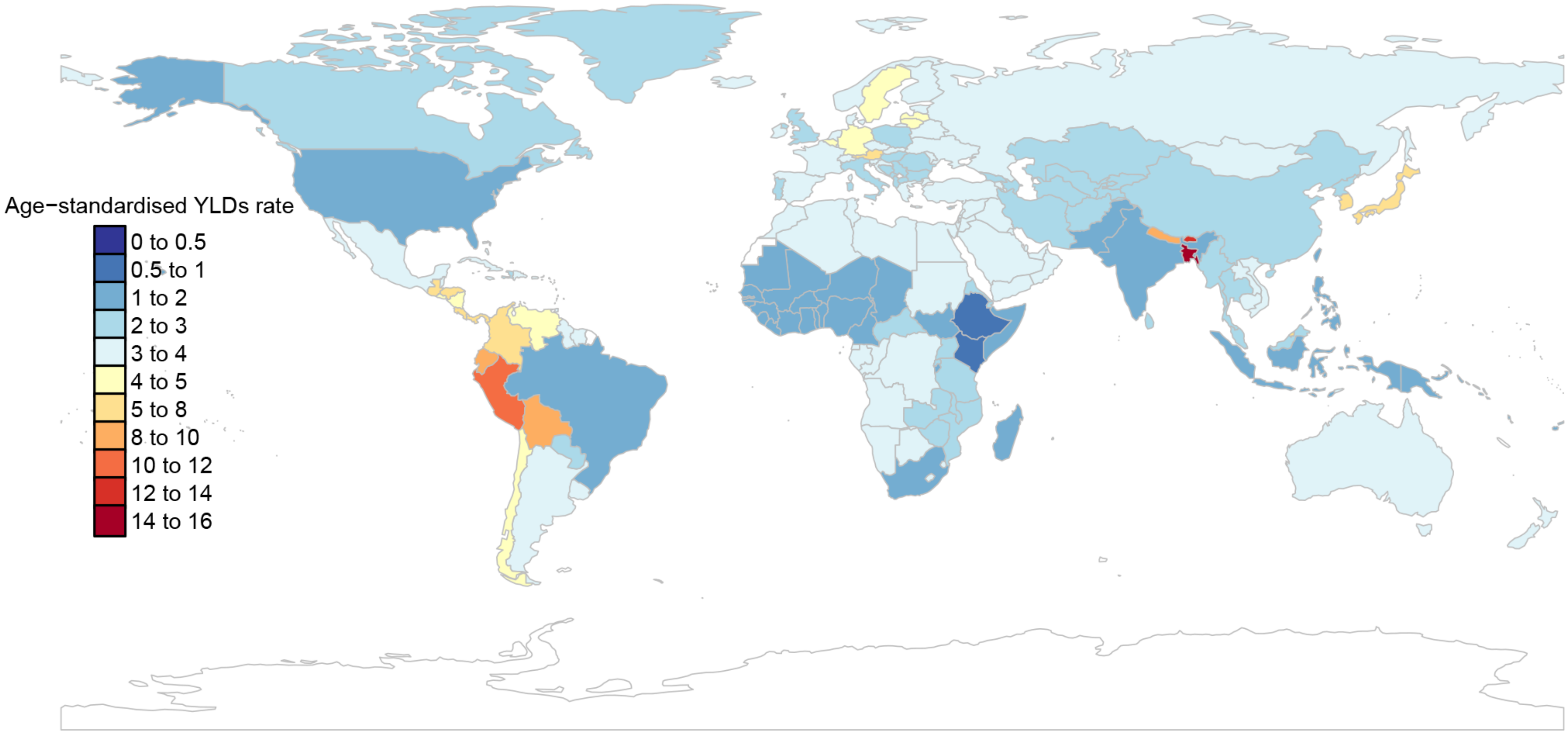

Figure S9: Age-standardized YLDs rates of appendicitis per 100 000 population in 2019, by country and territory.

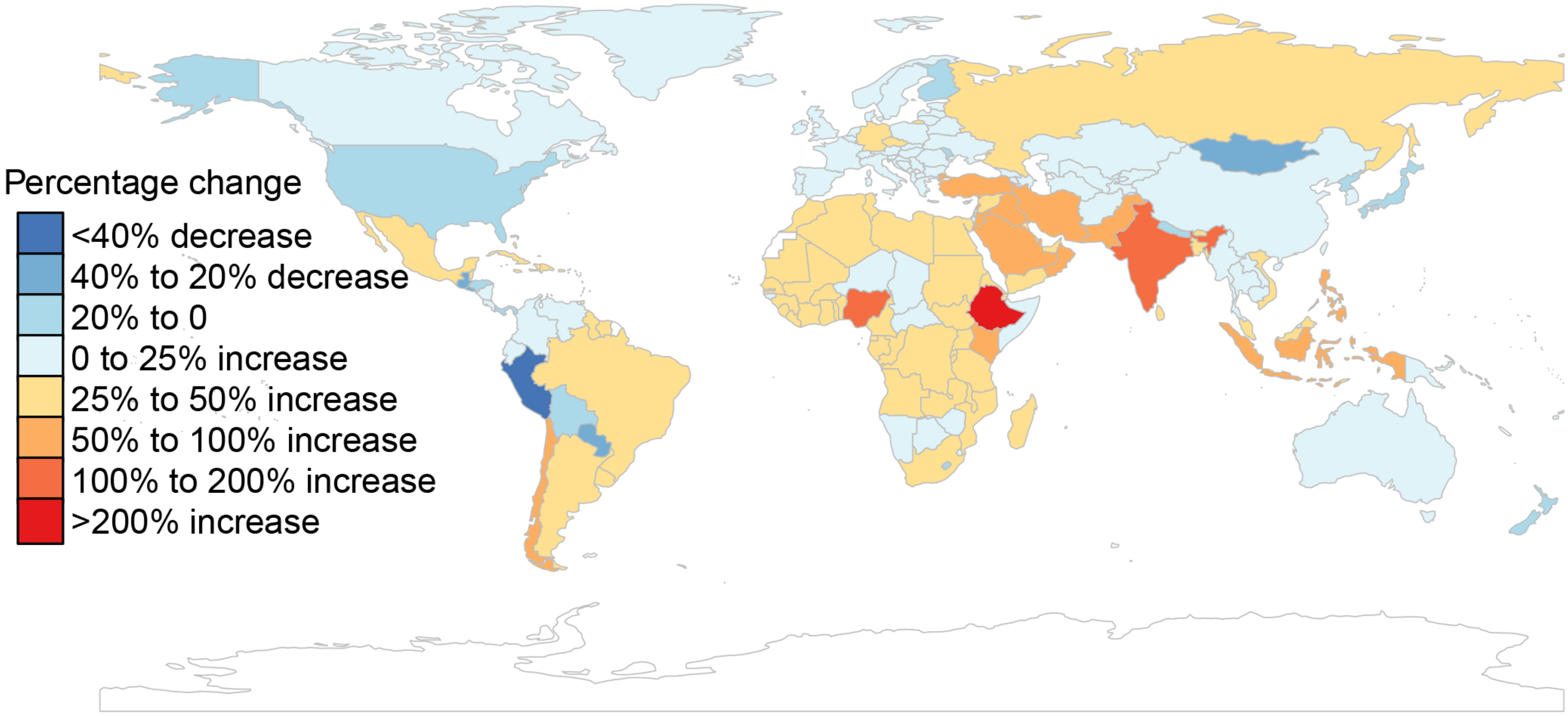

Figure S10: The percentage change in age-standardized point prevalence of appendicitis from 1990 to 2019 for country and territory.

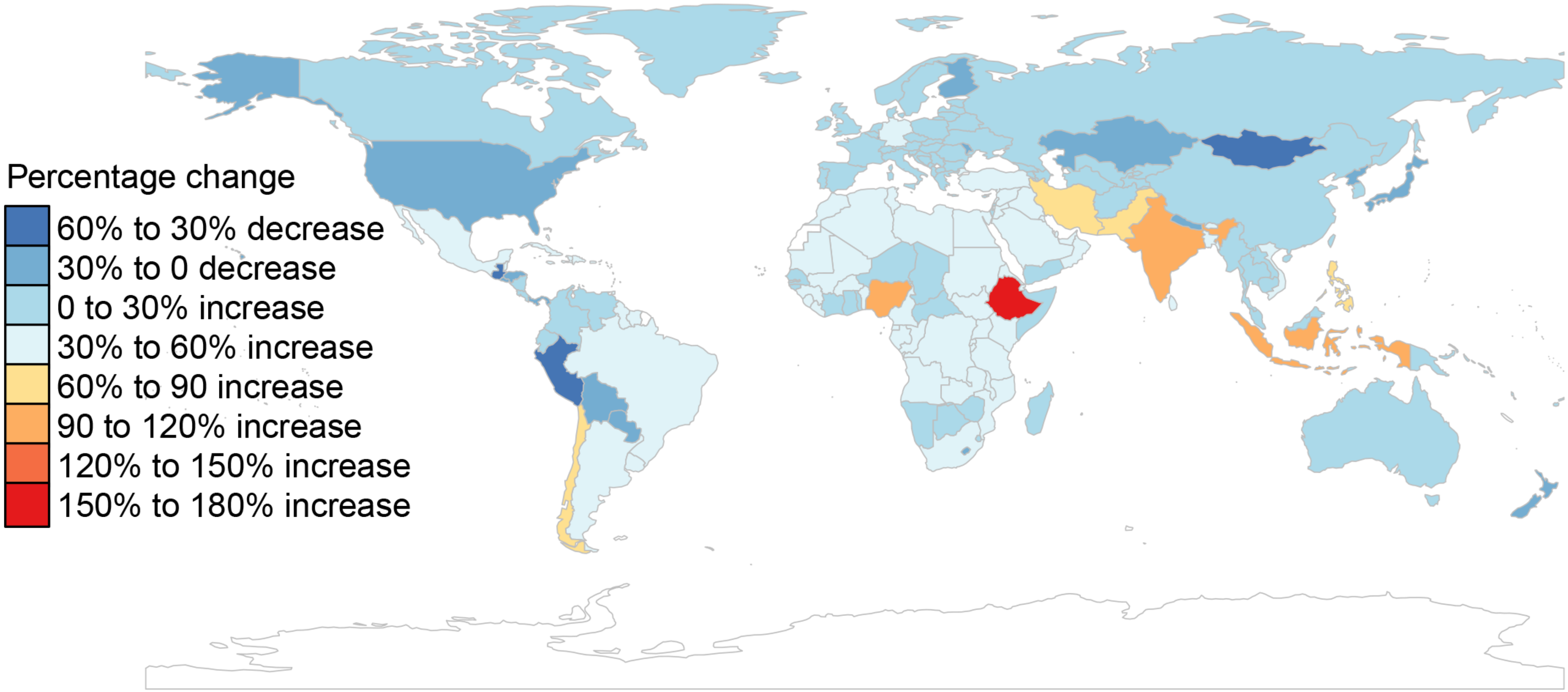

Figure S11: The percentage change in age-standardized point incidence of appendicitis from 1990 to 2019 for country and territory.

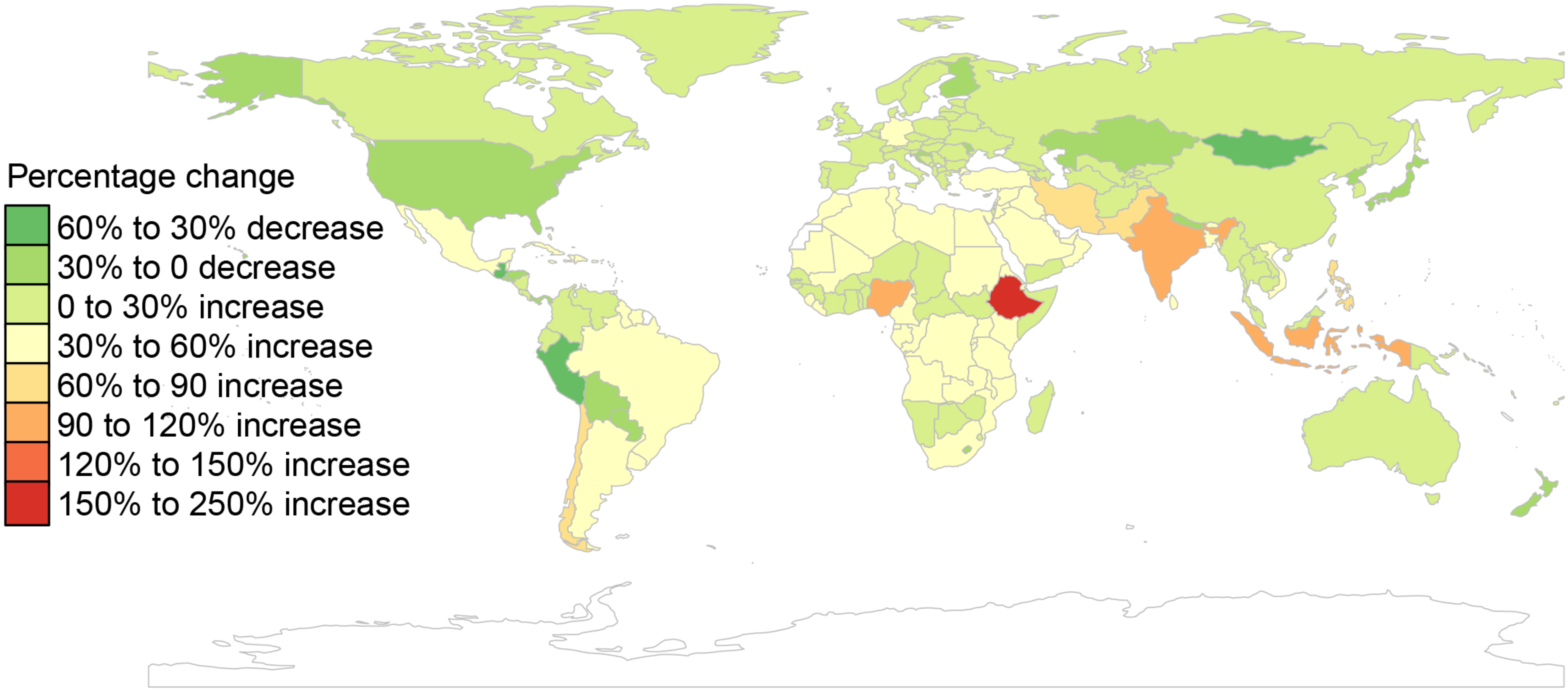

Figure S12: The percentage change in age-standardized point YLDs of appendicitis from 1990 to 2019 for country and territory.

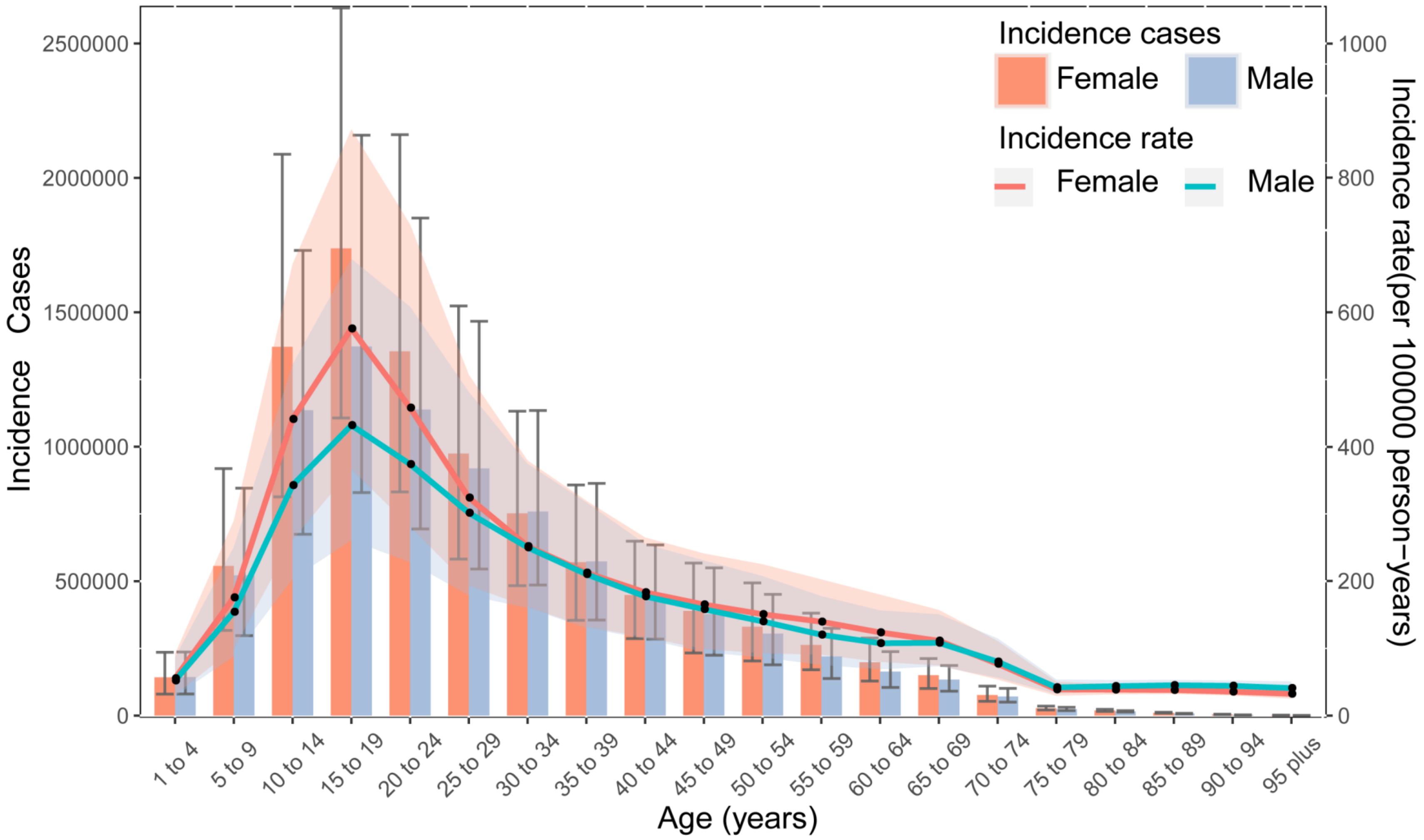

Figure S13: Global cases and age-standardized rates of incidence of appendicitis per 100 000 population by age and sex, 2019. Shading indicates the upper and lower limits of the 95% uncertainty intervals (95% UIs).

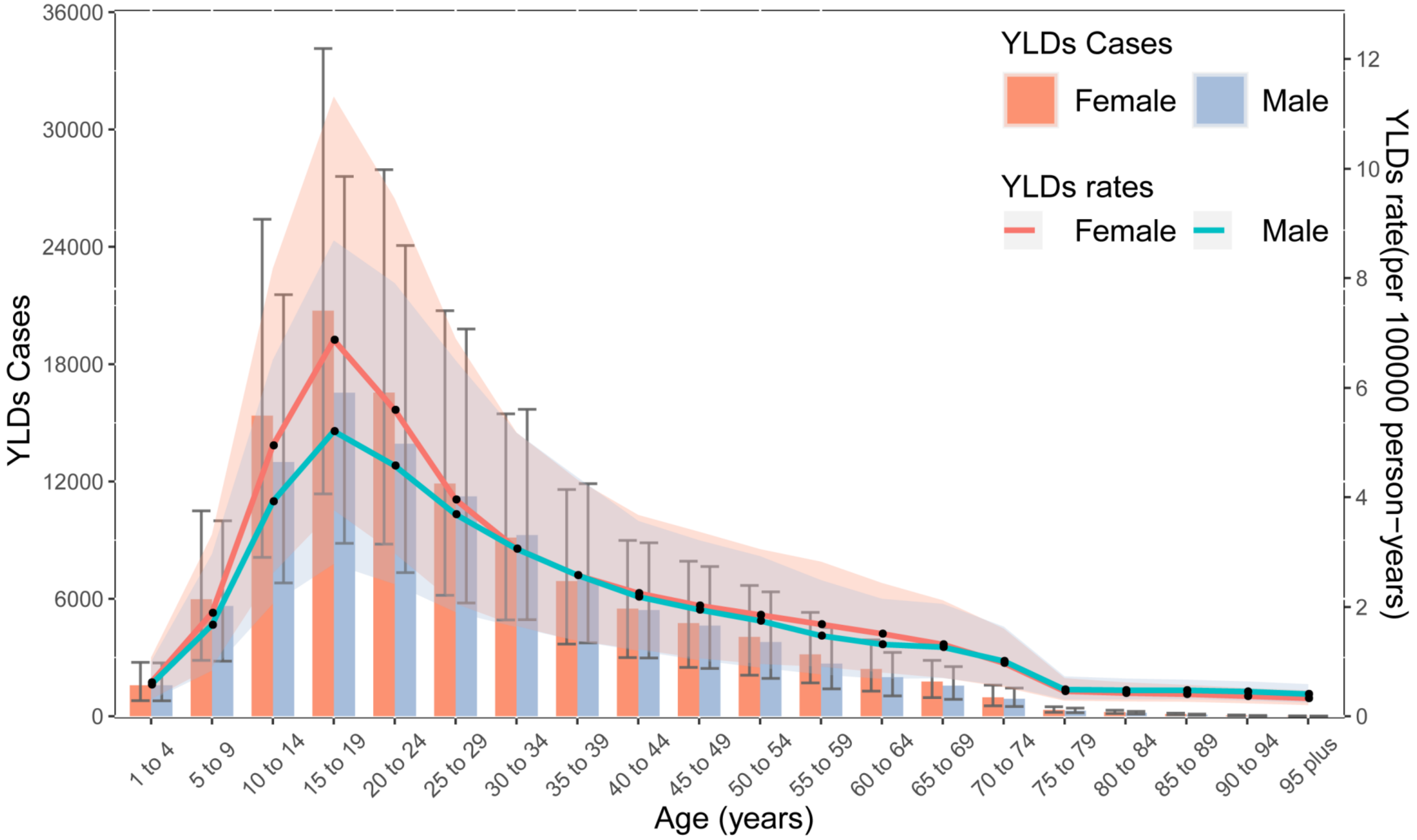

Figure S14: Global cases and age-standardized rates of YLDs of appendicitis per 100 000 population by age and sex, 2019. Shading indicates the upper and lower limits of the 95% uncertainty intervals (95% UIs).
